# Interventions to improve appropriate antibiotic prescribing in long-term care facilities: a systematic review

**DOI:** 10.1186/s12877-020-01564-1

**Published:** 2020-07-09

**Authors:** Elise Crayton, Michelle Richardson, Chris Fuller, Catherine Smith, Sunny Liu, Gillian Forbes, Niall Anderson, Laura Shallcross, Susan Michie, Andrew Hayward, Fabiana Lorencatto

**Affiliations:** 1 Department of Clinical, Educational and Health Psychology, Centre for Behaviour Change, University College London, 1-19 Torrington Place, London, WC1E 7HB UK; 2 Institute of Education (IOE), University College London, London, WC1H 0NS UK; 3 Institute of Health Informatics, University College London, London, NW1 2DA UK; 4 Health Protection Research Unit in Evaluation of Interventions, National Institute of Health Research (NIHR), London, BS8 2BN UK; 5 Institute of Epidemiology & Health, University College London, London, WC1E 7HB UK

**Keywords:** Antimicrobial stewardship, Behavioural sciences, Nursing homes, Systematic review

## Abstract

**Background:**

Overuse of antibiotics has contributed to antimicrobial resistance; a growing public health threat. In long-term care facilities, levels of inappropriate prescribing are as high as 75%. Numerous interventions targeting long-term care facilities’ antimicrobial stewardship have been reported with varying, and largely unexplained, effects. Therefore, this review aimed to apply behavioural science frameworks to specify the component behaviour change techniques of stewardship interventions in long-term care facilities and identify those components associated with improved outcomes.

**Method:**

A systematic review (CRD42018103803) was conducted through electronic database searches. Two behavioural science frameworks, the Behaviour Change Wheel and Behaviour Change Technique Taxonomy were used to classify intervention descriptions into intervention types and component behaviour change techniques used. Study design and outcome heterogeneity prevented meta-analysis and meta-regression. Interventions were categorised as ‘very promising’ (all outcomes statistically significant), ‘quite promising’ (some outcomes statistically significant), or ‘not promising’ (no outcomes statistically significant). ‘Promise ratios’ (PR) were calculated for identified intervention types and behaviour change techniques by dividing the number of (very or quite) promising interventions featuring the intervention type or behaviour change technique by the number of interventions featuring the intervention type or behaviour change technique that were not promising. Promising intervention types and behaviour change techniques were defined as those with a PR ≥ 2.

**Results:**

Twenty studies (of19 interventions) were included. Seven interventions (37%) were ‘very promising’, eight ‘quite promising’ (42%) and four ‘not promising’ (21%). Most promising intervention types were ‘persuasion’ (*n* = 12; promise ratio (PR) = 5.0), ‘enablement’ (*n* = 16; PR = 4.33) and ‘education’ (*n* = 19; PR = 3.75). Most promising behaviour change techniques were ‘feedback on behaviour’ (*n* = 9; PR = 8.0) and ‘restructuring the social environment’ (e.g. staff role changes; *n* = 8; PR = 7.0).

**Conclusion:**

Systematic identification of the active ingredients of antimicrobial stewardship in long-term care facilities was facilitated through the application of behavioural science frameworks. Incorporating environmental restructuring and performance feedback may be promising intervention strategies for antimicrobial stewardship interventions within long-term care facilities.

## Background

Long-term care facility (LTCF) residents have an increased risk of acquiring infections and of experiencing more severe disease course and outcomes [1]. For the purpose of this review LTCFs are defined as “an institution such as a nursing home that is capable of providing continuous care for older or chronically ill persons” [2]. Age-related biological factors (e.g. frailty and co-morbidity), as well as LTCF environmental factors (e.g. the frequency of contact between residents) [3], contribute to this increased risk of infection acquisition. Among the most commonly reported infections that can cause outbreaks are: chest infections, gastrointestinal infections, urinary tract infections and skin and soft tissue infections [3, 4]. Infections, such as these, are associated with high morbidity and mortality rates, re-hospitalisation and substantial health care costs [5–7]. Owing to the frequency of symptoms (e.g. fever, nausea, cough, aches, diarrhoea) that may indicate infection, antimicrobials are often prescribed to residents of LTCFs and may not always be needed (e.g. prescribed for a non-bacterial infection).

Antimicrobial resistance is a growing public health threat [8]. This is partly as a result of overuse and misuse of antimicrobials, which has been observed across multiple healthcare settings [9, 10]. Safe stewardship of antibiotics (“an organisational or healthcare-system-wide approach to promoting and monitoring judicious use of antimicrobials to preserve their future effectiveness” [11]) is essential to reducing antimicrobial resistance. This is particularly important in long-term care facilities. A census of England and Wales in 2011 showed an increasingly aging population of residents in LTCFs with 59.2% of residents aged 85 and over, 30.3% aged 75 to 84 and 10.5% aged 65 to 74 [12]. Differentiating infection from other illnesses in older adults can be particularly challenging, in part due to high rates of asymptomatic bacteriuria. Older adults are particularly vulnerable to serious side effects from antibiotic use such as *Clostridium difficile* infections [13]*.* There is evidence that up to 75% of antibiotic prescriptions are considered inappropriate in LTCFs (although inappropriate prescribing was not explicitly defined) [14, 15]. More recently, cross-sectional analyses have shown rates of inappropriate prescribing for infections such as urinary tract infections in LTCF residents at 55.9% [16]. Management of infections in LTCFs can also impact other care settings, such as the emergency department, where there are rising numbers of preventable emergency admissions in older adults [17]. Moreover, movement of residents from LTCFs to other healthcare settings such as acute hospitals can also facilitate unwanted transmission and spread of resistant organisms and infections such as *Clostridium difficile* [18, 19].

Interventions to support safe stewardship of antibiotics, sometimes referred to as antimicrobial stewardship programmes, aim to reduce the behaviours associated with antimicrobial overuse or misuse, whilst still ensuring that effective treatments are received by those who need them [20]. Examples of these programmes can include provision of recommendations for optimal antibiotic prescribing practices [21], comparison of prescribing data from one healthcare facility against others [22] and implementation of algorithms to calculate the need for antibiotics [23].

Comprehensive reviews of the effectiveness of antimicrobial stewardship programmes have been conducted across different healthcare settings, including secondary care [24]. For example, Davey and colleagues (2017) conducted a review pooling data from 221 studies that delivered antimicrobial stewardship interventions in hospitals [24]. Generally, these interventions have been found to be safe and effective [24–29] at improving appropriate antibiotic use, whilst reducing length of hospital stay and duration of antibiotic treatment [24]. However, there is substantial heterogeneity in outcomes (e.g. relative risk of antibiotic exposure = 0.56 [95% confidence interval (CI) = 0.44 to 0.70; *P* < 0.001, [30] to relative risk of the proportion of patients on antibiotics day five postadmission = 0.83 [95% CI = 0.60 to 1.14; *p* = 0.24] [31]) [24], with limited clarity as to what makes one antimicrobial stewardship programme more effective than another.

This is also the case for antimicrobial stewardship programmes in LTCFs, where previous reviews [32–36] have found that antimicrobial stewardship interventions reduce antibiotic prescribing (e.g. meta-analysis found significant reductions of antimicrobial use by 14% [95% CI = − 8 to − 20%] [34]). These reviews ranged in the number of included studies. One review included four randomised controlled trials (RCTs) [32] whereas another included 20 studies representing a broader range of study designs (e.g. quasi-experimental, pe-post test, cluster RCTs) [35]. However, there was wide variation of effects across different outcomes in different studies (e.g. odds ratio (OR) = 0.47 [95% CI 0.21 to 1.05] for antibiotic prescribing adherent to guidelines; absolute risk reduction = 0.028 [95% CI = − 0.193 to 0.249] in proportion of quinolones prescribed; weighted mean difference = − 0.49, [95% CI = -0.93 to − 0.06] for rate of antimicrobial use for suspected urinary tract infection) [32, 33]. There is limited and variable high-quality trial evidence delivering and testing effectiveness of interventions targeting antimicrobial stewardship in LTCFS and it is not clear what factors underpin this variability. Antimicrobial stewardship interventions can take many different forms, with variation in content (i.e. what is delivered), modes of delivery (i.e. how content was delivered) and targets (i.e. what behaviours and people an intervention targets). All of these factors can influence effectiveness, but their association with outcomes remain unexplored [37].

There have been calls to adopt a multidisciplinary approach to designing and evaluating antimicrobial stewardship programmes [38–40] which draw on theories, frameworks and methods in the behavioural and social sciences to understand what drives antimicrobial stewardship behaviours and how interventions have their effects (or not) [10, 38]. One approach is to apply behavioural science frameworks to specify and synthesise the content and delivery of behaviour change interventions, such as antimicrobial stewardship interventions [24, 38].

One such widely used framework is the Behaviour Change Wheel (BCW) [41] which links a model of behaviour to types of intervention that can be used. Intervention types are defined as “broad categories of means by which an intervention can change behaviour” [41]. Examples of intervention types include education, persuasion, environmental restructuring, and incentivisation. They are made up of smaller component behaviour change techniques (BCTs). BCTs are “observable, replicable and irreducible components of an intervention designed to alter or redirect causal processes that regulate behaviour” [42], and are the ‘active ingredients’ within an intervention. Examples include ‘feedback on behaviour’, ‘goal setting’ and ‘prompts/cues’. Complex interventions are typically made up of multiple, often interacting, component BCTs [39]. A taxonomy synthesising published literature on behavioural definitions outlines and defines 93 distinct BCTs [42]. These BCTs are grouped into 16 clusters representing the mechanisms through which the BCTs may change behaviour (e.g. shaping knowledge) [42]. The BCT Taxonomy was developed to enable detailed and consistent specification of the active ingredients (content) of complex behaviour change interventions. Both the BCW and the BCT Taxonomy have been used in systematic reviews to: 1) identify and categorise the components in published descriptions of interventions, and 2) examine the association between identified intervention types or BCTs and outcomes using meta-regression techniques. This enables disentanglement of intervention effects and heterogeneity to precisely identify which components of these multi-faceted, complex interventions are contributing to improved outcomes [43, 44].

The present study aimed to build upon and update the existing systematic reviews of antimicrobial stewardship programmes in LTCFs [32–36] by applying these behavioural science frameworks to specify the components of existing interventions in this context, as a basis for identifying those that are associated with improved outcomes. The specific research questions were:

1. Which behaviours are targeted by the identified interventions?
2. What intervention types and BCTs are used in existing interventions?
3. How effective are interventions to improve antibiotic prescribing in LTCFs?
4. Which intervention types and BCTs are associated with intervention effectiveness?

## Methods

This systematic review was conducted in accordance with the Preferred Reporting Items for Systematic Reviews and Meta-Analyses (PRISMA) guidelines [45]. The systematic review protocol was prospectively registered on PROSPERO (CRD42018103803).

### Search strategy and selection process

The search targeted literature investigating antimicrobial stewardship interventions in LTCFs. The search strategy was based upon a previous systematic review in this area [32]. We expanded the existing search strategy to include a wider range of study designs (e.g. quasi-experimental, prospective cohort). A multi-method search was undertaken using combined terms for antimicrobial stewardship AND long-term care facilities, plus a combination of subject heading and free text searching (see Supplement 1 for tailored search strategy). Reference list searching of existing systematic reviews identified in the search also took place. Sources included MEDLINE, EMBASE, PsycINFO, the Cochrane Library, Web of Science (core collection) and reference lists of included full text articles. The search was limited to English language. The search was run from inception until July 2018.

Title and abstract screening was conducted followed by assessment of full texts according to predefined inclusion/exclusion criteria. Eppi Reviewer software [46] was used to manage this process. Article screening were performed independently by three reviewers (EC, MR, CF). Three iterative rounds of double title/abstract screening took place to ensure application of inclusion criteria was consistent (*n* = 35 title/abstracts per round) and 97% agreement was reached. Disputes were resolved through discussion to ascertain 100% agreement and then the remaining title/abstracts were distributed and independently screened by EC, MR and CF. All full text articles were double screened by EC and CF. Initial agreement was 80% and so conflict resolution was conducted by an additional reviewer (FL) until 100% agreement was reached. The search and selection process is displayed in Fig. 2 (see Results).

### Criteria for study inclusion and exclusion

Inclusion criteria:

- Population: Studies evaluating antimicrobial stewardship interventions in LTCFs (including nursing homes, skilled nursing facilities and veteran affair nursing homes) targeting persons who could be involved in stewardship (i.e. healthcare professionals (including nurses, physicians, pharmacists, nursing assistants and managerial staff), patients and/or family members)
- Intervention: Interventions were included when targeting antimicrobial stewardship behaviours where the intervention was aimed to change antimicrobial stewardship
- Comparison: Studies using a comparator group of control/usual care or other intervention as well as studies with no comparator group (encompassing designs such as RCTs, cluster RCTs (cRCTs), quasi RCTs, interrupted time-series studies, before and after studies, cohort studies and case-control studies), as there are very few RCT studies delivering antimicrobial stewardship interventions in LTCFs
- Outcomes: Studies reporting changes in antimicrobial stewardship behaviours (e.g. changes in prescribing, adherence to prescribing guidelines) in order to assess the effect of the intervention on antimicrobial stewardship
- Primary, peer reviewed research studies (as there was limited resources within the team to explore broader literature bases)
- English language (as this was the only fluent language spoken by the research team)

Exclusion criteria:

- Studies employing a qualitative study design, or reviews reporting a qualitative data synthesis (such as meta-ethnography) as data from these studies would not allow for comparison of effect size or other outcomes that would indicate intervention effectiveness

### Data extraction and analysis

#### Data extraction

Behavioural science frameworks, specifically the BCW [41] and BCT Taxonomy [42] were used to support data extraction and categorisation of the components comprising the content of the interventions reported in the papers included in this review. These frameworks differ in their level of granularity. The BCW specifies broad types of intervention strategies to change behaviour (e.g. education, training, incentivisation), whereas the BCT taxonomy specifies the more granular active ingredients that make up these broad intervention types (e.g. goal setting, action planning, problem solving). Further, antimicrobial stewardship is an umbrella term comprising multiple actions/behaviours (e.g. identifying suspected infections, conducting diagnostic tests, adherence to prescribing guidelines, prescription of antibiotics, review and de-escalation of antibiotic prescriptions). These behaviours are performed at different time points in the care pathway, by different healthcare professional prescriber and non-prescriber roles (e.g. doctors, nurses, pharmacists, care assistants, care home managers). Therefore, we applied the TACTA principle of behavioural specification: Target, Action, Context, Timeframe, Actor (TACTA)) [47], to precisely specify the behaviours targeted by the included interventions. In summary, based on these frameworks, intervention content and behaviours were extracted and categorised into:

- A potential nine intervention types (Education, Training, Modelling, Incentivisation, Coercion, Enablement, Environmental Restructuring, Persuasion and Restriction) based on the BCW
- A potential 93 BCTs based on the BCT Taxonomy V1.
- The TACTA categories, for example: Target (‘to whom’ the behaviour effects; e.g. the infections residents have that antibiotics are prescribed for); Action (‘what’ is being targeted for change, e.g. prescribing of a specific antibiotic); Context (where the behaviour is performed e.g. a nursing home); Timeframe (‘when’ and for ‘how long’ the behaviour is performed e.g. review of antibiotic prescription weekly until the course ends); Actor (the person(s) ‘who’ are part of the intervention e.g. prescribing clinicians) [47]

A data extraction proforma was developed and piloted for this review, in accordance with Cochrane guidance [48]. Data extracted included:

1. Study characteristics (e.g. country, study design, number of service users in a LTCF, use of theory)
2. Description of the behaviour targeted by the intervention. Antimicrobial stewardship is an umbrella term comprising multiple behaviours (e.g. adherence to prescribing guidelines, prescription of antibiotics, review of antibiotic prescriptions), performed at different time points by different actors.
3. Outcomes, effectiveness, and other reported statistics (e.g. confidence intervals, *p* values)
4. Intervention components, including intervention types identified through application of the BCW [41] and BCTs coded through application of the BCT Taxonomy [42]

**Fig. 1:**
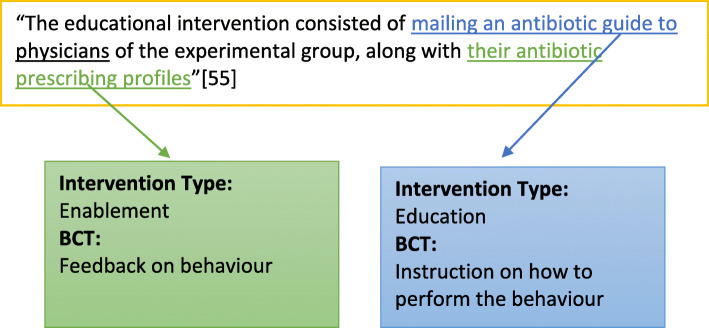
Example of intervention type and component behaviour change technique extraction and coding

Data extraction was performed by EC, with intervention components extracted by two reviewers (EC and FL) for 50% of articles. The process of extracting intervention components entailed extraction of intervention descriptions from the published paper. Descriptions were read line by line, with intervention components extracted and classified using the BCW [41] and BCT Taxonomy [42] as a coding framework (see Fig. 1 for an example). Initial inter-rater agreement was 82.6%, Cohen’s Kappa k = 0.686; 95% CI = 0.57 to 0.8; *p* < 0.0005, indicating substantial agreement [49]. Discrepancies were resolved through discussion until 100% agreement was reached.

Quality appraisal of included studies was carried out by two reviewers (SL and EC). The Cochrane risk of bias tool [48] was used to appraise studies using randomised study designs and the ROBINS-I (Risk Of Bias In Non-randomised Studies - of Interventions) [50] for studies using non-randomised study designs.

#### Analysis

Study design, outcomes and data heterogeneity prevented meta-analysis of intervention effect sizes and meta-regression to assess associations between intervention components and intervention effectiveness. We therefore adapted the method used by Gardner et al. [51] and Martin et al. [52] to identify potentially promising intervention components. This approach provides a descriptive measure in which to summarise the data, with findings indicative of potential trends between intervention components and outcomes.

Interventions were grouped into three categories according to their potential to change antibiotic stewardship behaviours. Potential was judged according to whether a significant change in behaviour was reported following intervention; assessed by the reported effect size, confidence intervals and/or *p* values (significance defined as a p value of ≤0.05) of the intervention. Interventions were classified as one of the following:

- ‘Very promising’. A*ll* antibiotic stewardship behavioural outcomes assessed showed significant improvement following intervention within the intervention group and/or behaviour change was greater than observed in the comparator group.
- ‘Quite promising’. A*t least one* antibiotic stewardship behavioural outcome assessed showed significant change following intervention within the intervention group and/or behaviour change was greater than observed in the comparator group (e.g. a significant change to rates of prescription of one antibiotic but not another).
- ‘Not promising’. The intervention resulted in *no significant change* to antibiotic stewardship behavioural outcomes within the intervention group and/or behaviour change was not greater than observed in the comparator group.

Descriptive statistics were used to summarise the number of intervention types and BCTs in each intervention. The relationship between individual BCTs or intervention types with intervention promise was judged by deriving ‘promise ratios’, calculated as:

$$ \frac{No. of` very' or` quite\ promising' interventions\ featuring\ intervention\ type\ or\  BCT}{No. of` not\ promising' interventions\ featuring\ intervention\ type\ or\  BCT} $$

This promise ratio indicates the potential contribution of intervention types or BCTs to intervention promise [52]. A promise ratio of ≥ 2 was judged as indicating an intervention type or BCT as likely promising in this context (i.e. the intervention type or BCT are used in twice as many promising as not promising interventions). Promise of an intervention type or BCT was also only considered when the intervention type or BCT was present in at least two interventions in total. In instances where intervention types or BCTs were only used in (two or more) interventions classified as promising (i.e. there was no not promising comparison to derive a promise ratio), the number of interventions in which they were used was reported instead of a promise ratio. Inferential statistics to explore associations between intervention promise and the number of intervention types and BCTs used within an intervention was planned. Sensitivity analysis to explore the relationship between risk of bias score and intervention promise was also planned.

## Results

**Fig. 2:**
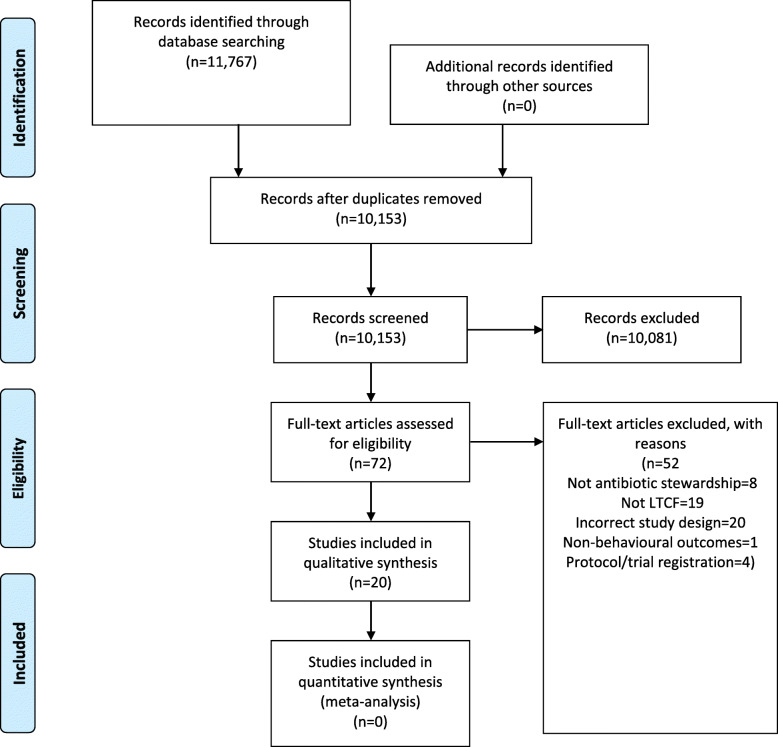
PRISMA flow diagram

The search retrieved a total of 10,153 articles (with duplicates removed). Titles and abstracts were screened, resulting in 72 full texts to assess. Following assessment of full texts, 20 papers met inclusion criteria, reporting 19 interventions (Fig. 2). As two studies [53, 54] reported the same intervention, data are presented in terms of number of studies (x/20) or number of interventions (x/19).

### Study characteristics

Study characteristics are summarised in Table 1. The majority of studies were conducted in North America (Canada (*n* = 3), United States (US) (*n* = 14)). Nursing homes were the most frequent LTCF setting (*n* = 11). Across the 20 studies a total of 72 LTCFs were exposed to an intervention. Of studies that reported a control arm (11/20 studies; 9/19 interventions), there were a total of 71 control LTCFs included. An average of 1435 LTCF residents (range = 24–4217) were included in the analysis (reported in 9/20 studies; 8/19 interventions). Twelve studies did not clearly report the number of healthcare professionals who received intervention or control conditions. Of the seven studies (7/19 interventions) that did, there was a mean of 57 (range = 10–206) healthcare professionals. A range of study designs were used: RCT (*n* = 1), cRCT (*n* = 4), quasi-experimental (*n* = 7), cross-sectional (n = 1), pre-post test designs (*n* = 8), observational (n = 1), interventional (n = 1) and clinical demonstration project (n = 1). Eleven studies (10/19 interventions) did not specify the timeframe for outcome data collection. For the nine that did, follow-up data collection occurred at 3 months (*n* = 2), 4 or 5 months (n = 2), 6 months (*n* = 3), 11 months (n = 1) and 30 months (n = 1) [23, 53–71].

**Table 1:** Study characteristics and intervention effects

First author/ Country	Design [sample size^**a**^]	Measurement method of behaviour	Antibiotics Assessed	Comparison/ control	Behavioural outcomes	Reported statistics	95% CI	Intervention promise	Risk of Bias
Monette 2007/ Canada [55]	cRCT [8 LTCFs]	Not reported	Not reported	Usual care	Adherence to guidelines post-intervention 1(3-month period)	OR = 0,47	0.21, 1.05	Very	Unclear
					Adherence to guidelines post-intervention 2 (3-month period)	OR = 0.36	0.18, 0.73		
					Adherence to guidelines follow up (3-month period)	OR = 0.48	0.23, 1.02		
Naughton 2001/ US [56]	RCT [10 LTCFs]	Form completion	Not reported	Pre-intervention	Antibiotic use consistent with guideline	OR = NR	NR	Not	Low
Jump 2013/ US [57]	Clinical Demonstration Project [1 LTCF]	Chart review	Not reported	None	Relationship between referral source (LTCF or ID consultation service) and likelihood of needing an intervention (stewardship behaviour) [adjusted OR]	OR = 0.4	0.19, 0.78	Very	Critical
McMaughan 2016/ US [58]	Pre-post test with comparison [12 LTCFs]	Chart review	Not reported	Comparison-no description provided	Relationship between group allocation and prescription being written: High intensity decision aid training	OR = 0.77	0.32, 1.86	Not	Serious
					Relationship between group allocation and prescription being written: Low intensity decision aid training	OR = 1.19	0.47, 3.01		
					Pre/post intervention comparison: high intensity decision aid training	OR = 0.79	0.33, 1.88		
					Pre/post intervention comparison: Low intensity decision aid training	OR = 0.63	0.25, 1.60		
Van Buul 2015/ Netherlands [59]	Quasi-experimental [10 LTCFs]	Form completion	Not reported	Comparison-no description provided	Overall appropriateness of antibiotic prescribing	OR = 0.76	0.43, 1.34	Not	Serious
					Appropriateness of antibiotic prescribing for UTI	OR = 0.74	0.39, 1.40		
					Appropriateness of antibiotic prescribing for RTI	OR = 0.95	0.39, 2.33		
Petterson 2011/ Sweden [60]	cRCT [46 LTCFs]	Survey/ questionnaire	Quinolones Nitrofurantoin	Comparison-no description provided	Proportion of quinolones prescribed for lower UTI	Β = 0.028	− 0.193, 0.249	Quite	High
					Proportion of antibiotics prescribed	Β = 0.124	− 0.228, − 0.019		
					Proportion of nitrofurantoin prescribed for lower UTI	Β = − 0.077	−0.242, 0.088		
Schwartz 2007/ US [61]	Quasi-experimental [1 LTCF]	Chart review	Not reported	None	Effect of intervention on incidence of antimicrobial days	Β = −0.04	NR	Very	Moderate
					Effect of intervention on incidence of antimicrobial starts	Β = −0.05	NR		
Loeb 2005/ Canada & US [23]	cRCT [20 LTCFs]	Form completion	Not reported	Usual care	Rate of antimicrobial use for suspected UTI	t = − 0.49	−0.93, − 0.06	Quite	High
					Total antimicrobial use between the intervention and usual care groups	t = − 0.37	−1.17, 0.44		
Kassett 2016/ Canada [62]	Pre-post test [1 LTCF]	Medical record review	Amikacin Amoxicillin Amoxicillin– clavulanic acid Ampicillin Ceftriaxone Ciprofloxacin Gentamicin Levofloxacin Meropenem Nitrofurantoin Norfloxacin Piperacillin and tazobactam Tobramycin Trimethoprim (TMP) Trimethoprim–sulfamethoxazole (TMP-SMX)	None	UTI rate (an index of overall antibiotic use)	t = 1.255 d = 0.535	−0.003, 0.013	Quite	Serious
					Prescribed days of therapy	t = 2.293 d = 0.978	0.003, 0.066		
					Actual days of therapy	t = 2.902 d = 1.237	0.011, 0.065		
					Ciprofloxacin rate (number of ciprofloxacin prescriptions in a given month per LTCF unit occupancy for that month)	t = 3.79 d = 1.616	0.003, 0.012		
					Ciprofloxacin proportion (number of ciprofloxacin prescriptions in a given month per UTI case)	t = 3.809 d = 1.624	0.064, 0.216		
Doernberg 2015/ US [63]	Quasi-experimental [3 LTCFs]	Chart review	Fluoroquinolones Nitrofurantoin Trimethoprimsulfamethoxazole Cephalexin Amoxicillin +/− clavulanate	None	Antibiotic starts for UTI (per 1000 resident-days)	IRR = 0.94	0.92, 0.97	Very	Serious
					All antibiotic starts (per 1000 resident-days)	IRR = 0.95	0.92, 0.98		
Zimmerman 2014/ US [64]	Quasi-experimental [12 LTCFs]	Infection control log review	Not reported	Comparison-no description provided	Overall intervention effectiveness	IRR = 0.71	0.56, 0.90	Very	Moderate
					Antibiotic prescribing (prescriptions per resident-day) [adjusted model]	IRR = 0.86	0.79, 0.95		
Fleet 2014/ UK [65]	cRCT (pilot) [30 LTCFs]	Not reported	Amoxicillin Co-amoxiclav Flucloxacillin Trimethoprim Clarithromycin Cefalexin Erythromycin Nitrofurantoin Ciprofloxacin Doxycycline Other	Control- no description provided	Pre- and post-intervention point prevalence of systemic antibiotic prescribing (per 100 residents) for treatment of infection - Intervention Group	EPR = 1.01	0.81, 1.25	Quite	Low
					Pre- and post-intervention point prevalence of systemic antibiotic prescribing (per 100 residents) for treatment of infection - Control Group	EPR = 1.11	0.87, 1.41		
					Total antibiotic consumption (defined daily does (DDDs)/1000 residents/day (DRD)) - Intervention group	% decrease = 4.9% (3.25 DRD)	1.0, 8.6%		
					Total antibiotic consumption (defined daily does (DDDs)/1000 residents/day (DRD)) - Control group	% increase = 5.1% (2.24 DRD)	0.2, 10.2%		
Furuno 2014/ US [66]	Pre-post test [1 LTCF]	Semi-structured interview and chart review	Aminoglycosides Carbapenems Cephalosporins Fluoroquinolones Vancomycin Trimethoprim-sulfamethoxazole Tetracyclines Nitrofurantonin Metronidazole Other	None	Appropriate empirical antibiotic prescribing	% Increase: 32 to 45%	NR	Not	Serious
Gugkaeva 2012/ US [67]	Phase 1 observational Phase 2 interventional [1 LTCF]	Medical record review	Not reported	None	Cases where antibiotics were prescribed inappropriately (comparison pre and post intervention implementation)	% Decrease: 40 to 21%	NR	Quite	Serious
Hutt 2006/ US [68]	Pre-post test [2 LTCFs]	Nursing home record review: - A modified Barthel Index -Cognitive Performance Scale -NHAP Severity Index -Measures of intervention dose	Not reported	Control- no description provided	Compliance to guideline: Timely antibiotics - Intervention Group [change in percentage points]	−9	NR	Quite	Serious
					Compliance to guideline: Timely antibiotics - Control Group [change in percentage points]	−53	NR		
					Compliance to guideline: Appropriate antibiotics - Intervention Group [change in percentage points]	18	NR		
					Compliance to guideline: Appropriate antibiotics - Control Group [change in percentage points]	−13	NR		
					Average total compliance to guidelines score – intervention group [change in percentage points]	5	NR		
					Average total compliance to guidelines score – intervention group [change in percentage points]	−5	NR		
Linnebur 2011/ US [54]	Quasi-experimental [16 LTCFs]	Medical record review	Levofloxacin Azithromycin Other	Control-no description provided	Providing antibiotics within 4 h - Intervention group [change in % from baseline to 2 years]	57 to 75%	NR	Quite	Critical
					Providing antibiotics within 4 h - Control group [change in % from baseline to 2 years]	38 to 31%	NR		
					Adherence to optimal antibiotic use - Intervention group [change in % from baseline to 2 years]	60 to 66%	NR		
					Adherence to optimal antibiotic use - Control group [change in % from baseline to 2 years]	32 to 39%	NR		
					Receipt of antibiotics for 10 to 14 days - Intervention group [change in % from baseline to 2 years]	27 to 13%	NR		
					Receipt of antibiotics for 10 to 14 days - Control group [change in % from baseline to 2 years]	24 to 19%	NR		
Rahme 2016/ US [69]	Pre-post test [1 LTCF]	Form completion (inventory usage reports)	Fluoroquinolone Nitrofurantoin Ciprofloxacin Levofloxacin Moxifloxacin Penicillin Cephalosporin Macrolide Tetracycline Sulfonamide	None	Total antibiotic use (daily defined dose (DDD) per 1000 resident days (RD) [pre/post intervention percentage change]	−11.68%	−0.44, −18.97	Quite	Low
					Penicillin use (daily defined dose (DDD) per 1000 resident days (RD) [pre/post intervention percentage change]	+ 10.23%	−5.15, 1.21		
					Cephalosporins use (daily defined dose (DDD) per 1000 resident days (RD) [pre/post intervention percentage change]	−24.49%	−0.15, 6.41		
					Macrolides use (daily defined dose (DDD) per 1000 resident days (RD) [pre/post intervention percentage change]	−25.63%	−1.25, 4.50		
					Tetracyclines use (daily defined dose (DDD) per 1000 resident days (RD) [pre/post intervention percentage change]	−14.29%	−3.10, 9.48		
					Fluoroquinolones use (daily defined dose (DDD) per 1000 resident days (RD) [pre/post intervention percentage change]	−16.20%	−2.38, 6.78		
					Sulfonamides use (daily defined dose (DDD) per 1000 resident days (RD) [pre/post intervention percentage change]	+ 9.45%	−2.02, 0.30		
					Nitrofurantoin use (daily defined dose (DDD) per 1000 resident days (RD) [pre/post intervention percentage change]	−25.34%	−0.34, 1.33		
					Ciprofloxacin use (daily defined dose (DDD) per 1000 resident days (RD) [pre/post intervention percentage change]	−38.7%	0.58, 4.9		
					Levofloxacin use (daily defined dose (DDD) per 1000 resident days (RD) [pre/post intervention percentage change]	+ 9.09%	−3.21, 2.09		
					Moxifloxacin use (daily defined dose (DDD) per 1000 resident days (RD) [pre/post intervention percentage change]	−5.88%	−0.32, 0.34		
Smith 2016/ US [70]	Pre-post test [1 LTCF]	Chart review	Vancomycin	None	Compliance with vancomycin level monitoring	% Increase: 71–85%	NR	Quite	Low
					Vancomycin trough levels in therapeutic range	% Increase: 63.3–70.5%	NR		
Hutt 2011/ US [53]	Quasi-experimental [16 LTCFs]	Chart review	Not reported	Control- no description provided	Year 1 Adherence to guideline for treating stable residents in the NH	M = 95 [approx.]	NR	Quite	Serious
					Year 2 Adherence to guideline for treating stable residents in the NH	M = 98 [approx..]	NR		
Zabarsky 2008/ US [71]	Quasi-experimental [1 LTCF]	Not reported	Not reported	None	3 months Pre-intervention: rate of asymptomatic bacteria treated [per 1000 patient-days]	IRR = 1.7	1.1, 2.6	Very	Moderate
					6 months Post-intervention: rate of asymptomatic bacteria treated [per 1000 patient-days]	IRR = 0.6	0.4, 1.0		
					7 to 30 months Post-intervention: rate of asymptomatic bacteria treated [per 1000 patient-days]	IRR = 0.3	0.2, 0.4		
					3 months Pre-intervention: total antimicrobial days of therapy [per 1000 patient-days]	167.7	NR		
					6 months Post-intervention: total antimicrobial days of therapy [per 1000 patient-days]	117.4	NR		
					7 to 30 Months post-intervention" total antimicrobial days of therapy [per 1000 patient-days]	109.0	NR		

*IRR* Incidence rate ratios; d-Cohen’s d effect size, *OR* Odds ratio, *M* Mean, *EPR* Estimated prevalence rate, *t* t-test statistic; −Beta coefficient, *UTI* Urinary tract infection, *NR* Not reported, *CI* confidence interval

^**a**^Sample size reports upon the sample included in the analysis only

The majority of studies did not explicitly report the use of theory to inform the design of their intervention (19/20 studies). Only one study [53] reported using a theory (Roger’s Theory of Innovation Diffusion). Two studies reported use of frameworks (Participatory Action Research [59] and Agency for Healthcare Research and Quality’s TeamSTEPPS® patient safety model [58]) to support the development, delivery or implementation of the intervention.

### Which behaviours were targeted by interventions?

Table 2 lists studies categorised by target, action, context, timeframe and actor. We found that the behaviours targeted by the included interventions (*n* = 19) were not fully specified and described. The most common targets were urinary tract infections (*n* = 12), respiratory tract infections (*n* = 7) and skin and soft tissue infections (*n* = 4). The most common action (behaviour) to be changed by antimicrobial stewardship was antibiotic prescribing (*n* = 16), commonly within the context of nursing homes (*n* = 11) and less commonly within veteran affairs nursing home facilities (*n* = 2). Timeframe (time over which the behaviour is performed, such as reviewing/discontinuing antibiotics after a specified, evidence-based time) was not clearly specified for any intervention. The actor (i.e. the person/people who were part of the intervention) was not always specified. The majority of interventions targeted healthcare professional behaviours (*n* = 13), including physicians, nursing staff and pharmacists. There were instances where interventions were also delivered to patients/residents (n = 2) and/or family member’s (*n* = 3), with the aim of increasing knowledge of antimicrobial stewardship programmes or decreasing risks of overuse of antibiotics or general quality improvement.

**Table 2:** Studies categorised by target, action, context, timeframe and actor

Study	Target (whom)	Action (behaviour)	Context	Timeframe	Actor (whose behaviour)									
					Physicians	Nurses	Physician Assistants	Nurses Assistants	Pharmacists	Medical Directors	Patients	Family Members	Infection Control Practitioners	Other Unspecified
Monette 2007 [55]	UTI LRTI SSTI	Antibiotic prescribing	LTCF	Unspecified	–	✓	–	–	–	–	–	–	–	–
Petterson 2011 [60]	UTI	Antibiotic prescribing	Nursing home	Unspecified	✓	✓	–	–	–	–	–	–	–	–
Loeb 2005 [23]	UTI	Antibiotic prescribing	Nursing home	Unspecified	✓	✓	✓	–	–	–	–	–	–	–
Naughton 2001 [56]	NHAP	Antibiotic prescribing	Skilled nursing facility	Unspecified	✓	✓	–	✓	–	–	–	–	–	–
McMaughan 2016 [58]	UTI	Antibiotic prescribing	Nursing homes	Unspecified	–	–	–	–	–	–	–	–	–	✓
Schwartz 2007 [61]	UTI	Antibiotic prescribing	LTCF	Unspecified	✓	✓	–	–	–	–	–	–	–	✓
Kassett 2016 [62]	UTI	Antibiotic prescribing	LTCF	Unspecified	✓	✓	–	–	✓	–	✓	✓	–	–
Doernberg 2015 [63]	UTI	Antibiotic prescribing	LTCF	Unspecified	–	–	–	–	–	–	–	–	✓	–
Fleet 2014 [65]	LRTI SSTI UTI	Antibiotic prescribing	Nursing home	Unspecified	–	✓	–	–	–	–	–	–	–	–
Furuno 2014 [66]	Unspecified	Antibiotic prescribing	Skilled nursing facility	Unspecified	–	–	–	–	–	–	–	–	–	✓
Gugkaeva 2012 [67]	Unspecified	Antibiotic prescribing	Nursing home	Unspecified	–	–	–	–	–	–	–	–	–	✓
Hutt 2006 [68]	NHAP	Antibiotic prescribing	Veterans nursing home	Unspecified	✓	✓	–	✓	–	–	–	–	–	–
Linneburr 2011 [54]	LRTI	Adherence to guidelines	Nursing homes	Unspecified	✓	–	✓	✓	–	✓	–	–	–	–
Rahme 2016 [69]	UTI SSRI RTI	Antibiotic prescribing	LTCF	Unspecified	–	–	–	–	–	–	–	✓	–	✓
Hutt 2011 [53]	LRTI	Adherence to guidelines	Nursing home	Unspecified	✓	✓	✓	–	–	✓	–	–	–	–
Jump 2013 [57]	Unspecified	Enacting stewardship role	Veteran Affairs community living centre	Unspecified	–	–	–	–	–	–	–	–	–	✓
Van Buul 2015 [59]	UTI RTI	Antibiotic prescribing	Nursing home	Unspecified	✓	✓	–	–	–	–	–	–	–	–
Zimmerman 2014 [64]	UTI SSTI RTI	Antibiotic prescribing	Nursing home	Unspecified	–	–	–	–	–	–	✓	✓	–	✓
Smith 2016 [70]	Unspecified	Monitoring of AKI and vancomycin trough levels	Nursing home	When indicated	–	–	–	–	✓	–	–	–	–	–
Zabarsky 2008 [71]	UTI	Antibiotic prescribing	Nursing home	Unspecified	✓	✓	✓	–	–	–	–	–	–	✓

*TACTA* Target, Action, Context, Timeframe, Actor UTI-Urinary Tract Infection, *LRTI* Lower Respiratory Tract Infection; RTI-Respiratory Tract Infection; SSTI- Skin and Soft Tissue Infection; NHAP-Nursing Home Acquired Pneumonia

### What intervention types and behaviour change techniques (BCTs) are used in existing interventions?

The frequency of intervention types and BCTs, along with definitions and examples are presented in Table 3. The number and type of intervention types and BCTs identified within each intervention are presented in Supplement 2.

**Table 3:** Intervention types - definitions, examples, frequency and association with intervention outcomes

Intervention Type	Definition^**a**^	Example	No. used in very promising intervention	No. used in quite promising interventions	No. used in not promising interventions	Total no. of times used across all interventions	Promise ratio
**Enablement**	*Increasing means/ reducing barriers to increase capability (beyond education or training) or opportunity (beyond environmental restructuring)*	Providing feedback on prescribing e.g. sending prescribing profiles for the previous 3 months to prescribers at intervention sites [55]	7	8	4	19	3.75
**Education**	*Increasing knowledge or understanding*	Guidelines stating the recommended empirical antibiotic to be administered [61]	7	6	3	16	4.33
**Training**	*Imparting skills*	Delivering training sessions on how to use algorithms to support antibiotic prescription decision making [68]	3	4	2	9	3.5
**Modelling**	*Providing an example for people to aspire to or imitate*	Training sessions providing hypothetical case scenarios demonstrating the behaviour [68]	1	3	0	4	–
**Environmental Restructuring**	*Changing the physical or social context*	Assigning a team member to a new role [54]	6	7	4	17	3.25
**Incentivisation**	*Creating an expectation of reward*	Payment to intervention facilities to incentivise compliance to guidelines [53]	0	1	0	1	–
**Restriction**	*Using rules to reduce the opportunity to engage in the target behaviour (or to increase the target behaviour by reducing the opportunity to engage in a competing behaviour)*	Enforcing mandatory attendance to training on antimicrobial prescribing [53]	0	1	0	1	–
**Persuasion**	*Using communication to induce positive or negative feelings or stimulate action*	Using credible sources (such as colleagues perceived to be experts in infectious diseases) to reinforce messages from training, education sessions or guidelines [67]	7	3	2	12	5

^**a**^Definitions from [41].

Eight of the potential nine intervention types were used at least once across all interventions. ‘Coercion’ was the only intervention type not used. ‘Enablement’ and ‘environmental restructuring’ were the most commonly used intervention types (*n* = 19 and *n* = 17 respectively), followed by ‘education’ (*n* = 16), ‘persuasion’ (*n* = 12) and ‘training’ (*n* = 9), which were all used in more than half of included interventions. ‘Restriction’ and ‘incentivisation’ were each used only once.

Eighteen of the 93 BCTs in the BCT Taxonomy were identified at least once in the interventions. For interventions in which education was mentioned in general terms, the BCT Taxonomy cluster heading ‘Shaping Knowledge’ was used to describe it. ‘Instruction on how to perform the behaviour’ was delivered in all but one intervention (*n* = 18). ‘Adding objects to the environment’ (*n* = 13) and ‘credible source’ (n = 12) were also commonly delivered. The least frequently used BCTs were ‘self-monitoring outcome(s) of behaviour’, ‘material incentive (behaviour)’ and ‘review behaviour goal’, each used in only one intervention.

### How effective were interventions?

Seven interventions were categorised as very promising, eight quite promising and four not promising; suggesting that 79% of interventions had at least some effect on improving antimicrobial stewardship behaviours (see Table 1 for study characteristic and intervention effects).

### Which intervention types and BCTs are associated with intervention effectiveness?

The small numbers of interventions identified in this context prevented the use of inferential statistics to explore associations between intervention promise and the number of intervention types and BCTs used within an intervention.

**Fig. 3:**
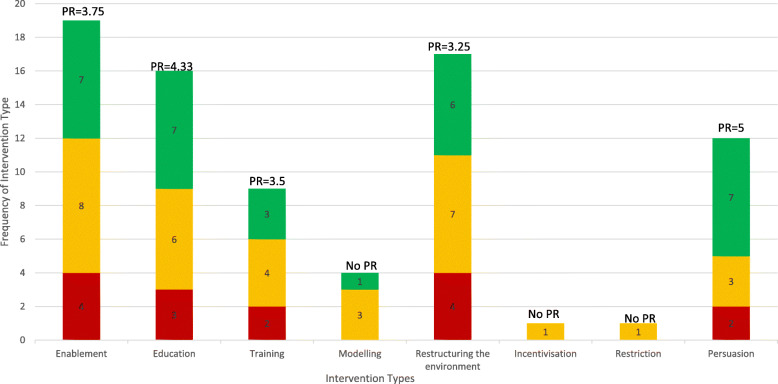
Frequency of each intervention type’s association with very, quite and not promising interventions

Figure 3 and Table 3 display intervention types by promise ratio and provide definitions and examples of each intervention type. The most promising intervention types included ‘persuasion’ (*n* = 12; promise ratio (PR) = 5.0), ‘enablement’ (*n* = 16; PR = 4.33) and ‘education’ (*n* = 19; PR = 3.75). The intervention type with the lowest promise ratio was ‘environmental restructuring’ (*n* = 17; PR = 3.25). Two intervention types (‘incentivisation’ and ‘restriction’) were only used in one intervention each and so promise ratios could not be calculated. Both these intervention types were used in interventions categorised as quite promising.

**Fig. 4:**
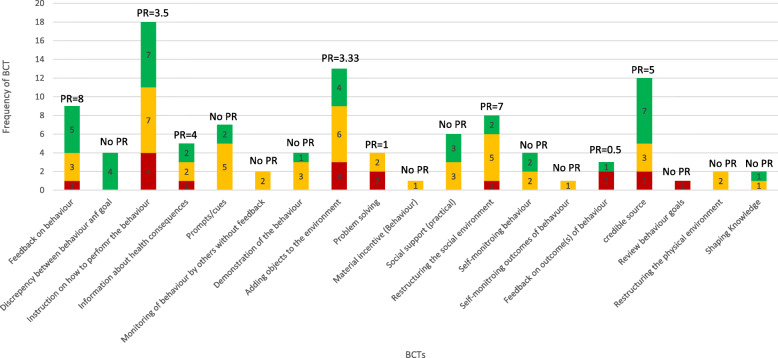
Frequency of each behaviour change technique’s association with very, quite and not promising interventions

Figure 4 and Table 4 display BCTs by promise ratio and provide definitions and examples of each BCT. The most promising BCTs were ‘feedback on behaviour’ (e.g. providing feedback on clinician prescription rates) (*n* = 9; PR = 8.0) and ‘restructuring the social environment’ (e.g. assigning a new role to a staff member to support stewardship) (*n* = 8; PR = 7.0). The least promising BCTs included ‘problem solving’ (e.g. troubleshooting difficulties faced when trying to empirically prescribe) (*n* = 4; PR = 1) and ‘feedback on outcomes of behaviour’ (e.g. providing feedback on local antimicrobial resistance pattern) (*n* = 3; PR = 0.5). Three BCTs (‘self-monitoring outcome(s) of behaviour’, ‘review behaviour goal’ and ‘material incentive (behaviour)’) were only used in one intervention each and so promise ratios could not be calculated. ‘Review behaviour goal’ was used in an intervention categorised as not promising, and ‘self-monitoring outcome(s) of behaviour’ and ‘material incentive (behaviour)’ were used in interventions categorised as quite promising.

**Table 4:** BCTs - definitions, examples, frequency and association with intervention outcomes

BCT	Definition^**a**^	Example	No. used in very promising intervention	No. used in quite promising interventions	No. used in not promising interventions	Total no. of times used across all interventions	Promise ratio
**Feedback on behaviour**	*Monitor and provide informative or evaluative feedback on performance of the behaviour (*e.g. *form, frequency, duration, intensity)*	“presented local pre-test prescribing in comparison with overall pre-test data and qualitative data on factors influencing antibiotic prescribing behaviour.” [59]	5	3	1	9	8
**Discrepancy between behaviour and goal**	*Draw attention to discrepancies between a person’s current behaviour (in terms of the form, frequency, duration, or intensity of that behaviour) and the person’s previously set outcome goals, behavioural goals or action plans (goes beyond self-monitoring of behaviour)*	“individualized direct feedback regarding specific instances when inappropriate urine cultures were sent and when ASB was treated” [71]	4	0	0	4	–
**Instruction on how to perform the behaviour**	*Advise or agree on how to perform the behaviour (includes ‘Skills training’)*	““a 60-min presentation summarizing treatment recommendations” [69]	7	7	4	18	3.5
**Information about health consequences**	*Provide information (*e.g. *written, verbal, visual) about health consequences of performing the behaviour*	“education was provided regarding the potential adverse effects of unnecessary antibiotic use, including promotion of antibiotic resistance,” [71]	2	2	1	5	4
**Prompts/cues**	*Introduce or define environmental or social stimulus with the purpose of prompting or cueing the behaviour. The prompt or cue would normally occur at the time or place of performance*	“Posters and other promotional material such as bookmarks were also distributed” [65]	2	5	0	7	–
**Monitoring of behaviour by others without feedback**	*Observe or record behaviour with the person’s knowledge as part of a behaviour change strategy*	“pharmacists did not interfere with antibiotic prescribing, but collected data on antibiotics prescribed, duration of therapy, laboratory tests, signs and symptoms of infection, and culture and sensitivity results” [67]	0	2	0	2	–
**Demonstration of the behaviour**	*Provide an observable sample of the performance of the behaviour, directly in person or indirectly* e.g. via *film, pictures, for the person to aspire to or imitate (includes ‘Modelling’).*	“stimulated interactions between the participants” [60]	1	3	0	4	–
**Adding objects to the environment**	*Add objects to the environment in order to facilitate performance of the behaviour*	“the introduction of the RAMP antimicrobial stewardship tool” [65]	4	6	3	13	3.33
**Problem solving**	*Analyse, or prompt the person to analyse, factors influencing the behaviour and generate or select strategies that include overcoming barriers and/or increasing facilitators*	“prompted to … identify barriers to implementation, to develop strategies for addressing those barriers, and to discuss and clarify their role in implementation” [56]	0	2	2	4	1
**Material incentive (Behaviour)**	*Inform that money, vouchers or other valued objects will be delivered if and only if there has been effort and/or progress in performing the behaviour (includes ‘Positive reinforcement’)*	“the intervention facilities were paid an additional $1000 each year during the 2 intervention years to incentivize guideline compliance” [53]	0	1	0	1	–
**Social support (practical)**	*Advise on, arrange, or provide practical help (*e.g. *from friends, relatives, colleagues, ‘buddies’ or staff) for performance of the behaviour*	“the providers were given a telephone number for both the infectious diseases physician on call and the antibiotic stewardship pharmacist. They were informed that this number could be called 24 h a day 7 days a week for any infectious disease related questions” [69]	3	3	0	6	–
**Restructuring the social environment**	*Change, or advise to change the social environment in order to facilitate performance of the wanted behaviour or create barriers to the unwanted behaviour (other than prompts/cues, rewards and punishments)*	“The homes identified a study liaison nurse who was the facility’s change agent for the study” [53]	2	5	1	8	7
**Self-monitoring behaviour**	*Establish a method for the person to monitor and record their behaviour(s) as part of a behaviour change strategy*	“We asked the nurses to complete a one page log of presenting symptoms and signs for every resident in whom urinary tract infection was suspected, as a reminder to use the algorithms.” [23]	2	2	0	4	–
**Self-monitoring outcomes of behaviour**	*Establish a method for the person to monitor and record the outcome(s) of their behavior as part of a behavior change strategy*	“results of specimens/swabs or ‘not available yet’ or ‘none taken’ recorded; outcome of antibiotic treatment documented” [65]	0	1	0	1	–
**Feedback on outcome(s) of behaviour**	*Monitor and provide feedback on the outcome of performance of the behaviour*	“use of antibiograms” [69]	1	0	2	3	0.5
**Credible source**	*Present verbal or visual communication from a credible source in favour of or against the behaviour*	“The LID consultation service consisted of an infectious disease physician and nurse practitioner” [57]	7	3	2	12	5
**Review behaviour goals**	*Review behaviour goal(s) jointly with the person and consider modifying goal(s) or behaviour change strategy in light of achievement. This may lead to re-setting the same goal, a small change in that goal or setting a new goal instead of (or in addition to) the first, or no change*	“the identification of opportunities for improved practice (i.e. planning action)” [59]	0	0	1	1	–
**Restructuring the physical environment**	*Change, or advise to change the physical environment in order to facilitate performance of) the wanted behaviour or create barriers to the unwanted behaviour (other than prompts/cues, rewards and punishments*	“Change to default stop dates for some antibiotics - simplified access to guidelines on computers” [62]	0	2	0	2	–
**Shaping Knowledge**	*This is the cluster heading for BCTs that serve an educational purpose in the taxonomy*	“Residents, their family members, and other NH staff received an informational brochure related to antibiotic prescribing and the QI program, and many attended family night gatherings or a resident council meeting or health fair where this information was presented” [64]	1	1	0	2	–

*BCT* Behavioural Change Technique, *No.* Number

^a^Definitions from [42].

### Quality appraisal

Of the five RCTs included in this review [23, 55, 56, 60, 65], only two were judged as low risk of bias [56, 65]. Incomplete outcome data was the primary reason for an increased risk of bias. Fifteen studies, employing non-randomised study designs, had varying levels of bias. Eight studies were judged to be at a serious risk of bias [53, 58, 59, 62, 63, 66–68], two a critical risk of bias [54, 57], three a moderate risk of bias [61, 64, 71] and two a low risk of bias [69, 70]. Studies with a critical risk of bias had confounders at baseline (e.g. adherence to guidelines was significantly different in control vs. intervention group at baseline) [54] and selection biases (such as selecting intervention sites based on staff recommendations) [57]. Similarly, baseline confounders were the most prominent reason for serious risk of bias [59, 63, 66, 67]. See Supplement 3 for full quality appraisal assessments.

Sensitivity analysis showed no statically significant relationship between study quality and intervention promise (Fisher’s Exact Test *p* = 0.533), although 86% of interventions in studies with low/moderate risk of bias were categorised as promising (three very promising, three quite promising and one not promising) compared to 73% of interventions in studies with a higher risk of bias (two very promising, seven quite promising, three not promising). The number of BCTs employed in interventions were similar for studies with low or moderate risk of bias and studies with higher risk of bias (mean number of BCTs = 5.6, range 1–8 Vs. mean number of BCTs = 6.5, range 4–8 respectively).

## Discussion

This review aimed to specify the active ingredients in antimicrobial stewardship interventions in LTCFs, in particular the BCTs associated with improved outcomes. A range of intervention strategies were used to improve antibiotic use in LTCFs. ‘Persuasion’, ‘enablement’, ‘feedback on behaviour’ and/or ‘restructuring of the social environment’ showed most promise. In contrast, ‘feedback on outcome(s) of behaviour’ and/or ‘problem solving’ showed least promise.

Enablement has been identified previously as an important component within effective antimicrobial stewardship interventions. Our findings echo that of Davey and colleagues (2017) [24] who found that enablement, including strategies with restrictive components (e.g. removal of certain antibiotics from clinical areas), was linked to intervention effectiveness.

One particular type of enablement intervention is audit and feedback, defined as a “summary of the clinical performance of healthcare provider(s) over a specified period of time” [72, 73]. Interventions including BCTs associated with audit and feedback (e.g. feedback on behaviour) have shown more promise. For example, a systematic review exploring effectiveness of antimicrobial stewardship interventions within the emergency department found that studies which employed audit and feedback strategies achieved statistically significant increases in appropriateness of antimicrobial prescribing and adherence to guidelines, as well as decreases in antimicrobial use [74]. Similarly, primary research exploring audit and feedback interventions in settings such as a tertiary care hospital [75] and an intensive care unit [76] found reduced use of antibiotics such as fluoroquinolones by 2.3 defined daily doses/1000 patient-days [95% CI − 3.97 to − 0.63] and significant reductions of antimicrobial use by 24.3% (87.3 defined daily doses/100 beds vs. 66.1 defined daily doses/100 beds; *P* < 0.001) respectively.

Less evidence is available to support the finding from this review that ‘restructuring the social environment’ (such as assigning staff to new roles e.g. antimicrobial stewardship champion) was associated with the most promising interventions. In part, there may be fewer studies using this BCT as it is likely to require more expensive or less acceptable changes within a healthcare environment that may not always be feasible in all contexts (for example in a financial and time pressured UK National Health Service). However, this finding is in line with studies of influences on antimicrobial stewardship behaviours, which have found social and cultural factors are key drivers of antimicrobial stewardship [77]. As such, this may explain why ‘restructuring the social environment’ was identified as promising in this review. More research is warranted to further explore the effect of this BCT upon antimicrobial stewardship.

### Quality of included studies

There are recognised challenges to conducting research in LTCFs, particularly RCTs and other clinical trials [78–80]. Over half the studies were judged to be of poor quality, largely because of the inclusion of small sample sizes and therefore underpowered analyses (e.g. *n* = 29) [67]. This meant that it is not possible to be confident about the validity of the findings of these studies.

### Strengths, limitations and implications

To our knowledge this is the first study that applied behavioural science frameworks to synthesise evidence and explore heterogeneity of antimicrobial stewardship interventions in LTCFs. Application of behavioural science frameworks has enabled systematic and transparent specification of intervention content associated with more or less promising interventions, allowing for better consideration of reasons for varying effectiveness of current antimicrobial stewardship interventions delivered in LTCFs. Despite not being able to draw firm conclusions about associations with effectiveness, it provides a good foundation on which to develop new interventions targeting antimicrobial stewardship in this context by highlighting gaps in existing interventions and missed opportunities for intervention design. It can also inform efforts to refine and enhance existing interventions. These are the objectives of a programme of research, funded by the Economic and Social Research Council called ‘Preserving Antibiotics through Safe Stewardship’ which will integrate the findings from this review with epidemiological analyses of prescribing patterns in LTCFs, as well as ethnographic and qualitative investigations of drivers of antimicrobial stewardship in LTFCs, to design theory- and evidence-based antimicrobial stewardship programmes in this context [81].

There were limitations to the evidence synthesised in this review that affect interpretation of findings. Firstly, given the limited research investigating antimicrobial stewardship interventions in LTCFs, a range of study designs were included, which meant variable quality and substantial heterogeneity in reported outcomes. In addition, a range of metrics were used to evaluate the effects of the interventions which precluded meta-analysis, thus affecting the ability to draw firm conclusion about associations between BCTs and effectiveness. As meta-regression was not feasible, we were not able to isolate the specific contribution of each intervention type or BCT to intervention effectiveness, nor could we examine the contribution of combinations of intervention types and BCTs to effectiveness. Instead, we could conclude whether or not interventions containing the intervention type or BCT were more or less promising, based on promise ratio calculations. This method did provide a meaningful descriptive measure upon which to summarise findings, but applies equal weighting to each study and thus does not account for factors such as variation in study sample size, study quality and effect sizes. Future research should consider testing antimicrobial stewardship interventions with high quality, robust study designs. Also, applying consistent and agreed upon metrics to appropriately evaluate interventions (i.e. developing a core outcome set) should be considered in order to allow for more robust analysis of outcomes in systematic reviews (such as meta-analysis and meta-regression).

Secondly, the majority of interventions were from the US. Therefore, findings may not be readily generalisable to other healthcare contexts (such as those within Europe or developing countries). Further, healthcare assistants (or nursing assistants) form a large number of healthcare workers within LTCFs. From the data collected in this review, the interventions delivered were not generally tailored to this group. Nearly all the interventions focused on prescribing behaviours (e.g. adherence to guidelines), a behaviour which is not relevant to these non-prescribing roles. Interventions to improve antimicrobial stewardship in LTCFs might benefit from targeting other stewardship behaviours of non-prescribing healthcare professional roles (e.g. care assistant, nurses, pharmacists, managers), as they play a vital role in enacting the key behaviours contributing to antimicrobial stewardship in LTCFs. These include helping to identify and diagnose suspected infections (e.g. collecting and sending urine cultures for testing for suspected urinary tract infections), acting as ‘gate keepers’, communicating and escalating suspected infections to doctors and managing infections without antibiotics [82].

Only 18 of a potential 93 BCTs were identified in this review. However, there is no evidence to suggest that interventions incorporating a greater number of BCTs are more effective than others [51]; rather, it is important to match the choice of BCT to the drivers of the behaviour of interest [41]. It is likely that some of the 93 BCTs may not be appropriate in this context (e.g. punishment). The findings from this and other antimicrobial stewardship reviews in different settings [24] indicate that the same types of interventions and BCTs are being used repeatedly in antimicrobial stewardship interventions (e.g. ‘feedback on behaviour’, ‘instruction on how to perform behaviour’, ‘information about health consequences’). Whilst this could be because these BCTs show promise and acceptability in this context, it could also signal potential research waste and stagnant science [83]. Behaviour change theories and models such as the Capability, Opportunity and Motivation model of Behaviour (COM-B) [41], which sits in the centre of the BCW, summarise factors influencing behaviour change. COM-B argues that in order for a desired behaviour to occur, the individual(s) whose behaviour you are trying to change must have the Capability (knowledge and skills), Opportunity (physical and social), and Motivation (reflective and automatic). It is important to ensure interventions include a range of strategies to address barriers and enablers within Capability, Opportunity, Motivation. There are tools which map the BCT taxonomy and BCW to COM-B [41, 84, 85]. We consulted these to map the BCTs and intervention types identified from studies included in this review against influences on behaviour (Supplement 4, Table 1). We identified that capability (knowledge and skills) and reflective motivation (e.g. perceived consequences, priority, goals) were targeted more frequently by component BCTs included in existing studies, compared to automatic motivation (e.g. emotions, habits), physical capability (e.g. resources, time, layout of the working environment, cues, prompts) and social opportunity (e.g. social support, norms, pressure, roles, identity) which were less frequently targeted by the component BCTs identified from included interventions. Use of a narrow range of BCTs (in terms of number and type) may therefore mean that only a narrow range of drivers of behaviour are being targeted by interventions and thus opportunities are being missed to target a broader range of drivers of behaviour. There is scope to explore a broader range of interventions to improve antimicrobial stewardship practice, particularly interventions that target social and motivational drivers of behaviour. There is a need for more intervention development and evaluation in antimicrobial stewardship that draws on behavioural and social science [40].

A limitation of the analysis approach for this review was that we explored the association of individual intervention types and BCTs with outcomes, rather than groups of BCTs or intervention types co-delivered in the same intervention package. Behaviour change interventions are typically complex, often containing multiple interacting components [39]. The effect of a BCT may be dependent upon whether or not it is delivered alongside another BCT. This relationship can be positive (whereby co-delivering BCTs can enhance effects of another), or negative (where delivering other BCTs can negatively impact or dilute the effectiveness of an individual BCT). For example, BCTs such as ‘goal setting’ and ‘feedback on behaviour’ would likely be complimentary. It was not possible to explore this in the present review due to the limited number of studies. However, future reviews would benefit from drawing on behaviour change theories to identify theory-based hypotheses or groupings of BCTs, and exploring whether interventions containing a greater number of theory-based BCTs are associated with effectiveness [86].

We identified poor or inexplicit reporting of interventions in published reports (e.g. limited description of specific aspects of an education session, lack of clarity as to whether behaviours (i.e. prescribing) or outcomes of behaviours (i.e. antimicrobial resistance patterns) were being assessed). This hampered the ability to extract and code the intervention components. This limits what could be learnt, interpreted and replicated from the findings, which in turn affects the ability to scale up promising interventions. This limitation of existing studies highlights improvements that need to be made for reporting of interventions, beyond the context of antimicrobial stewardship in LTCFs. Poor specification of behaviours is a problem because antimicrobial stewardship involves multiple behaviours, and so clarity is required to understand what is being targeted by the intervention, and subsequently what is being measured for change. Only by providing this clarity will replication and interpretation of antimicrobial stewardship interventions be enhanced. In addition to the frameworks used in this review, use of reporting checklists, such as the Template for Intervention Description and Replication (TIDieR) checklist [87], should be considered in future work.

The BCW also includes a layer of 7 Policy strategies (e.g. legislation and fiscal measures). Coding these was beyond the scope of this review. These categories are often used to support implementation of an intervention. For example, legislation can support implementation of the intervention type restriction (e.g. using rules to reduce the opportunity to smoke) by prohibiting the use of cigarettes in specific areas (like hospital grounds). The aim of this review was to generate generalisable lessons about what types of intervention strategies are often used and show promise of effectiveness in this context, as to inform design of future antimicrobial stewardship programmes or refinement of existing programmes. Whilst policy and regulation play a vital role and future research exploring the use of policy in interventions is warranted, modifying practice at a policy/regulatory level is not always feasible or accessible for many intervention designers. As such, this review focused on extracting and synthesising data at the level of intervention components and techniques that are potentially more accessible and/or feasible to incorporate for a wider range of intervention designers.

Lastly, interventions lacked rationale for the choice of strategy, or theoretical basis, with only three studies reporting any form of theory or framework to support intervention design. It has been argued that that explicit use of theory could improve intervention design, facilitate the evaluation of intervention effectiveness, support identification of contextual factors necessary for intervention success and enhance learning [88]. Relevant evidence has identified the benefit of selecting intervention content that align with theory [89]. For example, a meta-analysis showed that selection of strategies that aligned with Control Theory (such as ‘self-monitoring behaviour’, ‘feedback on behaviour’ and ‘goal setting’) were twice as effective as other interventions [90]. This suggests the need for action for better intervention design and explicit reporting of design rationale and intervention content in order to support future delivery and scalability of interventions targeting antimicrobial stewardship.

## Conclusion

This review provides a first step towards identifying the ‘active ingredients’ in interventions to improve antimicrobial stewardship in LTCFs. Interventions involving ‘enablement’, ‘environmental restructuring’ and delivery of audit and feedback strategies are likely to enhance antimicrobial stewardship. These findings can inform the design of future, or refinement of existing, interventions in this context. Higher quality trials of antimicrobial stewardship interventions in LTCFs, with systematic, transparent and consistent specification of intervention content are needed to strengthen the evidence in this area.

## Supplementary information

**Additional file 1.**

## Data Availability

Data generated and/or analysed for this study are included in this published article.

## Abbreviations

BCT: Behaviour Change Technique
BCW: Behaviour Change Wheel
CI: Confidence Interval
COM-B: Capability, Opportunity and Motivation Model of Behaviour
EPR: Estimated prevalence rate
cRCT: Cluster Randomised Controlled Trial
IRR: Incidence rate ratios
LTCF: Long-term care facility
LRTI: Lower Respiratory Tract Infection
NHAP: Nursing Home Acquitted Pneumonia
NR: Not reported
PR: Promise Ratio
PRISMA: Preferred Reporting Items for Systematic Reviews and Meta-Analyses
OR: Odds Ratio
RCT: Randomised Controlled Trial
ROBINS-I: Risk Of Bias In Non-randomised Studies - of Interventions
RTI: Respiratory Tract Infection
SSTI: Skin and Soft Tissue Infection
TACTA: Target, Action, Context, Timeframe, Actor
TIDieR: Template for Intervention Description and Replication
UK: United Kingdom
US: United States
UTI: Urinary Tract Infection
