# Supplementary material for: Interventions to improve appropriate antibiotic prescribing in long-term care facilities: a systematic review
Source: BMC Geriatr. 2020 Jul 9;20:237. doi: 10.1186/s12877-020-01564-1 (PMC7350746; doi:10.1186/s12877-020-01564-1)
Supplement: Supplementary file 1 — Additional file 1. [file 12877_2020_1564_MOESM1_ESM.docx]

**Supplementary Material**

**Supplement 1: Search Strategy**

**Ovid MEDLINE(R) and Epub Ahead of Print, In-Process & Other Non-Indexed Citations, and Daily**1946 to July 16, 2018

1. exp Nursing Homes/
2. exp Long-Term Care/
3. exp Residential Facilities/
4. Hospitals, Veterans/
5. 1 or 2 or 3 or 4
6. (((Long?term care or Residential care or healthcare or Assisted living) and (facilit$ or center? or centre? or unit?)) or (Nursing adj3 home?) or (veterans and (hospital? or facilit$ or center? or centre? or unit?))).mp. [mp=title, abstract, heading word, table of contents, key concepts, original title, tests & measures]
7. 5 or 6
8. Anti-Bacterial agents/
9. Anti-infective Agents/
10. Antibiotic Prophylaxis/
11. Antimicrobial stewardship/
12. 8 or 9 or 10 or 11
13. (((Antibiotic? or Anti?biotic? or Antimicrobi$ or Anti?microbi$) and (therap$ or prophylaxis or stewardship)) or Anti?infective agent?).mp. [mp=title, abstract, heading word, table of contents, key concepts, original title, tests & measures]
14. 12 or 13
15. randomized controlled trial.pt.
16. controlled clinical trial.pt.
17. pragmatic clinical trial.pt.
18. multicenter study.pt.
19. interrupted time series analysis/
20. controlled before-after studies/
21. (randomis$ or randomiz$ or randomly).ti,ab.
22. groups.ab.
23. (trial or multicenter or multi center or multicentre or multi centre).ti.
24. (intervention? or effect? or impact? or controlled or control group? or (before adj5 after) or (pre adj5 post) or quasiexperiment$ or quasi experiment$ or evaluat$ or time series or time point? or repeated measur$ or ((pretest or "pre test") and (posttest or "post test"))).ti,ab.
25. review.pt.
26. meta analysis.pt.
27. (systematic review or literature review).ti
28. 15 or 16 or 17 or 18 or 19 or 20 or 21 or 22 or 23 or 24 or 25 or 26 or 27
29. 7 and 14 and 28
30. Limit 29 to english language

**Embase Classic+Embase**1947 to 2018 July 16

1. exp Nursing Homes/
2. exp Long-Term Care/
3. exp Residential Facilities/
4. Hospitals, Veterans/
5. 1 or 2 or 3 or 4
6. (((Long?term care or Residential care or healthcare or Assisted living) and (facilit$ or center? or centre? or unit?)) or (Nursing adj3 home?) or (veterans and (hospital? or facilit$ or center? or centre? or unit?))).ti,ab.
7. 5 or 6
8. Anti-Bacterial agents/
9. Anti-infective Agents/
10. Antibiotic Prophylaxis/
11. Antimicrobial stewardship/
12. 8 or 9 or 10 or 11
13. (((Antibiotic? or Anti?biotic? or Antimicrobi$ or Anti?microbi$) and (therap$ or prophylaxis or stewardship)) or Anti?infective agent?).ti,ab.
14. 12 or 13
15. interrupted time series analysis/
16. controlled before-after studies/
17. (randomis$ or randomiz$ or randomly).ti,ab.
18. groups.ab.
19. (trial or multicenter or multi center or multicentre or multi centre).ti.
20. (intervention? or effect? or impact? or controlled or control group? or (before adj5 after) or (pre adj5 post) or quasiexperiment$ or quasi experiment$ or evaluat$ or time series or time point? or repeated measur$ or ((pretest or "pre test") and (posttest or "post test"))).ti,ab.
21. review.pt.
22. (systematic review or literature review).ti
23. 15 or 16 or 17 or 18 or 19 or 20 or 21 or 22
24. 7 and 14 and 23
25. Limit 24 to english language
26. Limit 25 to embase

**PsycINFO**1806 to July Week 2 2018

1. exp Nursing Homes/
2. exp Long-Term Care/
3. exp Residential Care Institutions/
4. 1 or 2 or 3
5. (((Long?term care or Residential care or healthcare or Assisted living) and (facilit$ or center? or centre? or unit?)) or (Nursing adj3 home?) or (veterans and (hospital? or facilit$ or center? or centre? or unit?))).mp. [mp=title, abstract, heading word, table of contents, key concepts, original title, tests & measures]
6. 4 or 5
7. Exp Antibiotics/
8. (((Antibiotic? or Anti?biotic? or Antimicrobi$ or Anti?microbi$) and (therap$ or prophylaxis or stewardship)) or Anti?infective agent?).mp. [mp=title, abstract, heading word, table of contents, key concepts, original title, tests & measures]
9. 7 or 8
10. (randomis$ or randomiz$ or randomly).ti,ab.
11. groups.ab.
12. (trial or multicenter or multi center or multicentre or multi centre).ti.
13. (intervention? or effect? or impact? or controlled or control group? or (before adj5 after) or (pre adj5 post) or quasiexperiment$ or quasi experiment$ or evaluat$ or time series or time point? or repeated measur$ or ((pretest or "pre test") and (posttest or "post test"))).ti,ab.
14. (systematic review or literature review).ti.
15. 10 or 11 or 12 or 13 or 14
16. 6 and 9 and 15
17. Limit 16 to english language

**Cochrane Library** Inception-present 17-7-2018

1. exp Nursing Homes/
2. exp Long-Term Care/
3. exp Residential Facilities/
4. exp Hospitals, Veterans/
5. 1 or 2 or 3 or 4
6. (((Long?term care or Residential care or healthcare or Assisted living) and (facilit* or center? or centre? or unit?)) or (Nursing NEXT home?) or (veterans and (hospital? or facilit* or center? or centre? or unit?)))
7. 5 or 6
8. Anti-Bacterial agents/
9. exp Anti-infective Agents/
10. exp Antibiotic Prophylaxis/
11. exp Antimicrobial stewardship/
12. 8 or 9 or 10 or 11
13. (((Antibiotic? or Anti?biotic? or Antimicrobi* or Anti?microbi*) and (therap* or prophylaxis or stewardship) or Anti?infective agent?))
14. 12 or 13
15. randomized controlled trial:pt
16. controlled clinical trial:pt
17. pragmatic clinical trial:pt
18. multicenter study.pt.
19. Non-randomized controlled trials as topic/
20. interrupted time series analysis/
21. controlled before-after studies/
22. (randomis* or randomiz* or randomly):ti,ab,kw
23. Groups:ab
24. (trial or multicenter or multi center or multicentre or multi centre):ti.
25. (intervention? or effect? or impact? or controlled or control group? or (before NEXT/5 after) or (pre NEXT/5 post) or quasiexperiment* or quasi experiment* or evaluat* or time series or time point? or repeated measur* or ((pretest or “pre test”) and (posttest or “post test”))):ti,ab,kw
26. Review:pt.
27. meta analysis:pt
28. (systematic review or literature review):ti
29. 15 or 16 or 17 or 18 or 19 or 20 or 21 or 22 or 23 or 24 or 25 or 26 or 27 or 28
30. 7 and 14 and 29

**Web of Science core collection** Timespan all years – 17-7-2018

1. TS=(((“Long-term care” or “Residential care” or healthcare or “Assisted living”) and (facilit* or center* or centre* or unit*)) or “Nursing home*” or (veterans and (hospital* or facilit* or center* or centre* or unit*)))
2. TS=((((Antibiotic* or Anti-biotic* or Antimicrobi* or Anti-microbi*) NEAR (therap* OR prophylaxis or stewardship)) or “Anti-infective agent*”))
3. TS=(randomis* or randomiz* or randomly)
4. TS=(groups)
5. TI=(trial or multicenter or “multi center” or multicentre or “multi centre”)
6. TS=((intervention? or effect? or impact? or controlled or “control group?” or (before NEAR/5 after) or (pre NEAR/5 post) or ((pretest or “pre test”) and (posttest or “post test”)) or quasiexperiment* or “quasi experiment*” or evaluat* or “time series” or “time point?” or “repeated measur*”))
7. TI=(“systematic review” or “literature review”)
8. 3 or 4 or 5 or 6 or 7
9. 1 AND 2 AND 8 *Indexes=SCI-EXPANDED, SSCI, A&HCI, CPCI-S, CPCI-SSH, BKCI-S, BKCI-SSH, ESCI, CCR-EXPANDED, IC Timespan=All years*
10. Restricted to English Language

**Supplement 2. Intervention functions, BCTs**

Table 1. Table displaying the total numbers of intervention types and BCTs identified in each study included in the review.

| Author | | Monette 2007 | Naughton 2001 | Jump 2013 | McMaughan 2016 | van Buul 2015 | Petterson 2011 | Schwartz 2007 | Loeb 2005 | Kassett 2016 | Doemberg 2017 | Zimmerman 2014 | Fleet 2014 | Furuno 2014 | Gugkaeva 2012 | Hutt 2006 | Linnebur 2011 | Rehme 2016 | Smith 2016 | Hutt 2011 | Zabarsky 2006 |
| --- | --- | --- | --- | --- | --- | --- | --- | --- | --- | --- | --- | --- | --- | --- | --- | --- | --- | --- | --- | --- | --- |
| Intervention functions | Enablement | ✓ | ✓ | ✓ | ✓ | ✓ | ✓ | ✓ | ✓ | ✓ | ✓ | ✓ | ✓ | ✓ | ✓ | ✓ | ✓ | ✓ | ✓ | ✓ | ✓ |
|  | Education | ✓ | ✓ | ✓ | - | ✓ | ✓ | ✓ | - | ✓ | ✓ | ✓ | ✓ | ✓ | ✓ | ✓ | ✓ | ✓ | - | ✓ | ✓ |
|  | Training | - | ✓ | - | ✓ | - | - | ✓ | ✓ | - | ✓ | ✓ | ✓ | - | - | ✓ | ✓ | - | - | ✓ | - |
|  | Modelling | - | - | ✓ | - | - | ✓ | - | ✓ | - | - | - | - | - | - | ✓ | - | - | - | - | - |
|  | Environmental Restructuring | - | ✓ | ✓ | ✓ | ✓ | - | ✓ | ✓ | ✓ | ✓ | ✓ | ✓ | ✓ | ✓ | ✓ | ✓ | ✓ | ✓ | ✓ | ✓ |
|  | Incentivisation | - | - | - | - | - | - | - | - | - | - | - | - | - | - | - | ✓ | - | - | ✓ | - |
|  | Restriction | - | - | - | - | - | - | - | - | - | - | - | - | - | - | - | ✓ | - | - | ✓ | - |
|  | Persuasion | ✓ | - | ✓ | ✓ | - | - | ✓ | - | - | ✓ | ✓ | ✓ | ✓ | ✓ | ✓ | - | ✓ | - | - | ✓ |
|  | **Total** | **3** | **4** | **5** | **4** | **3** | **3** | **5** | **4** | **3** | **5** | **5** | **5** | **4** | **4** | **6** | **6** | **4** | **2** | **6** | **4** |
| BCTs | Feedback on Behaviour | ✓ | - | - | - | ✓ | ✓ | ✓ | - | ✓ | ✓ | ✓ | - | - | - | - | ✓ | - | - | ✓ | ✓ |
|  | Discrepancy between behaviour and goal | ✓ | - | - | - | - | - | ✓ | - | - | - | ✓ | - | - | - | - | - | - | - | - | ✓ |
|  | Instruction on how to perform the behaviour | ✓ | ✓ | ✓ | ✓ | ✓ | ✓ | ✓ | ✓ | ✓ | ✓ | ✓ | ✓ | ✓ | ✓ | ✓ | ✓ | ✓ | - | ✓ | ✓ |
|  | Information about health consequences | - | - | - | - | ✓ | ✓ | - |  | - | - | - | ✓ | - | - | - | - | ✓ | - | - | ✓ |
|  | Prompts/cues | - | - | - | - | - | - | - | ✓ | ✓ | - | ✓ | ✓ | - | - | ✓ | ✓ | - | - | ✓ | ✓ |
|  | Monitoring of behaviour by others without feedback | - | - | - | - | - | - | - | ✓ | - | - | - | - | - | ✓ | - | - | - | - | - | - |
|  | Demonstration of the behaviour | - | - | ✓ | - | - | ✓ | - | ✓ | - | - | - | - | - | - | ✓ | - | - | - | - | - |
|  | Adding objects to the environment | - | ✓ | - | ✓ | - | - | ✓ | ✓ | ✓ | - | ✓ | ✓ | ✓ | ✓ | ✓ | ✓ | ✓ | - | ✓ | ✓ |
|  | Problem solving | - | ✓ | - | - | ✓ | - | - | - | - | - | - | - | - | - | ✓ | ✓ | - | - | ✓ | - |
|  | Material incentive (behaviour) | - | - | - | - | - | - | - | - | - | - | - | - | - | - | - | ✓ | - | - | ✓ | - |
|  | Social support (practical) | - | - | ✓ | - | - | - | - | - | - | ✓ | - | ✓ | - | ✓ | - | ✓ | ✓ | - | ✓ | - |
|  | Restructuring the social environment | - | - | ✓ | - | ✓ | - | - | ✓ | - | ✓ | - | - | - | ✓ | ✓ | ✓ | - | ✓ | ✓ | - |
|  | Self-monitoring of behaviour | - | - | - | - | - | - | ✓ | ✓ | - | - | ✓ | ✓ | - | - | - | - | - | - | - | - |
|  | Self-monitoring outcome(s) of behaviour | - | - | - | - | - | - | - | - | - | - | - | ✓ | - | - | - | - | - | - | - | - |
|  | Feedback on outcome(s) of behaviour | - | - | - | - | ✓ | - | - | - | - | - | - | - | ✓ | - | - | - | ✓ | - | - | - |
|  | Credible Soiree | ✓ | - | ✓ | ✓ | - | - | ✓ | - | - | ✓ | ✓ | ✓ | ✓ | ✓ | ✓ | - | ✓ | - | - | ✓ |
|  | Review behaviour goals | - | - | - | - | ✓ | - | - | - | - | - | - | - | - | - | - | - | - | - | - | - |
|  | Restructuring the physical environment | - | - | - | - | - | - | - | - | ✓ | - | - | - | - | - | ✓ | - | - | - | - | - |
|  | ‘Shaping Knowledge’ | - | - | - | - | - | - | - | - | ✓ | - | ✓ | - | - | - | - | - | - | - | - | - |
|  | **Total** | **4** | **3** | **5** | **3** | **7** | **4** | **6** | **7** | **6** | **5** | **8** | **8** | **4** | **6** | **8** | **8** | **6** | **1** | **8** | **7** |

BCTs-Behaviour Change Techniques

**Supplement 3: Quality Appraisal**

**ROBINS-I Appraisal**

**Doemberg et al 2015**

Responses underlined in green are potential markers for low risk of bias, and responses in red are potential markers for a risk of bias. Where questions relate only to sign posts to other questions, no formatting is used.

|  | **Signalling questions** | **Description** | **Response options** |
| --- | --- | --- | --- |
| **Bias due to confounding** | | | |
|  | 1.1 Is there potential for confounding of the effect of intervention in this study?  **If N/PN to 1.1:** the study can be considered to be at low risk of bias due to confounding and no further signalling questions need be considered | PY  Possible confound; baseline characteristics of pre-intervention not available. | Y / PY / PN / N |
|  | **If Y/PY to 1.1**: determine whether there is a need to assess time-varying confounding: |  |  |
|  | 1.2. Was the analysis based on splitting participants’ follow up time according to intervention received?  **If N/PN**, answer questions relating to baseline confounding (1.4 to 1.6)  **If Y/PY**, go to question 1.3. | N | NA / Y / PY / PN / N / NI |
|  | 1.3. Were intervention discontinuations or switches likely to be related to factors that are prognostic for the outcome?  **If N/PN**, answer questions relating to baseline confounding (1.4 to 1.6)  **If Y/PY**, answer questions relating to both baseline and time-varying confounding (1.7 and 1.8) |  | NA / Y / PY / PN / N / NI |

|  | **Questions relating to baseline confounding only** | | |
| --- | --- | --- | --- |
|  | 1.4. Did the authors use an appropriate analysis method that controlled for all the important confounding domains? | Y comparison with baseline information comparing AM susceptibility pattern (no change); Resident characteristics were also compared. | NA / Y / PY / PN / N / NI |
|  | 1.5. **If Y/PY to 1.4**: Were confounding domains that were controlled for measured validly and reliably by the variables available in this study? | Y | NA / Y / PY / PN / N / NI |
|  | 1.6. Did the authors control for any post-intervention variables that could have been affected by the intervention? | N; adherence rates not accounted for (low rates of recommendations being followed by physicians) - furthermore, "85% of prescriptions that were not reviewed were deemed unecessary", suggesting that ASP is not as frequent/effective as desired. | NA / Y / PY / PN / N / NI |
|  | **Questions relating to baseline and time-varying confounding** | |  |
|  | 1.7. Did the authors use an appropriate analysis method that controlled for all the important confounding domains and for time-varying confounding? |  | NA / Y / PY / PN / N / NI |
|  | 1.8. **If Y/PY to 1.7**: Were confounding domains that were controlled for measured validly and reliably by the variables available in this study? |  | NA / Y / PY / PN / N / NI |
|  | **Risk of bias judgement** | Serious | Low / Moderate / Serious / Critical / NI |
|  | Optional: What is the predicted direction of bias due to confounding? |  | Favours experimental / Favours comparator / Unpredictable |

| **Bias in selection of participants into the study** | | | |
| --- | --- | --- | --- |
|  | 2.1. Was selection of participants into the study (or into the analysis) based on participant characteristics observed after the start of intervention?  **If N/PN to 2.1:** go to 2.4 | N | Y / PY / PN / N / NI |
|  | 2.2. **If Y/PY to 2.1**: Were the post-intervention variables that influenced selection likely to be associated with intervention?  2.3 **If Y/PY to 2.2**: Were the post-intervention variables that influenced selection likely to be influenced by the outcome or a cause of the outcome? |  | NA / Y / PY / PN / N / NI  NA / Y / PY / PN / N / NI |
|  | 2.4. Do start of follow-up and start of intervention coincide for most participants? | Y | Y / PY / PN / N / NI |
|  | 2.5. **If Y/PY to 2.2 and 2.3, or N/PN to 2.4**: Were adjustment techniques used that are likely to correct for the presence of selection biases? |  | NA / Y / PY / PN / N / NI |
|  | **Risk of bias judgement** | Low | Low / Moderate / Serious / Critical / NI |
|  | Optional: What is the predicted direction of bias due to selection of participants into the study? |  | Favours experimental / Favours comparator / Towards null /Away from null / Unpredictable |

| **Bias in classification of interventions** | | | |
| --- | --- | --- | --- |
|  | 3.1 Were intervention groups clearly defined? | PY; mentions of ‘clinical consensus criteria’, but no specific criteria found. | Y / PY / PN / N / NI |
|  | 3.2 Was the information used to define intervention groups recorded at the start of the intervention? | Y | Y / PY / PN / N / NI |
|  | 3.3 Could classification of intervention status have been affected by knowledge of the outcome or risk of the outcome? | Y | Y / PY / PN / N / NI |
|  | **Risk of bias judgement** | Moderate | Low / Moderate / Serious / Critical / NI |
|  | Optional: What is the predicted direction of bias due to classification of interventions? |  | Favours experimental / Favours comparator / Towards null /Away from null / Unpredictable |

| **Bias due to deviations from intended interventions** | | | |
| --- | --- | --- | --- |
|  | **If your aim for this study is to assess the effect of assignment to intervention, answer questions 4.1 and 4.2** | |  |
|  | 4.1. Were there deviations from the intended intervention beyond what would be expected in usual practice? | N | Y / PY / PN / N / NI |
|  | 4.2. **If Y/PY to 4.1**: Were these deviations from intended intervention unbalanced between groups *and* likely to have affected the outcome? |  | NA / Y / PY / PN / N / NI |
|  | **If your aim for this study is to assess the effect of starting and adhering to intervention, answer questions 4.3 to 4.6** | |  |
|  | 4.3. Were important co-interventions balanced across intervention groups? | N; possible co-intervention with the pharmacist’s weekly visit vs ASP recommendation via telephone/fax. | Y / PY / PN / N / NI |
|  | 4.4. Was the intervention implemented successfully for most participants? | Y | Y / PY / PN / N / NI |
|  | 4.5. Did study participants adhere to the assigned intervention regimen? | N; only 25% of changes were accepted. | Y / PY / PN / N / NI |
|  | 4.6. **If N/PN to 4.3, 4.4 or 4.5**: Was an appropriate analysis used to estimate the effect of starting and adhering to the intervention? | N | NA / Y / PY / PN / N / NI |
|  | **Risk of bias judgement** | Serious |  |
|  | Optional: What is the predicted direction of bias due to deviations from the intended interventions? |  |  |

| **Bias due to missing data** | | | |
| --- | --- | --- | --- |
|  | 5.1 Were outcome data available for all, or nearly all, participants? | Y | Y / PY / PN / N / NI |
|  | 5.2 Were participants excluded due to missing data on intervention status? | N | Y / PY / PN / N / NI |
|  | 5.3 Were participants excluded due to missing data on other variables needed for the analysis? | N | Y / PY / PN / N / NI |
|  | 5.4 **If PN/N to 5.1, or Y/PY to 5.2 or 5.3**: Are the proportion of participants and reasons for missing data similar across interventions? |  | NA / Y / PY / PN / N / NI |
|  | 5.5 **If PN/N to 5.1, or Y/PY to 5.2 or 5.3**: Is there evidence that results were robust to the presence of missing data? |  | NA / Y / PY / PN / N / NI |
|  | **Risk of bias judgement** | Low | Low / Moderate / Serious / Critical / NI |
|  | Optional: What is the predicted direction of bias due to missing data? |  | Favours experimental / Favours comparator / Towards null /Away from null / Unpredictable |

| **Bias in measurement of outcomes** | | | |
| --- | --- | --- | --- |
|  | 6.1 Could the outcome measure have been influenced by knowledge of the intervention received? | PY: study characteristics. It was not possible to prevent the nurses and physicians knowing whether the LTC was under intervention condition. | Y / PY / PN / N / NI |
|  | 6.2 Were outcome assessors aware of the intervention received by study participants? | PN | Y / PY / PN / N / NI |
|  | 6.3 Were the methods of outcome assessment comparable across intervention groups? | Y | Y / PY / PN / N / NI |
|  | 6.4 Were any systematic errors in measurement of the outcome related to intervention received? | N | Y / PY / PN / N / NI |
|  | **Risk of bias judgement** | Moderate | Low / Moderate / Serious / Critical / NI |
|  | Optional: What is the predicted direction of bias due to measurement of outcomes? |  | Favours experimental / Favours comparator / Towards null /Away from null / Unpredictable |

| **Bias in selection of the reported result** | | | |
| --- | --- | --- | --- |
|  | Is the reported effect estimate likely to be selected, on the basis of the results, from... |  |  |
|  | 7.1. ... multiple outcome *measurements* within the outcome domain? | N | Y / PY / PN / N / NI |
|  | 7.2 ... multiple *analyses* of the intervention-outcome relationship? | N | Y / PY / PN / N / NI |
|  | 7.3 ... different *subgroups*? | N | Y / PY / PN / N / NI |
|  | **Risk of bias judgement** | Low | Low / Moderate / Serious / Critical / NI |
|  | Optional: What is the predicted direction of bias due to selection of the reported result? |  | Favours experimental / Favours comparator / Towards null /Away from null / Unpredictable |

| **Overall bias** | | | |
| --- | --- | --- | --- |
|  | **Risk of bias judgement** | Serious | Low / Moderate / Serious / Critical / NI |
|  | Optional: What is the overall predicted direction of bias for this outcome? |  | Favours experimental / Favours comparator / Towards null /Away from null / Unpredictable |

**Furuno et al 2014**

Responses underlined in green are potential markers for low risk of bias, and responses in red are potential markers for a risk of bias. Where questions relate only to sign posts to other questions, no formatting is used.

|  | **Signalling questions** | **Description** | **Response options** |
| --- | --- | --- | --- |
| **Bias due to confounding** | | | |
|  | 1.1 Is there potential for confounding of the effect of intervention in this study?  **If N/PN to 1.1:** the study can be considered to be at low risk of bias due to confounding and no further signalling questions need be considered | Y | Y / PY / PN / N |
|  | **If Y/PY to 1.1**: determine whether there is a need to assess time-varying confounding: |  |  |
|  | 1.2. Was the analysis based on splitting participants’ follow up time according to intervention received?  **If N/PN**, answer questions relating to baseline confounding (1.4 to 1.6)  **If Y/PY**, go to question 1.3. | Y | NA / Y / PY / PN / N / NI |
|  | 1.3. Were intervention discontinuations or switches likely to be related to factors that are prognostic for the outcome?  **If N/PN**, answer questions relating to baseline confounding (1.4 to 1.6)  **If Y/PY**, answer questions relating to both baseline and time-varying confounding (1.7 and 1.8) | Y | NA / Y / PY / PN / N / NI |

|  | **Questions relating to baseline confounding only** | | |
| --- | --- | --- | --- |
|  | 1.4. Did the authors use an appropriate analysis method that controlled for all the important confounding domains? |  | NA / Y / PY / PN / N / NI |
|  | 1.5. **If Y/PY to 1.4**: Were confounding domains that were controlled for measured validly and reliably by the variables available in this study? |  | NA / Y / PY / PN / N / NI |
|  | 1.6. Did the authors control for any post-intervention variables that could have been affected by the intervention? |  | NA / Y / PY / PN / N / NI |
|  | **Questions relating to baseline and time-varying confounding** | |  |
|  | 1.7. Did the authors use an appropriate analysis method that controlled for all the important confounding domains and for time-varying confounding? | PN. there were varying levels of patient transfer to primary care which appears to be significantly different (no stats data available; 48%/79%/98% transferred to primary-associated hospital.)  Although the authors limited intake to patients in SNFs for more than 6 months to adjust, they also included patients who have been transferred to the hospital (obtaining antibiogram data from the hospital instead). It is not known if the primary-associated hospital switched AB treatment with respect to antibiogram. | NA / Y / PY / PN / N / NI |
|  | 1.8. **If Y/PY to 1.7**: Were confounding domains that were controlled for measured validly and reliably by the variables available in this study? |  | NA / Y / PY / PN / N / NI |
|  | **Risk of bias judgement** | Serious | Low / Moderate / Serious / Critical / NI |
|  | Optional: What is the predicted direction of bias due to confounding? |  | Favours experimental / Favours comparator / Unpredictable |

| **Bias in selection of participants into the study** | | | |
| --- | --- | --- | --- |
|  | 2.1. Was selection of participants into the study (or into the analysis) based on participant characteristics observed after the start of intervention?  **If N/PN to 2.1:** go to 2.4 | N | Y / PY / PN / N / NI |
|  | 2.2. **If Y/PY to 2.1**: Were the post-intervention variables that influenced selection likely to be associated with intervention?  2.3 **If Y/PY to 2.2**: Were the post-intervention variables that influenced selection likely to be influenced by the outcome or a cause of the outcome? |  | NA / Y / PY / PN / N / NI  NA / Y / PY / PN / N / NI |
|  | 2.4. Do start of follow-up and start of intervention coincide for most participants? | Y | Y / PY / PN / N / NI |
|  | 2.5. **If Y/PY to 2.2 and 2.3, or N/PN to 2.4**: Were adjustment techniques used that are likely to correct for the presence of selection biases? |  | NA / Y / PY / PN / N / NI |
|  | **Risk of bias judgement** | Low | Low / Moderate / Serious / Critical / NI |
|  | Optional: What is the predicted direction of bias due to selection of participants into the study? |  | Favours experimental / Favours comparator / Towards null /Away from null / Unpredictable |

| **Bias in classification of interventions** | | | |
| --- | --- | --- | --- |
|  | 3.1 Were intervention groups clearly defined? | Y – yes, by skilled nursing facility | Y / PY / PN / N / NI |
|  | 3.2 Was the information used to define intervention groups recorded at the start of the intervention? | Y | Y / PY / PN / N / NI |
|  | 3.3 Could classification of intervention status have been affected by knowledge of the outcome or risk of the outcome? | N | Y / PY / PN / N / NI |
|  | **Risk of bias judgement** | Low | Low / Moderate / Serious / Critical / NI |
|  | Optional: What is the predicted direction of bias due to classification of interventions? |  | Favours experimental / Favours comparator / Towards null /Away from null / Unpredictable |

| **Bias due to deviations from intended interventions** | | | |
| --- | --- | --- | --- |
|  | **If your aim for this study is to assess the effect of assignment to intervention, answer questions 4.1 and 4.2** | |  |
|  | 4.1. Were there deviations from the intended intervention beyond what would be expected in usual practice? | N | Y / PY / PN / N / NI |
|  | 4.2. **If Y/PY to 4.1**: Were these deviations from intended intervention unbalanced between groups *and* likely to have affected the outcome? |  | NA / Y / PY / PN / N / NI |
|  | **If your aim for this study is to assess the effect of starting and adhering to intervention, answer questions 4.3 to 4.6** | |  |
|  | 4.3. Were important co-interventions balanced across intervention groups? | N/A – only one intervention tested (implementation of Antibiogram) | Y / PY / PN / N / NI |
|  | 4.4. Was the intervention implemented successfully for most participants? | Y | Y / PY / PN / N / NI |
|  | 4.5. Did study participants adhere to the assigned intervention regimen? | Y | Y / PY / PN / N / NI |
|  | 4.6. **If N/PN to 4.3, 4.4 or 4.5**: Was an appropriate analysis used to estimate the effect of starting and adhering to the intervention? |  | NA / Y / PY / PN / N / NI |
|  | **Risk of bias judgement** | Low |  |
|  | Optional: What is the predicted direction of bias due to deviations from the intended interventions? |  |  |

| **Bias due to missing data** | | | |
| --- | --- | --- | --- |
|  | 5.1 Were outcome data available for all, or nearly all, participants? | PY; patients transferred to hospitals had antibiogram data sent over. | Y / PY / PN / N / NI |
|  | 5.2 Were participants excluded due to missing data on intervention status? | N | Y / PY / PN / N / NI |
|  | 5.3 Were participants excluded due to missing data on other variables needed for the analysis? | N | Y / PY / PN / N / NI |
|  | 5.4 **If PN/N to 5.1, or Y/PY to 5.2 or 5.3**: Are the proportion of participants and reasons for missing data similar across interventions? |  | NA / Y / PY / PN / N / NI |
|  | 5.5 **If PN/N to 5.1, or Y/PY to 5.2 or 5.3**: Is there evidence that results were robust to the presence of missing data? |  | NA / Y / PY / PN / N / NI |
|  | **Risk of bias judgement** | Low | Low / Moderate / Serious / Critical / NI |
|  | Optional: What is the predicted direction of bias due to missing data? |  | Favours experimental / Favours comparator / Towards null /Away from null / Unpredictable |

| **Bias in measurement of outcomes** | | | |
| --- | --- | --- | --- |
|  | 6.1 Could the outcome measure have been influenced by knowledge of the intervention received? | PN: possible that prescribed drugs are more appropriate given symptoms, although this is unlikely since symptoms across intervention/control groups are likely to be similar. Bacterial resistance unlikely to change due to assignment. | Y / PY / PN / N / NI |
|  | 6.2 Were outcome assessors aware of the intervention received by study participants? | NA. Although outcome assessors were aware, it is unlikely that bacterial resistance data can change due to having knowledge of intervention. | Y / PY / PN / N / NI |
|  | 6.3 Were the methods of outcome assessment comparable across intervention groups? | Y | Y / PY / PN / N / NI |
|  | 6.4 Were any systematic errors in measurement of the outcome related to intervention received? | N | Y / PY / PN / N / NI |
|  | **Risk of bias judgement** | Low | Low / Moderate / Serious / Critical / NI |
|  | Optional: What is the predicted direction of bias due to measurement of outcomes? |  | Favours experimental / Favours comparator / Towards null /Away from null / Unpredictable |

| **Bias in selection of the reported result** | | | |
| --- | --- | --- | --- |
|  | Is the reported effect estimate likely to be selected, on the basis of the results, from... |  |  |
|  | 7.1. ... multiple outcome *measurements* within the outcome domain? | N | Y / PY / PN / N / NI |
|  | 7.2 ... multiple *analyses* of the intervention-outcome relationship? | N | Y / PY / PN / N / NI |
|  | 7.3 ... different *subgroups*? | N | Y / PY / PN / N / NI |
|  | **Risk of bias judgement** | Low | Low / Moderate / Serious / Critical / NI |
|  | Optional: What is the predicted direction of bias due to selection of the reported result? |  | Favours experimental / Favours comparator / Towards null /Away from null / Unpredictable |

| **Overall bias** | | | |
| --- | --- | --- | --- |
|  | **Risk of bias judgement** | Serious | Low / Moderate / Serious / Critical / NI |
|  | Optional: What is the overall predicted direction of bias for this outcome? |  | Favours experimental / Favours comparator / Towards null /Away from null / Unpredictable |

**Gugkaeva et al 2012**

Responses underlined in green are potential markers for low risk of bias, and responses in red are potential markers for a risk of bias. Where questions relate only to sign posts to other questions, no formatting is used.

|  | **Signalling questions** | **Description** | **Response options** |
| --- | --- | --- | --- |
| **Bias due to confounding** | | | |
|  | 1.1 Is there potential for confounding of the effect of intervention in this study?  **If N/PN to 1.1:** the study can be considered to be at low risk of bias due to confounding and no further signalling questions need be considered | Y. | Y / PY / PN / N |
|  | **If Y/PY to 1.1**: determine whether there is a need to assess time-varying confounding: |  |  |
|  | 1.2. Was the analysis based on splitting participants’ follow up time according to intervention received?  **If N/PN**, answer questions relating to baseline confounding (1.4 to 1.6)  **If Y/PY**, go to question 1.3. | N | NA / Y / PY / PN / N / NI |
|  | 1.3. Were intervention discontinuations or switches likely to be related to factors that are prognostic for the outcome?  **If N/PN**, answer questions relating to baseline confounding (1.4 to 1.6)  **If Y/PY**, answer questions relating to both baseline and time-varying confounding (1.7 and 1.8) |  | NA / Y / PY / PN / N / NI |

|  | **Questions relating to baseline confounding only** | | |
| --- | --- | --- | --- |
|  | 1.4. Did the authors use an appropriate analysis method that controlled for all the important confounding domains? | PN. Although the author attempted to perform qualitative data instead of statistical testing as sample size was small (N = 29), sample size is too small to be controlled. | NA / Y / PY / PN / N / NI |
|  | 1.5. **If Y/PY to 1.4**: Were confounding domains that were controlled for measured validly and reliably by the variables available in this study? | PY; attempted to with qualitative data used. | NA / Y / PY / PN / N / NI |
|  | 1.6. Did the authors control for any post-intervention variables that could have been affected by the intervention? | N | NA / Y / PY / PN / N / NI |
|  | **Questions relating to baseline and time-varying confounding** | |  |
|  | 1.7. Did the authors use an appropriate analysis method that controlled for all the important confounding domains and for time-varying confounding? |  | NA / Y / PY / PN / N / NI |
|  | 1.8. **If Y/PY to 1.7**: Were confounding domains that were controlled for measured validly and reliably by the variables available in this study? |  | NA / Y / PY / PN / N / NI |
|  | **Risk of bias judgement** | Serious. Small sample size expected to interfere with obtained result. | Low / Moderate / Serious / Critical / NI |
|  | Optional: What is the predicted direction of bias due to confounding? |  | Favours experimental / Favours comparator / Unpredictable |

| **Bias in selection of participants into the study** | | | |
| --- | --- | --- | --- |
|  | 2.1. Was selection of participants into the study (or into the analysis) based on participant characteristics observed after the start of intervention?  **If N/PN to 2.1:** go to 2.4 | N | Y / PY / PN / N / NI |
|  | 2.2. **If Y/PY to 2.1**: Were the post-intervention variables that influenced selection likely to be associated with intervention?  2.3 **If Y/PY to 2.2**: Were the post-intervention variables that influenced selection likely to be influenced by the outcome or a cause of the outcome? |  | NA / Y / PY / PN / N / NI  NA / Y / PY / PN / N / NI |
|  | 2.4. Do start of follow-up and start of intervention coincide for most participants? | Y | Y / PY / PN / N / NI |
|  | 2.5. **If Y/PY to 2.2 and 2.3, or N/PN to 2.4**: Were adjustment techniques used that are likely to correct for the presence of selection biases? |  | NA / Y / PY / PN / N / NI |
|  | **Risk of bias judgement** | Low | Low / Moderate / Serious / Critical / NI |
|  | Optional: What is the predicted direction of bias due to selection of participants into the study? |  | Favours experimental / Favours comparator / Towards null /Away from null / Unpredictable |

| **Bias in classification of interventions** | | | |
| --- | --- | --- | --- |
|  | 3.1 Were intervention groups clearly defined? | Y: LTC facility | Y / PY / PN / N / NI |
|  | 3.2 Was the information used to define intervention groups recorded at the start of the intervention? | Y | Y / PY / PN / N / NI |
|  | 3.3 Could classification of intervention status have been affected by knowledge of the outcome or risk of the outcome? | N | Y / PY / PN / N / NI |
|  | **Risk of bias judgement** | Low | Low / Moderate / Serious / Critical / NI |
|  | Optional: What is the predicted direction of bias due to classification of interventions? |  | Favours experimental / Favours comparator / Towards null /Away from null / Unpredictable |

| **Bias due to deviations from intended interventions** | | | |
| --- | --- | --- | --- |
|  | **If your aim for this study is to assess the effect of assignment to intervention, answer questions 4.1 and 4.2** | |  |
|  | 4.1. Were there deviations from the intended intervention beyond what would be expected in usual practice? | N | Y / PY / PN / N / NI |
|  | 4.2. **If Y/PY to 4.1**: Were these deviations from intended intervention unbalanced between groups *and* likely to have affected the outcome? |  | NA / Y / PY / PN / N / NI |
|  | **If your aim for this study is to assess the effect of starting and adhering to intervention, answer questions 4.3 to 4.6** | |  |
|  | 4.3. Were important co-interventions balanced across intervention groups? | NA – no significant co-interventions | Y / PY / PN / N / NI |
|  | 4.4. Was the intervention implemented successfully for most participants? | Y | Y / PY / PN / N / NI |
|  | 4.5. Did study participants adhere to the assigned intervention regimen? | Y – 100% adherence | Y / PY / PN / N / NI |
|  | 4.6. **If N/PN to 4.3, 4.4 or 4.5**: Was an appropriate analysis used to estimate the effect of starting and adhering to the intervention? |  | NA / Y / PY / PN / N / NI |
|  | **Risk of bias judgement** | Low |  |
|  | Optional: What is the predicted direction of bias due to deviations from the intended interventions? |  |  |

| **Bias due to missing data** | | | |
| --- | --- | --- | --- |
|  | 5.1 Were outcome data available for all, or nearly all, participants? | PY; there appears to be unpublished data (in terms of culture, patient characteristics etc; secondary data); however, it is a preliminary paper. Authors do not appear to be hiding data. | Y / PY / PN / N / NI |
|  | 5.2 Were participants excluded due to missing data on intervention status? | N | Y / PY / PN / N / NI |
|  | 5.3 Were participants excluded due to missing data on other variables needed for the analysis? | N | Y / PY / PN / N / NI |
|  | 5.4 **If PN/N to 5.1, or Y/PY to 5.2 or 5.3**: Are the proportion of participants and reasons for missing data similar across interventions? |  | NA / Y / PY / PN / N / NI |
|  | 5.5 **If PN/N to 5.1, or Y/PY to 5.2 or 5.3**: Is there evidence that results were robust to the presence of missing data? |  | NA / Y / PY / PN / N / NI |
|  | **Risk of bias judgement** | Moderate | Low / Moderate / Serious / Critical / NI |
|  | Optional: What is the predicted direction of bias due to missing data? |  | Favours experimental / Favours comparator / Towards null /Away from null / Unpredictable |

| **Bias in measurement of outcomes** | | | |
| --- | --- | --- | --- |
|  | 6.1 Could the outcome measure have been influenced by knowledge of the intervention received? | N | Y / PY / PN / N / NI |
|  | 6.2 Were outcome assessors aware of the intervention received by study participants? | Y. study characteristics – not possible to hide intervention condition since there is addition of a pharmacist. | Y / PY / PN / N / NI |
|  | 6.3 Were the methods of outcome assessment comparable across intervention groups? | Y | Y / PY / PN / N / NI |
|  | 6.4 Were any systematic errors in measurement of the outcome related to intervention received? | N | Y / PY / PN / N / NI |
|  | **Risk of bias judgement** | Moderate | Low / Moderate / Serious / Critical / NI |
|  | Optional: What is the predicted direction of bias due to measurement of outcomes? |  | Favours experimental / Favours comparator / Towards null /Away from null / Unpredictable |

| **Bias in selection of the reported result** | | | |
| --- | --- | --- | --- |
|  | Is the reported effect estimate likely to be selected, on the basis of the results, from... |  |  |
|  | 7.1. ... multiple outcome *measurements* within the outcome domain? | N | Y / PY / PN / N / NI |
|  | 7.2 ... multiple *analyses* of the intervention-outcome relationship? | PN. | Y / PY / PN / N / NI |
|  | 7.3 ... different *subgroups*? | N | Y / PY / PN / N / NI |
|  | **Risk of bias judgement** | Low | Low / Moderate / Serious / Critical / NI |
|  | Optional: What is the predicted direction of bias due to selection of the reported result? |  | Favours experimental / Favours comparator / Towards null /Away from null / Unpredictable |

| **Overall bias** | | | |
| --- | --- | --- | --- |
|  | **Risk of bias judgement** | Serious – sample size. | Low / Moderate / Serious / Critical / NI |
|  | Optional: What is the overall predicted direction of bias for this outcome? |  | Favours experimental / Favours comparator / Towards null /Away from null / Unpredictable |

**Hutt et al 2006**

Responses underlined in green are potential markers for low risk of bias, and responses in red are potential markers for a risk of bias. Where questions relate only to sign posts to other questions, no formatting is used.

|  | **Signalling questions** | **Description** | **Response options** |
| --- | --- | --- | --- |
| **Bias due to confounding** | | | |
|  | 1.1 Is there potential for confounding of the effect of intervention in this study?  **If N/PN to 1.1:** the study can be considered to be at low risk of bias due to confounding and no further signalling questions need be considered | Y | Y / PY / PN / N |
|  | **If Y/PY to 1.1**: determine whether there is a need to assess time-varying confounding: |  |  |
|  | 1.2. Was the analysis based on splitting participants’ follow up time according to intervention received?  **If N/PN**, answer questions relating to baseline confounding (1.4 to 1.6)  **If Y/PY**, go to question 1.3. | N | NA / Y / PY / PN / N / NI |
|  | 1.3. Were intervention discontinuations or switches likely to be related to factors that are prognostic for the outcome?  **If N/PN**, answer questions relating to baseline confounding (1.4 to 1.6)  **If Y/PY**, answer questions relating to both baseline and time-varying confounding (1.7 and 1.8) |  | NA / Y / PY / PN / N / NI |

|  | **Questions relating to baseline confounding only** | | |
| --- | --- | --- | --- |
|  | 1.4. Did the authors use an appropriate analysis method that controlled for all the important confounding domains? | PN; no control for the fact that intervention site is 30 miles away from the hospital while control site is at hospital. Aside from that, no significant difference between participants.  Furthermore, despite claims of similarity between sites, there is a lack of statistical data showing difference between intervention sites – on first glance, preintervention data seems to suggest differences in baseline data. | NA / Y / PY / PN / N / NI |
|  | 1.5. **If Y/PY to 1.4**: Were confounding domains that were controlled for measured validly and reliably by the variables available in this study? |  | NA / Y / PY / PN / N / NI |
|  | 1.6. Did the authors control for any post-intervention variables that could have been affected by the intervention? | N | NA / Y / PY / PN / N / NI |
|  | **Questions relating to baseline and time-varying confounding** | |  |
|  | 1.7. Did the authors use an appropriate analysis method that controlled for all the important confounding domains and for time-varying confounding? |  | NA / Y / PY / PN / N / NI |
|  | 1.8. **If Y/PY to 1.7**: Were confounding domains that were controlled for measured validly and reliably by the variables available in this study? |  | NA / Y / PY / PN / N / NI |
|  | **Risk of bias judgement** | Moderate – proximity to hospital a confound | Low / Moderate / Serious / Critical / NI |
|  | Optional: What is the predicted direction of bias due to confounding? |  | Favours experimental / Favours comparator / Unpredictable |

| **Bias in selection of participants into the study** | | | |
| --- | --- | --- | --- |
|  | 2.1. Was selection of participants into the study (or into the analysis) based on participant characteristics observed after the start of intervention?  **If N/PN to 2.1:** go to 2.4 | N | Y / PY / PN / N / NI |
|  | 2.2. **If Y/PY to 2.1**: Were the post-intervention variables that influenced selection likely to be associated with intervention?  2.3 **If Y/PY to 2.2**: Were the post-intervention variables that influenced selection likely to be influenced by the outcome or a cause of the outcome? |  | NA / Y / PY / PN / N / NI  NA / Y / PY / PN / N / NI |
|  | 2.4. Do start of follow-up and start of intervention coincide for most participants? | Y | Y / PY / PN / N / NI |
|  | 2.5. **If Y/PY to 2.2 and 2.3, or N/PN to 2.4**: Were adjustment techniques used that are likely to correct for the presence of selection biases? |  | NA / Y / PY / PN / N / NI |
|  | **Risk of bias judgement** | Low | Low / Moderate / Serious / Critical / NI |
|  | Optional: What is the predicted direction of bias due to selection of participants into the study? |  | Favours experimental / Favours comparator / Towards null /Away from null / Unpredictable |

| **Bias in classification of interventions** | | | |
| --- | --- | --- | --- |
|  | 3.1 Were intervention groups clearly defined? | Y, by NH with Pre/post test comparisons with untreated group. | Y / PY / PN / N / NI |
|  | 3.2 Was the information used to define intervention groups recorded at the start of the intervention? | Y | Y / PY / PN / N / NI |
|  | 3.3 Could classification of intervention status have been affected by knowledge of the outcome or risk of the outcome? | N | Y / PY / PN / N / NI |
|  | **Risk of bias judgement** | Low | Low / Moderate / Serious / Critical / NI |
|  | Optional: What is the predicted direction of bias due to classification of interventions? |  | Favours experimental / Favours comparator / Towards null /Away from null / Unpredictable |

| **Bias due to deviations from intended interventions** | | | |
| --- | --- | --- | --- |
|  | **If your aim for this study is to assess the effect of assignment to intervention, answer questions 4.1 and 4.2** | |  |
|  | 4.1. Were there deviations from the intended intervention beyond what would be expected in usual practice? | N | Y / PY / PN / N / NI |
|  | 4.2. **If Y/PY to 4.1**: Were these deviations from intended intervention unbalanced between groups *and* likely to have affected the outcome? |  | NA / Y / PY / PN / N / NI |
|  | **If your aim for this study is to assess the effect of starting and adhering to intervention, answer questions 4.3 to 4.6** | |  |
|  | 4.3. Were important co-interventions balanced across intervention groups? | N | Y / PY / PN / N / NI |
|  | 4.4. Was the intervention implemented successfully for most participants? | Y | Y / PY / PN / N / NI |
|  | 4.5. Did study participants adhere to the assigned intervention regimen? | PN; Low adherence; not all delivered intervention was carried out; particularly, adherence was low (hovering around 50% for in-service).  However, certainly policies (such as printing of pre-orders, occurred.) On the other hand, control implemented policies that was coincidentally part of intervention (staff flu shot policy) | Y / PY / PN / N / NI |
|  | 4.6. **If N/PN to 4.3, 4.4 or 4.5**: Was an appropriate analysis used to estimate the effect of starting and adhering to the intervention? | N | NA / Y / PY / PN / N / NI |
|  | **Risk of bias judgement** | Serious/Moderate |  |
|  | Optional: What is the predicted direction of bias due to deviations from the intended interventions? |  |  |

| **Bias due to missing data** | | | |
| --- | --- | --- | --- |
|  | 5.1 Were outcome data available for all, or nearly all, participants? | Y; | Y / PY / PN / N / NI |
|  | 5.2 Were participants excluded due to missing data on intervention status? | N | Y / PY / PN / N / NI |
|  | 5.3 Were participants excluded due to missing data on other variables needed for the analysis? | N | Y / PY / PN / N / NI |
|  | 5.4 **If PN/N to 5.1, or Y/PY to 5.2 or 5.3**: Are the proportion of participants and reasons for missing data similar across interventions? |  | NA / Y / PY / PN / N / NI |
|  | 5.5 **If PN/N to 5.1, or Y/PY to 5.2 or 5.3**: Is there evidence that results were robust to the presence of missing data? |  | NA / Y / PY / PN / N / NI |
|  | **Risk of bias judgement** | Low | Low / Moderate / Serious / Critical / NI |
|  | Optional: What is the predicted direction of bias due to missing data? |  | Favours experimental / Favours comparator / Towards null /Away from null / Unpredictable |

| **Bias in measurement of outcomes** | | | |
| --- | --- | --- | --- |
|  | 6.1 Could the outcome measure have been influenced by knowledge of the intervention received? | PY: staff is likely to know there is an intervention policy since education, immunization etc were introduced. | Y / PY / PN / N / NI |
|  | 6.2 Were outcome assessors aware of the intervention received by study participants? | Y – difficult for a blind study | Y / PY / PN / N / NI |
|  | 6.3 Were the methods of outcome assessment comparable across intervention groups? | Y | Y / PY / PN / N / NI |
|  | 6.4 Were any systematic errors in measurement of the outcome related to intervention received? | N | Y / PY / PN / N / NI |
|  | **Risk of bias judgement** | Moderate | Low / Moderate / Serious / Critical / NI |
|  | Optional: What is the predicted direction of bias due to measurement of outcomes? |  | Favours experimental / Favours comparator / Towards null /Away from null / Unpredictable |

| **Bias in selection of the reported result** | | | |
| --- | --- | --- | --- |
|  | Is the reported effect estimate likely to be selected, on the basis of the results, from... |  |  |
|  | 7.1. ... multiple outcome *measurements* within the outcome domain? | PY; possibility of picking certain data. See below | Y / PY / PN / N / NI |
|  | 7.2 ... multiple *analyses* of the intervention-outcome relationship? | PY; Cherry picking of analysis. For instance, despite the claim that intervention facility improved vaccination rate by nearly 25% ‘in a year when vaccine was difficult to obtain’ (not backed up), flu shot policy for staff was implemented in the control facility but not the vaccination facility.  It is also possible that flu shot vaccine numbers increased because prior numbers were low – it was at 71, compared to 100 at control facility. | Y / PY / PN / N / NI |
|  | 7.3 ... different *subgroups*? | N | Y / PY / PN / N / NI |
|  | **Risk of bias judgement** | Moderate | Low / Moderate / Serious / Critical / NI |
|  | Optional: What is the predicted direction of bias due to selection of the reported result? | Favour experimental | Favours experimental / Favours comparator / Towards null /Away from null / Unpredictable |

| **Overall bias** | | | |
| --- | --- | --- | --- |
|  | **Risk of bias judgement** | Serious | Low / Moderate / Serious / Critical / NI |
|  | Optional: What is the overall predicted direction of bias for this outcome? | Favours experimental | Favours experimental / Favours comparator / Towards null /Away from null / Unpredictable |

**Hutt et al 2011**

Responses underlined in green are potential markers for low risk of bias, and responses in red are potential markers for a risk of bias. Where questions relate only to sign posts to other questions, no formatting is used.

|  | **Signalling questions** | **Description** | **Response options** |
| --- | --- | --- | --- |
| **Bias due to confounding** | | | |
|  | 1.1 Is there potential for confounding of the effect of intervention in this study?  **If N/PN to 1.1:** the study can be considered to be at low risk of bias due to confounding and no further signalling questions need be considered | Y. Pre/post intervention comparison done over flu seasons. intervention facilities were paid an additional $1000/year to "incentivize guideline compliance and early detection of LRTI signs and symptoms". Suggestion for differential treatment for detection (increased effort, perhaps?) which may affect patient expectancy | Y / PY / PN / N |
|  | **If Y/PY to 1.1**: determine whether there is a need to assess time-varying confounding: |  |  |
|  | 1.2. Was the analysis based on splitting participants’ follow up time according to intervention received?  **If N/PN**, answer questions relating to baseline confounding (1.4 to 1.6)  **If Y/PY**, go to question 1.3. | N | NA / Y / PY / PN / N / NI |
|  | 1.3. Were intervention discontinuations or switches likely to be related to factors that are prognostic for the outcome?  **If N/PN**, answer questions relating to baseline confounding (1.4 to 1.6)  **If Y/PY**, answer questions relating to both baseline and time-varying confounding (1.7 and 1.8) |  | NA / Y / PY / PN / N / NI |

|  | **Questions relating to baseline confounding only** | | |
| --- | --- | --- | --- |
|  | 1.4. Did the authors use an appropriate analysis method that controlled for all the important confounding domains? | PN.  Despite attempts of the author to match, choice of nursing homes resulted in statistical significant differences in racial distribution, Functional status, severity score, nursing staff turnover, and nurse/resident hours per day. In general, it appeared that control homes were a lot less well-funded than intervention homes, which is even more questionable (more minorities; less hours; patients less independent etc) when considering the additional funding Intervention homes are given.  Although the author noted the differences, it was weakly justified by similar levels of mortality. nonetheless, the demographic differences is of concern. | NA / Y / PY / PN / N / NI |
|  | 1.5. **If Y/PY to 1.4**: Were confounding domains that were controlled for measured validly and reliably by the variables available in this study? |  | NA / Y / PY / PN / N / NI |
|  | 1.6. Did the authors control for any post-intervention variables that could have been affected by the intervention? | N | NA / Y / PY / PN / N / NI |
|  | **Questions relating to baseline and time-varying confounding** | |  |
|  | 1.7. Did the authors use an appropriate analysis method that controlled for all the important confounding domains and for time-varying confounding? |  | NA / Y / PY / PN / N / NI |
|  | 1.8. **If Y/PY to 1.7**: Were confounding domains that were controlled for measured validly and reliably by the variables available in this study? |  | NA / Y / PY / PN / N / NI |
|  | **Risk of bias judgement** | Moderate | Low / Moderate / Serious / Critical / NI |
|  | Optional: What is the predicted direction of bias due to confounding? |  | Favours experimental / Favours comparator / Unpredictable |

| **Bias in selection of participants into the study** | | | |
| --- | --- | --- | --- |
|  | 2.1. Was selection of participants into the study (or into the analysis) based on participant characteristics observed after the start of intervention?  **If N/PN to 2.1:** go to 2.4 | N | Y / PY / PN / N / NI |
|  | 2.2. **If Y/PY to 2.1**: Were the post-intervention variables that influenced selection likely to be associated with intervention?  2.3 **If Y/PY to 2.2**: Were the post-intervention variables that influenced selection likely to be influenced by the outcome or a cause of the outcome? |  | NA / Y / PY / PN / N / NI  NA / Y / PY / PN / N / NI |
|  | 2.4. Do start of follow-up and start of intervention coincide for most participants? | Y | Y / PY / PN / N / NI |
|  | 2.5. **If Y/PY to 2.2 and 2.3, or N/PN to 2.4**: Were adjustment techniques used that are likely to correct for the presence of selection biases? |  | NA / Y / PY / PN / N / NI |
|  | **Risk of bias judgement** | Low | Low / Moderate / Serious / Critical / NI |
|  | Optional: What is the predicted direction of bias due to selection of participants into the study? |  | Favours experimental / Favours comparator / Towards null /Away from null / Unpredictable |

| **Bias in classification of interventions** | | | |
| --- | --- | --- | --- |
|  | 3.1 Were intervention groups clearly defined? | Y – by NH. | Y / PY / PN / N / NI |
|  | 3.2 Was the information used to define intervention groups recorded at the start of the intervention? | Y | Y / PY / PN / N / NI |
|  | 3.3 Could classification of intervention status have been affected by knowledge of the outcome or risk of the outcome? | N | Y / PY / PN / N / NI |
|  | **Risk of bias judgement** | Low | Low / Moderate / Serious / Critical / NI |
|  | Optional: What is the predicted direction of bias due to classification of interventions? |  | Favours experimental / Favours comparator / Towards null /Away from null / Unpredictable |

| **Bias due to deviations from intended interventions** | | | |
| --- | --- | --- | --- |
|  | **If your aim for this study is to assess the effect of assignment to intervention, answer questions 4.1 and 4.2** | |  |
|  | 4.1. Were there deviations from the intended intervention beyond what would be expected in usual practice? | N | Y / PY / PN / N / NI |
|  | 4.2. **If Y/PY to 4.1**: Were these deviations from intended intervention unbalanced between groups *and* likely to have affected the outcome? |  | NA / Y / PY / PN / N / NI |
|  | **If your aim for this study is to assess the effect of starting and adhering to intervention, answer questions 4.3 to 4.6** | |  |
|  | 4.3. Were important co-interventions balanced across intervention groups? | N | Y / PY / PN / N / NI |
|  | 4.4. Was the intervention implemented successfully for most participants? | N: | Y / PY / PN / N / NI |
|  | 4.5. Did study participants adhere to the assigned intervention regimen? | Y | Y / PY / PN / N / NI |
|  | 4.6. **If N/PN to 4.3, 4.4 or 4.5**: Was an appropriate analysis used to estimate the effect of starting and adhering to the intervention? | 4.3: Y – use of Fichter’s test  4.4: N – Poor adherence in recommendation of hospitalization to critically ill residents. | NA / Y / PY / PN / N / NI |
|  | **Risk of bias judgement** | Serious |  |
|  | Optional: What is the predicted direction of bias due to deviations from the intended interventions? |  |  |

| **Bias due to missing data** | | | |
| --- | --- | --- | --- |
|  | 5.1 Were outcome data available for all, or nearly all, participants? | Y | Y / PY / PN / N / NI |
|  | 5.2 Were participants excluded due to missing data on intervention status? | N | Y / PY / PN / N / NI |
|  | 5.3 Were participants excluded due to missing data on other variables needed for the analysis? | N | Y / PY / PN / N / NI |
|  | 5.4 **If PN/N to 5.1, or Y/PY to 5.2 or 5.3**: Are the proportion of participants and reasons for missing data similar across interventions? |  | NA / Y / PY / PN / N / NI |
|  | 5.5 **If PN/N to 5.1, or Y/PY to 5.2 or 5.3**: Is there evidence that results were robust to the presence of missing data? |  | NA / Y / PY / PN / N / NI |
|  | **Risk of bias judgement** | Low | Low / Moderate / Serious / Critical / NI |
|  | Optional: What is the predicted direction of bias due to missing data? |  | Favours experimental / Favours comparator / Towards null /Away from null / Unpredictable |

| **Bias in measurement of outcomes** | | | |
| --- | --- | --- | --- |
|  | 6.1 Could the outcome measure have been influenced by knowledge of the intervention received? | Y – cannot be controlled, as interventions are relatively salient. | Y / PY / PN / N / NI |
|  | 6.2 Were outcome assessors aware of the intervention received by study participants? | Y – cannot be controlled. Intervention introduced academic detailing among other policies. | Y / PY / PN / N / NI |
|  | 6.3 Were the methods of outcome assessment comparable across intervention groups? | Y | Y / PY / PN / N / NI |
|  | 6.4 Were any systematic errors in measurement of the outcome related to intervention received? | N | Y / PY / PN / N / NI |
|  | **Risk of bias judgement** | Moderate | Low / Moderate / Serious / Critical / NI |
|  | Optional: What is the predicted direction of bias due to measurement of outcomes? |  | Favours experimental / Favours comparator / Towards null /Away from null / Unpredictable |

| **Bias in selection of the reported result** | | | |
| --- | --- | --- | --- |
|  | Is the reported effect estimate likely to be selected, on the basis of the results, from... |  |  |
|  | 7.1. ... multiple outcome *measurements* within the outcome domain? | PY – primary outcome data not shown; secondary outcome data also not shown, but reported as findings (Chest X-ray). However, this is possibly due to the fact that there’s no significant differences between these data. | Y / PY / PN / N / NI |
|  | 7.2 ... multiple *analyses* of the intervention-outcome relationship? | PY – selective analysis of data (see above) | Y / PY / PN / N / NI |
|  | 7.3 ... different *subgroups*? | N | Y / PY / PN / N / NI |
|  | **Risk of bias judgement** | Serious | Low / Moderate / Serious / Critical / NI |
|  | Optional: What is the predicted direction of bias due to selection of the reported result? |  | Favours experimental / Favours comparator / Towards null /Away from null / Unpredictable |

| **Overall bias** | | | |
| --- | --- | --- | --- |
|  | **Risk of bias judgement** | Serious | Low / Moderate / Serious / Critical / NI |
|  | Optional: What is the overall predicted direction of bias for this outcome? |  | Favours experimental / Favours comparator / Towards null /Away from null / Unpredictable |

**Jump et al 2013**

Responses underlined in green are potential markers for low risk of bias, and responses in red are potential markers for a risk of bias. Where questions relate only to sign posts to other questions, no formatting is used.

|  | **Signalling questions** | **Description** | **Response options** |
| --- | --- | --- | --- |
| **Bias due to confounding** | | | |
|  | 1.1 Is there potential for confounding of the effect of intervention in this study?  **If N/PN to 1.1:** the study can be considered to be at low risk of bias due to confounding and no further signalling questions need be considered | Y | Y / PY / PN / N |
|  | **If Y/PY to 1.1**: determine whether there is a need to assess time-varying confounding: |  |  |
|  | 1.2. Was the analysis based on splitting participants’ follow up time according to intervention received?  **If N/PN**, answer questions relating to baseline confounding (1.4 to 1.6)  **If Y/PY**, go to question 1.3. | PY; as LID’s recommendations concern patients, patients may choose not to follow LID’s treatment plan. For instance, LID may recommend a patient to stay at a hospital but a patient may insist on staying (under LTC’s initial guidance; thus non-intervention) | NA / Y / PY / PN / N / NI |
|  | 1.3. Were intervention discontinuations or switches likely to be related to factors that are prognostic for the outcome?  **If N/PN**, answer questions relating to baseline confounding (1.4 to 1.6)  **If Y/PY**, answer questions relating to both baseline and time-varying confounding (1.7 and 1.8) | PY | NA / Y / PY / PN / N / NI |

|  | **Questions relating to baseline confounding only** | | |
| --- | --- | --- | --- |
|  | 1.4. Did the authors use an appropriate analysis method that controlled for all the important confounding domains? |  | NA / Y / PY / PN / N / NI |
|  | 1.5. **If Y/PY to 1.4**: Were confounding domains that were controlled for measured validly and reliably by the variables available in this study? |  | NA / Y / PY / PN / N / NI |
|  | 1.6. Did the authors control for any post-intervention variables that could have been affected by the intervention? |  | NA / Y / PY / PN / N / NI |
|  | **Questions relating to baseline and time-varying confounding** | |  |
|  | 1.7. Did the authors use an appropriate analysis method that controlled for all the important confounding domains and for time-varying confounding? | N; it appeared that the authors did not have a pre-post intervention setting, instead basing results on changes (in terms of AB prescription) to initial diagnosis by the CLC staff. No information on switches or patients’ choices. | NA / Y / PY / PN / N / NI |
|  | 1.8. **If Y/PY to 1.7**: Were confounding domains that were controlled for measured validly and reliably by the variables available in this study? |  | NA / Y / PY / PN / N / NI |
|  | **Risk of bias judgement** | Serious | Low / Moderate / Serious / Critical / NI |
|  | Optional: What is the predicted direction of bias due to confounding? |  | Favours experimental / Favours comparator / Unpredictable |

| **Bias in selection of participants into the study** | | | |
| --- | --- | --- | --- |
|  | 2.1. Was selection of participants into the study (or into the analysis) based on participant characteristics observed after the start of intervention?  **If N/PN to 2.1:** go to 2.4 | Y; selection was based on LTC (or hospital) staff’s recommendation. | Y / PY / PN / N / NI |
|  | 2.2. **If Y/PY to 2.1**: Were the post-intervention variables that influenced selection likely to be associated with intervention?  2.3 **If Y/PY to 2.2**: Were the post-intervention variables that influenced selection likely to be influenced by the outcome or a cause of the outcome? | Y; As the paper stated, there appear to be a need for LID’s in LTCs. Selection by the LTC staff for LID’s recommendation is likely to be based on either AB requirements (eg. Vancomycin dosage), or seriousness/uncommon symptoms. This suggests a selection bias. | NA / Y / PY / PN / N / NI  NA / Y / PY / PN / N / NI |
|  | 2.4. Do start of follow-up and start of intervention coincide for most participants? | Y | Y / PY / PN / N / NI |
|  | 2.5. **If Y/PY to 2.2 and 2.3, or N/PN to 2.4**: Were adjustment techniques used that are likely to correct for the presence of selection biases? |  | NA / Y / PY / PN / N / NI |
|  | **Risk of bias judgement** | Critical | Low / Moderate / Serious / Critical / NI |
|  | Optional: What is the predicted direction of bias due to selection of participants into the study? |  | Favours experimental / Favours comparator / Towards null /Away from null / Unpredictable |

| **Bias in classification of interventions** | | | |
| --- | --- | --- | --- |
|  | 3.1 Were intervention groups clearly defined? | N; based on LTC/hospital staff recommendation | Y / PY / PN / N / NI |
|  | 3.2 Was the information used to define intervention groups recorded at the start of the intervention? | Y | Y / PY / PN / N / NI |
|  | 3.3 Could classification of intervention status have been affected by knowledge of the outcome or risk of the outcome? | PY | Y / PY / PN / N / NI |
|  | **Risk of bias judgement** | Serious | Low / Moderate / Serious / Critical / NI |
|  | Optional: What is the predicted direction of bias due to classification of interventions? |  | Favours experimental / Favours comparator / Towards null /Away from null / Unpredictable |

| **Bias due to deviations from intended interventions** | | | |
| --- | --- | --- | --- |
|  | **If your aim for this study is to assess the effect of assignment to intervention, answer questions 4.1 and 4.2** | |  |
|  | 4.1. Were there deviations from the intended intervention beyond what would be expected in usual practice? | Y; lack of comparison or pre/post intervention comparisons. | Y / PY / PN / N / NI |
|  | 4.2. **If Y/PY to 4.1**: Were these deviations from intended intervention unbalanced between groups *and* likely to have affected the outcome? | Y | NA / Y / PY / PN / N / NI |
|  | **If your aim for this study is to assess the effect of starting and adhering to intervention, answer questions 4.3 to 4.6** | |  |
|  | 4.3. Were important co-interventions balanced across intervention groups? | N: although the LID consultation service’s main purpose was consultation only, “they had direct communication with providers and could place orders pertaining to antibiotic and related medication, as well as diagnostic studies and laboratory requests.” | Y / PY / PN / N / NI |
|  | 4.4. Was the intervention implemented successfully for most participants? | Y | Y / PY / PN / N / NI |
|  | 4.5. Did study participants adhere to the assigned intervention regimen? | Y | Y / PY / PN / N / NI |
|  | 4.6. **If N/PN to 4.3, 4.4 or 4.5**: Was an appropriate analysis used to estimate the effect of starting and adhering to the intervention? | No | NA / Y / PY / PN / N / NI |
|  | **Risk of bias judgement** | Moderate |  |
|  | Optional: What is the predicted direction of bias due to deviations from the intended interventions? |  |  |

| **Bias due to missing data** | | | |
| --- | --- | --- | --- |
|  | 5.1 Were outcome data available for all, or nearly all, participants? | Y | Y / PY / PN / N / NI |
|  | 5.2 Were participants excluded due to missing data on intervention status? | N | Y / PY / PN / N / NI |
|  | 5.3 Were participants excluded due to missing data on other variables needed for the analysis? | N | Y / PY / PN / N / NI |
|  | 5.4 **If PN/N to 5.1, or Y/PY to 5.2 or 5.3**: Are the proportion of participants and reasons for missing data similar across interventions? |  | NA / Y / PY / PN / N / NI |
|  | 5.5 **If PN/N to 5.1, or Y/PY to 5.2 or 5.3**: Is there evidence that results were robust to the presence of missing data? |  | NA / Y / PY / PN / N / NI |
|  | **Risk of bias judgement** | Low | Low / Moderate / Serious / Critical / NI |
|  | Optional: What is the predicted direction of bias due to missing data? |  | Favours experimental / Favours comparator / Towards null /Away from null / Unpredictable |

| **Bias in measurement of outcomes** | | | |
| --- | --- | --- | --- |
|  | 6.1 Could the outcome measure have been influenced by knowledge of the intervention received? | N | Y / PY / PN / N / NI |
|  | 6.2 Were outcome assessors aware of the intervention received by study participants? | Y. Not controlled for. | Y / PY / PN / N / NI |
|  | 6.3 Were the methods of outcome assessment comparable across intervention groups? | Y | Y / PY / PN / N / NI |
|  | 6.4 Were any systematic errors in measurement of the outcome related to intervention received? | Y; likely that selection bias have an impact on measurement of outcome of intervention group | Y / PY / PN / N / NI |
|  | **Risk of bias judgement** | Moderate | Low / Moderate / Serious / Critical / NI |
|  | Optional: What is the predicted direction of bias due to measurement of outcomes? |  | Favours experimental / Favours comparator / Towards null /Away from null / Unpredictable |

| **Bias in selection of the reported result** | | | |
| --- | --- | --- | --- |
|  | Is the reported effect estimate likely to be selected, on the basis of the results, from... |  |  |
|  | 7.1. ... multiple outcome *measurements* within the outcome domain? | N | Y / PY / PN / N / NI |
|  | 7.2 ... multiple *analyses* of the intervention-outcome relationship? | N | Y / PY / PN / N / NI |
|  | 7.3 ... different *subgroups*? | N | Y / PY / PN / N / NI |
|  | **Risk of bias judgement** | Low | Low / Moderate / Serious / Critical / NI |
|  | Optional: What is the predicted direction of bias due to selection of the reported result? |  | Favours experimental / Favours comparator / Towards null /Away from null / Unpredictable |

| **Overall bias** | | | |
| --- | --- | --- | --- |
|  | **Risk of bias judgement** | Critical | Low / Moderate / Serious / Critical / NI |
|  | Optional: What is the overall predicted direction of bias for this outcome? |  | Favours experimental / Favours comparator / Towards null /Away from null / Unpredictable |

**Kassett et al 2016**

Responses underlined in green are potential markers for low risk of bias, and responses in red are potential markers for a risk of bias. Where questions relate only to sign posts to other questions, no formatting is used.

|  | **Signalling questions** | **Description** | **Response options** |
| --- | --- | --- | --- |
| **Bias due to confounding** | | | |
|  | 1.1 Is there potential for confounding of the effect of intervention in this study?  **If N/PN to 1.1:** the study can be considered to be at low risk of bias due to confounding and no further signalling questions need be considered | Y | Y / PY / PN / N |
|  | **If Y/PY to 1.1**: determine whether there is a need to assess time-varying confounding: |  |  |
|  | 1.2. Was the analysis based on splitting participants’ follow up time according to intervention received?  **If N/PN**, answer questions relating to baseline confounding (1.4 to 1.6)  **If Y/PY**, go to question 1.3. | N | NA / Y / PY / PN / N / NI |
|  | 1.3. Were intervention discontinuations or switches likely to be related to factors that are prognostic for the outcome?  **If N/PN**, answer questions relating to baseline confounding (1.4 to 1.6)  **If Y/PY**, answer questions relating to both baseline and time-varying confounding (1.7 and 1.8) |  | NA / Y / PY / PN / N / NI |

|  | **Questions relating to baseline confounding only** | | |
| --- | --- | --- | --- |
|  | 1.4. Did the authors use an appropriate analysis method that controlled for all the important confounding domains? | PY - α level of 0.05, t-test of Prescribed days of therapy vs Actual days of therapy | NA / Y / PY / PN / N / NI |
|  | 1.5. **If Y/PY to 1.4**: Were confounding domains that were controlled for measured validly and reliably by the variables available in this study? | N; the two facilities were in the same building. There is a possibility of patients being ‘double-treated’, even though the possibility may be low. However, due to lack of patient identification, this issue becomes a possible confound. | NA / Y / PY / PN / N / NI |
|  | 1.6. Did the authors control for any post-intervention variables that could have been affected by the intervention? | N | NA / Y / PY / PN / N / NI |
|  | **Questions relating to baseline and time-varying confounding** | |  |
|  | 1.7. Did the authors use an appropriate analysis method that controlled for all the important confounding domains and for time-varying confounding? |  | NA / Y / PY / PN / N / NI |
|  | 1.8. **If Y/PY to 1.7**: Were confounding domains that were controlled for measured validly and reliably by the variables available in this study? |  | NA / Y / PY / PN / N / NI |
|  | **Risk of bias judgement** | Moderate | Low / Moderate / Serious / Critical / NI |
|  | Optional: What is the predicted direction of bias due to confounding? |  | Favours experimental / Favours comparator / Unpredictable |

| **Bias in selection of participants into the study** | | | |
| --- | --- | --- | --- |
|  | 2.1. Was selection of participants into the study (or into the analysis) based on participant characteristics observed after the start of intervention?  **If N/PN to 2.1:** go to 2.4 | N | Y / PY / PN / N / NI |
|  | 2.2. **If Y/PY to 2.1**: Were the post-intervention variables that influenced selection likely to be associated with intervention?  2.3 **If Y/PY to 2.2**: Were the post-intervention variables that influenced selection likely to be influenced by the outcome or a cause of the outcome? |  | NA / Y / PY / PN / N / NI  NA / Y / PY / PN / N / NI |
|  | 2.4. Do start of follow-up and start of intervention coincide for most participants? | Y | Y / PY / PN / N / NI |
|  | 2.5. **If Y/PY to 2.2 and 2.3, or N/PN to 2.4**: Were adjustment techniques used that are likely to correct for the presence of selection biases? |  | NA / Y / PY / PN / N / NI |
|  | **Risk of bias judgement** | Low | Low / Moderate / Serious / Critical / NI |
|  | Optional: What is the predicted direction of bias due to selection of participants into the study? |  | Favours experimental / Favours comparator / Towards null /Away from null / Unpredictable |

| **Bias in classification of interventions** | | | |
| --- | --- | --- | --- |
|  | 3.1 Were intervention groups clearly defined? | PN; UTI patients/patients using a list of UTI medications. However, patients may be suffering from other ailments but using same medication. – Paper also mentioned, that the medication could be used for other stuffs. | Y / PY / PN / N / NI |
|  | 3.2 Was the information used to define intervention groups recorded at the start of the intervention? | Y | Y / PY / PN / N / NI |
|  | 3.3 Could classification of intervention status have been affected by knowledge of the outcome or risk of the outcome? | PY; not possible to blind participants due to semination of information. Possible confound. | Y / PY / PN / N / NI |
|  | **Risk of bias judgement** | Serious | Low / Moderate / Serious / Critical / NI |
|  | Optional: What is the predicted direction of bias due to classification of interventions? |  | Favours experimental / Favours comparator / Towards null /Away from null / Unpredictable |

| **Bias due to deviations from intended interventions** | | | |
| --- | --- | --- | --- |
|  | **If your aim for this study is to assess the effect of assignment to intervention, answer questions 4.1 and 4.2** | |  |
|  | 4.1. Were there deviations from the intended intervention beyond what would be expected in usual practice? | N | Y / PY / PN / N / NI |
|  | 4.2. **If Y/PY to 4.1**: Were these deviations from intended intervention unbalanced between groups *and* likely to have affected the outcome? |  | NA / Y / PY / PN / N / NI |
|  | **If your aim for this study is to assess the effect of starting and adhering to intervention, answer questions 4.3 to 4.6** | |  |
|  | 4.3. Were important co-interventions balanced across intervention groups? | N: co-interventions not implemented. Possible bias in Education vs Audit and feedback vs Change to default stop dates. | Y / PY / PN / N / NI |
|  | 4.4. Was the intervention implemented successfully for most participants? | NI | Y / PY / PN / N / NI |
|  | 4.5. Did study participants adhere to the assigned intervention regimen? | NI | Y / PY / PN / N / NI |
|  | 4.6. **If N/PN to 4.3, 4.4 or 4.5**: Was an appropriate analysis used to estimate the effect of starting and adhering to the intervention? |  | NA / Y / PY / PN / N / NI |
|  | **Risk of bias judgement** | Moderate |  |
|  | Optional: What is the predicted direction of bias due to deviations from the intended interventions? |  |  |

| **Bias due to missing data** | | | |
| --- | --- | --- | --- |
|  | 5.1 Were outcome data available for all, or nearly all, participants? | Y | Y / PY / PN / N / NI |
|  | 5.2 Were participants excluded due to missing data on intervention status? | N | Y / PY / PN / N / NI |
|  | 5.3 Were participants excluded due to missing data on other variables needed for the analysis? | N | Y / PY / PN / N / NI |
|  | 5.4 **If PN/N to 5.1, or Y/PY to 5.2 or 5.3**: Are the proportion of participants and reasons for missing data similar across interventions? |  | NA / Y / PY / PN / N / NI |
|  | 5.5 **If PN/N to 5.1, or Y/PY to 5.2 or 5.3**: Is there evidence that results were robust to the presence of missing data? |  | NA / Y / PY / PN / N / NI |
|  | **Risk of bias judgement** | Low | Low / Moderate / Serious / Critical / NI |
|  | Optional: What is the predicted direction of bias due to missing data? |  | Favours experimental / Favours comparator / Towards null /Away from null / Unpredictable |

| **Bias in measurement of outcomes** | | | |
| --- | --- | --- | --- |
|  | 6.1 Could the outcome measure have been influenced by knowledge of the intervention received? | PY – being aware of receiving intervention may cause physicians to be more likely to adhere to treatment guidelines. | Y / PY / PN / N / NI |
|  | 6.2 Were outcome assessors aware of the intervention received by study participants? | Y; study design; introduction of intervention will be known be participants and assessors as it is a hospital wide policy. | Y / PY / PN / N / NI |
|  | 6.3 Were the methods of outcome assessment comparable across intervention groups? | Y | Y / PY / PN / N / NI |
|  | 6.4 Were any systematic errors in measurement of the outcome related to intervention received? | N | Y / PY / PN / N / NI |
|  | **Risk of bias judgement** | Moderate | Low / Moderate / Serious / Critical / NI |
|  | Optional: What is the predicted direction of bias due to measurement of outcomes? |  | Favours experimental / Favours comparator / Towards null /Away from null / Unpredictable |

| **Bias in selection of the reported result** | | | |
| --- | --- | --- | --- |
|  | Is the reported effect estimate likely to be selected, on the basis of the results, from... |  |  |
|  | 7.1. ... multiple outcome *measurements* within the outcome domain? | N | Y / PY / PN / N / NI |
|  | 7.2 ... multiple *analyses* of the intervention-outcome relationship? | N | Y / PY / PN / N / NI |
|  | 7.3 ... different *subgroups*? | N | Y / PY / PN / N / NI |
|  | **Risk of bias judgement** | Low | Low / Moderate / Serious / Critical / NI |
|  | Optional: What is the predicted direction of bias due to selection of the reported result? |  | Favours experimental / Favours comparator / Towards null /Away from null / Unpredictable |

| **Overall bias** | | | |
| --- | --- | --- | --- |
|  | **Risk of bias judgement** | Serious | Low / Moderate / Serious / Critical / NI |
|  | Optional: What is the overall predicted direction of bias for this outcome? |  | Favours experimental / Favours comparator / Towards null /Away from null / Unpredictable |

## Linnebur et al 2011

Responses underlined in green are potential markers for low risk of bias, and responses in red are potential markers for a risk of bias. Where questions relate only to sign posts to other questions, no formatting is used.

|  | **Signalling questions** | **Description** | **Response options** |
| --- | --- | --- | --- |
| **Bias due to confounding** | | | |
|  | 1.1 Is there potential for confounding of the effect of intervention in this study?  **If N/PN to 1.1:** the study can be considered to be at low risk of bias due to confounding and no further signalling questions need be considered | Y | Y / PY / PN / N |
|  | **If Y/PY to 1.1**: determine whether there is a need to assess time-varying confounding: |  |  |
|  | 1.2. Was the analysis based on splitting participants’ follow up time according to intervention received?  **If N/PN**, answer questions relating to baseline confounding (1.4 to 1.6)  **If Y/PY**, go to question 1.3. | N | NA / Y / PY / PN / N / NI |
|  | 1.3. Were intervention discontinuations or switches likely to be related to factors that are prognostic for the outcome?  **If N/PN**, answer questions relating to baseline confounding (1.4 to 1.6)  **If Y/PY**, answer questions relating to both baseline and time-varying confounding (1.7 and 1.8) |  | NA / Y / PY / PN / N / NI |

|  | **Questions relating to baseline confounding only** | | |
| --- | --- | --- | --- |
|  | 1.4. Did the authors use an appropriate analysis method that controlled for all the important confounding domains? | PN; baseline differences in control/intervention group, particularly, in mean adherence within 4 hrs (57% at baseline, pre-intervention, compared to 38% at control facilities, pre-intervention), were not accounted for. Critically, mean adherence within 4 hrs is an outcome variable that is measured and compared, thus severely weakening the validity of the study.  Similarly, another outcome baseline variable that differed was adherence to use of guideline recommended AB (60% at intervention v 32% at control). | NA / Y / PY / PN / N / NI |
|  | 1.5. **If Y/PY to 1.4**: Were confounding domains that were controlled for measured validly and reliably by the variables available in this study? |  | NA / Y / PY / PN / N / NI |
|  | 1.6. Did the authors control for any post-intervention variables that could have been affected by the intervention? |  | NA / Y / PY / PN / N / NI |
|  | **Questions relating to baseline and time-varying confounding** | |  |
|  | 1.7. Did the authors use an appropriate analysis method that controlled for all the important confounding domains and for time-varying confounding? |  | NA / Y / PY / PN / N / NI |
|  | 1.8. **If Y/PY to 1.7**: Were confounding domains that were controlled for measured validly and reliably by the variables available in this study? |  | NA / Y / PY / PN / N / NI |
|  | **Risk of bias judgement** | Critical | Low / Moderate / Serious / Critical / NI |
|  | Optional: What is the predicted direction of bias due to confounding? |  | Favours experimental / Favours comparator / Unpredictable |

| **Bias in selection of participants into the study** | | | |
| --- | --- | --- | --- |
|  | 2.1. Was selection of participants into the study (or into the analysis) based on participant characteristics observed after the start of intervention?  **If N/PN to 2.1:** go to 2.4 | N | Y / PY / PN / N / NI |
|  | 2.2. **If Y/PY to 2.1**: Were the post-intervention variables that influenced selection likely to be associated with intervention?  2.3 **If Y/PY to 2.2**: Were the post-intervention variables that influenced selection likely to be influenced by the outcome or a cause of the outcome? |  | NA / Y / PY / PN / N / NI  NA / Y / PY / PN / N / NI |
|  | 2.4. Do start of follow-up and start of intervention coincide for most participants? | Y | Y / PY / PN / N / NI |
|  | 2.5. **If Y/PY to 2.2 and 2.3, or N/PN to 2.4**: Were adjustment techniques used that are likely to correct for the presence of selection biases? |  | NA / Y / PY / PN / N / NI |
|  | **Risk of bias judgement** | L | Low / Moderate / Serious / Critical / NI |
|  | Optional: What is the predicted direction of bias due to selection of participants into the study? |  | Favours experimental / Favours comparator / Towards null /Away from null / Unpredictable |

| **Bias in classification of interventions** | | | |
| --- | --- | --- | --- |
|  | 3.1 Were intervention groups clearly defined? | Y – dependent on NH. 8NHs in Colorado vs 8 NH in Kansas and Missouri (control) | Y / PY / PN / N / NI |
|  | 3.2 Was the information used to define intervention groups recorded at the start of the intervention? | Y | Y / PY / PN / N / NI |
|  | 3.3 Could classification of intervention status have been affected by knowledge of the outcome or risk of the outcome? | N | Y / PY / PN / N / NI |
|  | **Risk of bias judgement** | L | Low / Moderate / Serious / Critical / NI |
|  | Optional: What is the predicted direction of bias due to classification of interventions? |  | Favours experimental / Favours comparator / Towards null /Away from null / Unpredictable |

| **Bias due to deviations from intended interventions** | | | |
| --- | --- | --- | --- |
|  | **If your aim for this study is to assess the effect of assignment to intervention, answer questions 4.1 and 4.2** | |  |
|  | 4.1. Were there deviations from the intended intervention beyond what would be expected in usual practice? | N | Y / PY / PN / N / NI |
|  | 4.2. **If Y/PY to 4.1**: Were these deviations from intended intervention unbalanced between groups *and* likely to have affected the outcome? |  | NA / Y / PY / PN / N / NI |
|  | **If your aim for this study is to assess the effect of starting and adhering to intervention, answer questions 4.3 to 4.6** | |  |
|  | 4.3. Were important co-interventions balanced across intervention groups? | N | Y / PY / PN / N / NI |
|  | 4.4. Was the intervention implemented successfully for most participants? | Y | Y / PY / PN / N / NI |
|  | 4.5. Did study participants adhere to the assigned intervention regimen? | PN: adherence rates were low (57→ 75% in intervention facilities and 38 → 31% in control facilities for adherence to within 4 hrs, and 60→66% in intervention facilities, and 32→39% in control facilities for use of recommended AB) [note: adherence change is not significant.] | Y / PY / PN / N / NI |
|  | 4.6. **If N/PN to 4.3, 4.4 or 4.5**: Was an appropriate analysis used to estimate the effect of starting and adhering to the intervention? | N | NA / Y / PY / PN / N / NI |
|  | **Risk of bias judgement** | Serious |  |
|  | Optional: What is the predicted direction of bias due to deviations from the intended interventions? |  |  |

| **Bias due to missing data** | | | |
| --- | --- | --- | --- |
|  | 5.1 Were outcome data available for all, or nearly all, participants? | Y – all relevant data available. | Y / PY / PN / N / NI |
|  | 5.2 Were participants excluded due to missing data on intervention status? | N | Y / PY / PN / N / NI |
|  | 5.3 Were participants excluded due to missing data on other variables needed for the analysis? | N | Y / PY / PN / N / NI |
|  | 5.4 **If PN/N to 5.1, or Y/PY to 5.2 or 5.3**: Are the proportion of participants and reasons for missing data similar across interventions? |  | NA / Y / PY / PN / N / NI |
|  | 5.5 **If PN/N to 5.1, or Y/PY to 5.2 or 5.3**: Is there evidence that results were robust to the presence of missing data? |  | NA / Y / PY / PN / N / NI |
|  | **Risk of bias judgement** | Low | Low / Moderate / Serious / Critical / NI |
|  | Optional: What is the predicted direction of bias due to missing data? |  | Favours experimental / Favours comparator / Towards null /Away from null / Unpredictable |

| **Bias in measurement of outcomes** | | | |
| --- | --- | --- | --- |
|  | 6.1 Could the outcome measure have been influenced by knowledge of the intervention received? | PY: Academic detailing is likely to have confounds, especially on outcome (pressure of being watched etc) | Y / PY / PN / N / NI |
|  | 6.2 Were outcome assessors aware of the intervention received by study participants? | Y: not possible to prevent as intervention team is tagged to treatment in intervention condition | Y / PY / PN / N / NI |
|  | 6.3 Were the methods of outcome assessment comparable across intervention groups? | Y | Y / PY / PN / N / NI |
|  | 6.4 Were any systematic errors in measurement of the outcome related to intervention received? | N | Y / PY / PN / N / NI |
|  | **Risk of bias judgement** | Moderate | Low / Moderate / Serious / Critical / NI |
|  | Optional: What is the predicted direction of bias due to measurement of outcomes? |  | Favours experimental / Favours comparator / Towards null /Away from null / Unpredictable |

| **Bias in selection of the reported result** | | | |
| --- | --- | --- | --- |
|  | Is the reported effect estimate likely to be selected, on the basis of the results, from... |  |  |
|  | 7.1. ... multiple outcome *measurements* within the outcome domain? | N | Y / PY / PN / N / NI |
|  | 7.2 ... multiple *analyses* of the intervention-outcome relationship? | N | Y / PY / PN / N / NI |
|  | 7.3 ... different *subgroups*? | N | Y / PY / PN / N / NI |
|  | **Risk of bias judgement** | Low | Low / Moderate / Serious / Critical / NI |
|  | Optional: What is the predicted direction of bias due to selection of the reported result? |  | Favours experimental / Favours comparator / Towards null /Away from null / Unpredictable |

| **Overall bias** | | | |
| --- | --- | --- | --- |
|  | **Risk of bias judgement** | Critical | Low / Moderate / Serious / Critical / NI |
|  | Optional: What is the overall predicted direction of bias for this outcome? |  | Favours experimental / Favours comparator / Towards null /Away from null / Unpredictable |

**McMaughen et al 2016**

Responses underlined in green are potential markers for low risk of bias, and responses in red are potential markers for a risk of bias. Where questions relate only to sign posts to other questions, no formatting is used.

|  | **Signalling questions** | **Description** | **Response options** |
| --- | --- | --- | --- |
| **Bias due to confounding** | | | |
|  | 1.1 Is there potential for confounding of the effect of intervention in this study?  **If N/PN to 1.1:** the study can be considered to be at low risk of bias due to confounding and no further signalling questions need be considered | Y | Y / PY / PN / N |
|  | **If Y/PY to 1.1**: determine whether there is a need to assess time-varying confounding: |  |  |
|  | 1.2. Was the analysis based on splitting participants’ follow up time according to intervention received?  **If N/PN**, answer questions relating to baseline confounding (1.4 to 1.6)  **If Y/PY**, go to question 1.3. | N | NA / Y / PY / PN / N / NI |
|  | 1.3. Were intervention discontinuations or switches likely to be related to factors that are prognostic for the outcome?  **If N/PN**, answer questions relating to baseline confounding (1.4 to 1.6)  **If Y/PY**, answer questions relating to both baseline and time-varying confounding (1.7 and 1.8) | N | NA / Y / PY / PN / N / NI |

|  | **Questions relating to baseline confounding only** | | |
| --- | --- | --- | --- |
|  | 1.4. Did the authors use an appropriate analysis method that controlled for all the important confounding domains? | PY; use of ANOVA for a 2x2 calculation | NA / Y / PY / PN / N / NI |
|  | 1.5. **If Y/PY to 1.4**: Were confounding domains that were controlled for measured validly and reliably by the variables available in this study? | PY | NA / Y / PY / PN / N / NI |
|  | 1.6. Did the authors control for any post-intervention variables that could have been affected by the intervention? | PY; fidelity was taken into consideration during calculation – see later section. | NA / Y / PY / PN / N / NI |
|  | **Questions relating to baseline and time-varying confounding** | |  |
|  | 1.7. Did the authors use an appropriate analysis method that controlled for all the important confounding domains and for time-varying confounding? |  | NA / Y / PY / PN / N / NI |
|  | 1.8. **If Y/PY to 1.7**: Were confounding domains that were controlled for measured validly and reliably by the variables available in this study? |  | NA / Y / PY / PN / N / NI |
|  | **Risk of bias judgement** | Moderate | Low / Moderate / Serious / Critical / NI |
|  | Optional: What is the predicted direction of bias due to confounding? |  | Favours experimental / Favours comparator / Unpredictable |

| **Bias in selection of participants into the study** | | | |
| --- | --- | --- | --- |
|  | 2.1. Was selection of participants into the study (or into the analysis) based on participant characteristics observed after the start of intervention?  **If N/PN to 2.1:** go to 2.4 | N | Y / PY / PN / N / NI |
|  | 2.2. **If Y/PY to 2.1**: Were the post-intervention variables that influenced selection likely to be associated with intervention?  2.3 **If Y/PY to 2.2**: Were the post-intervention variables that influenced selection likely to be influenced by the outcome or a cause of the outcome? |  | NA / Y / PY / PN / N / NI  NA / Y / PY / PN / N / NI |
|  | 2.4. Do start of follow-up and start of intervention coincide for most participants? | Y | Y / PY / PN / N / NI |
|  | 2.5. **If Y/PY to 2.2 and 2.3, or N/PN to 2.4**: Were adjustment techniques used that are likely to correct for the presence of selection biases? |  | NA / Y / PY / PN / N / NI |
|  | **Risk of bias judgement** | Low | Low / Moderate / Serious / Critical / NI |
|  | Optional: What is the predicted direction of bias due to selection of participants into the study? |  | Favours experimental / Favours comparator / Towards null /Away from null / Unpredictable |

| **Bias in classification of interventions** | | | |
| --- | --- | --- | --- |
|  | 3.1 Were intervention groups clearly defined? | Y – Pre/Post test analysis with comparison group. | Y / PY / PN / N / NI |
|  | 3.2 Was the information used to define intervention groups recorded at the start of the intervention? | Y | Y / PY / PN / N / NI |
|  | 3.3 Could classification of intervention status have been affected by knowledge of the outcome or risk of the outcome? | N | Y / PY / PN / N / NI |
|  | **Risk of bias judgement** | Low | Low / Moderate / Serious / Critical / NI |
|  | Optional: What is the predicted direction of bias due to classification of interventions? |  | Favours experimental / Favours comparator / Towards null /Away from null / Unpredictable |

| **Bias due to deviations from intended interventions** | | | |
| --- | --- | --- | --- |
|  | **If your aim for this study is to assess the effect of assignment to intervention, answer questions 4.1 and 4.2** | |  |
|  | 4.1. Were there deviations from the intended intervention beyond what would be expected in usual practice? | N | Y / PY / PN / N / NI |
|  | 4.2. **If Y/PY to 4.1**: Were these deviations from intended intervention unbalanced between groups *and* likely to have affected the outcome? |  | NA / Y / PY / PN / N / NI |
|  | **If your aim for this study is to assess the effect of starting and adhering to intervention, answer questions 4.3 to 4.6** | |  |
|  | 4.3. Were important co-interventions balanced across intervention groups? | Y – provision of decision-making aid to all NHs, under 3 conditions – control (no training, only when NH requests), low intensity training, and high intensity training. | Y / PY / PN / N / NI |
|  | 4.4. Was the intervention implemented successfully for most participants? | Y – intervention involves the use of tool. | Y / PY / PN / N / NI |
|  | 4.5. Did study participants adhere to the assigned intervention regimen? | N; fidelity was an issue; 4 nursing home in the intervention condition (1 in high intensity, 3 in low intensity) had low fidelity (<25% implementation) | Y / PY / PN / N / NI |
|  | 4.6. **If N/PN to 4.3, 4.4 or 4.5**: Was an appropriate analysis used to estimate the effect of starting and adhering to the intervention? | Y | NA / Y / PY / PN / N / NI |
|  | **Risk of bias judgement** | Moderate |  |
|  | Optional: What is the predicted direction of bias due to deviations from the intended interventions? |  |  |

| **Bias due to missing data** | | | |
| --- | --- | --- | --- |
|  | 5.1 Were outcome data available for all, or nearly all, participants? | PY | Y / PY / PN / N / NI |
|  | 5.2 Were participants excluded due to missing data on intervention status? | N | Y / PY / PN / N / NI |
|  | 5.3 Were participants excluded due to missing data on other variables needed for the analysis? | N | Y / PY / PN / N / NI |
|  | 5.4 **If PN/N to 5.1, or Y/PY to 5.2 or 5.3**: Are the proportion of participants and reasons for missing data similar across interventions? |  | NA / Y / PY / PN / N / NI |
|  | 5.5 **If PN/N to 5.1, or Y/PY to 5.2 or 5.3**: Is there evidence that results were robust to the presence of missing data? |  | NA / Y / PY / PN / N / NI |
|  | **Risk of bias judgement** | Low | Low / Moderate / Serious / Critical / NI |
|  | Optional: What is the predicted direction of bias due to missing data? |  | Favours experimental / Favours comparator / Towards null /Away from null / Unpredictable |

| **Bias in measurement of outcomes** | | | |
| --- | --- | --- | --- |
|  | 6.1 Could the outcome measure have been influenced by knowledge of the intervention received? | PN: use of decision making aid meant that it unlikely that the intervention has too much impact as physicians were trained to look for particular symptoms/features of infection | Y / PY / PN / N / NI |
|  | 6.2 Were outcome assessors aware of the intervention received by study participants? | Y; however, unlikely to have impact as physicians are following the decision-making aid. Moreover, collection was done by a clinically trained nurse – it is unclear whether she was blinded. | Y / PY / PN / N / NI |
|  | 6.3 Were the methods of outcome assessment comparable across intervention groups? | Y | Y / PY / PN / N / NI |
|  | 6.4 Were any systematic errors in measurement of the outcome related to intervention received? | N | Y / PY / PN / N / NI |
|  | **Risk of bias judgement** | Moderate | Low / Moderate / Serious / Critical / NI |
|  | Optional: What is the predicted direction of bias due to measurement of outcomes? |  | Favours experimental / Favours comparator / Towards null /Away from null / Unpredictable |

| **Bias in selection of the reported result** | | | |
| --- | --- | --- | --- |
|  | Is the reported effect estimate likely to be selected, on the basis of the results, from... |  |  |
|  | 7.1. ... multiple outcome *measurements* within the outcome domain? | N | Y / PY / PN / N / NI |
|  | 7.2 ... multiple *analyses* of the intervention-outcome relationship? | PY; initial outcome was not significant, likely due to fidelity issues. However, paper analyzed performed regression analysis taking into account of fidelity/no fidelity conditions instead, finding a significant relationship and publishing results. | Y / PY / PN / N / NI |
|  | 7.3 ... different *subgroups*? | N | Y / PY / PN / N / NI |
|  | **Risk of bias judgement** | Serious | Low / Moderate / Serious / Critical / NI |
|  | Optional: What is the predicted direction of bias due to selection of the reported result? |  | Favours experimental / Favours comparator / Towards null /Away from null / Unpredictable |

| **Overall bias** | | | |
| --- | --- | --- | --- |
|  | **Risk of bias judgement** | Serious | Low / Moderate / Serious / Critical / NI |
|  | Optional: What is the overall predicted direction of bias for this outcome? |  | Favours experimental / Favours comparator / Towards null /Away from null / Unpredictable |

**Rehme et al 2016**

Responses underlined in green are potential markers for low risk of bias, and responses in red are potential markers for a risk of bias. Where questions relate only to sign posts to other questions, no formatting is used.

|  | **Signalling questions** | **Description** | **Response options** |
| --- | --- | --- | --- |
| **Bias due to confounding** | | | |
|  | 1.1 Is there potential for confounding of the effect of intervention in this study?  **If N/PN to 1.1:** the study can be considered to be at low risk of bias due to confounding and no further signalling questions need be considered | PN | Y / PY / PN / N |
|  | **If Y/PY to 1.1**: determine whether there is a need to assess time-varying confounding: |  |  |
|  | 1.2. Was the analysis based on splitting participants’ follow up time according to intervention received?  **If N/PN**, answer questions relating to baseline confounding (1.4 to 1.6)  **If Y/PY**, go to question 1.3. |  | NA / Y / PY / PN / N / NI |
|  | 1.3. Were intervention discontinuations or switches likely to be related to factors that are prognostic for the outcome?  **If N/PN**, answer questions relating to baseline confounding (1.4 to 1.6)  **If Y/PY**, answer questions relating to both baseline and time-varying confounding (1.7 and 1.8) |  | NA / Y / PY / PN / N / NI |

|  | **Questions relating to baseline confounding only** | | |
| --- | --- | --- | --- |
|  | 1.4. Did the authors use an appropriate analysis method that controlled for all the important confounding domains? | Y. Use of SPSS to perform analysis. | NA / Y / PY / PN / N / NI |
|  | 1.5. **If Y/PY to 1.4**: Were confounding domains that were controlled for measured validly and reliably by the variables available in this study? | PY | NA / Y / PY / PN / N / NI |
|  | 1.6. Did the authors control for any post-intervention variables that could have been affected by the intervention? | N | NA / Y / PY / PN / N / NI |
|  | **Questions relating to baseline and time-varying confounding** | |  |
|  | 1.7. Did the authors use an appropriate analysis method that controlled for all the important confounding domains and for time-varying confounding? |  | NA / Y / PY / PN / N / NI |
|  | 1.8. **If Y/PY to 1.7**: Were confounding domains that were controlled for measured validly and reliably by the variables available in this study? |  | NA / Y / PY / PN / N / NI |
|  | **Risk of bias judgement** | Low | Low / Moderate / Serious / Critical / NI |
|  | Optional: What is the predicted direction of bias due to confounding? |  | Favours experimental / Favours comparator / Unpredictable |

| **Bias in selection of participants into the study** | | | |
| --- | --- | --- | --- |
|  | 2.1. Was selection of participants into the study (or into the analysis) based on participant characteristics observed after the start of intervention?  **If N/PN to 2.1:** go to 2.4 | N | Y / PY / PN / N / NI |
|  | 2.2. **If Y/PY to 2.1**: Were the post-intervention variables that influenced selection likely to be associated with intervention?  2.3 **If Y/PY to 2.2**: Were the post-intervention variables that influenced selection likely to be influenced by the outcome or a cause of the outcome? |  | NA / Y / PY / PN / N / NI  NA / Y / PY / PN / N / NI |
|  | 2.4. Do start of follow-up and start of intervention coincide for most participants? | Y | Y / PY / PN / N / NI |
|  | 2.5. **If Y/PY to 2.2 and 2.3, or N/PN to 2.4**: Were adjustment techniques used that are likely to correct for the presence of selection biases? |  | NA / Y / PY / PN / N / NI |
|  | **Risk of bias judgement** | Low | Low / Moderate / Serious / Critical / NI |
|  | Optional: What is the predicted direction of bias due to selection of participants into the study? |  | Favours experimental / Favours comparator / Towards null /Away from null / Unpredictable |

| **Bias in classification of interventions** | | | |
| --- | --- | --- | --- |
|  | 3.1 Were intervention groups clearly defined? | Y – staff in a local LTCF. Pre/post intervention design. | Y / PY / PN / N / NI |
|  | 3.2 Was the information used to define intervention groups recorded at the start of the intervention? | Y | Y / PY / PN / N / NI |
|  | 3.3 Could classification of intervention status have been affected by knowledge of the outcome or risk of the outcome? | N | Y / PY / PN / N / NI |
|  | **Risk of bias judgement** | Low | Low / Moderate / Serious / Critical / NI |
|  | Optional: What is the predicted direction of bias due to classification of interventions? |  | Favours experimental / Favours comparator / Towards null /Away from null / Unpredictable |

| **Bias due to deviations from intended interventions** | | | |
| --- | --- | --- | --- |
|  | **If your aim for this study is to assess the effect of assignment to intervention, answer questions 4.1 and 4.2** | |  |
|  | 4.1. Were there deviations from the intended intervention beyond what would be expected in usual practice? | N | Y / PY / PN / N / NI |
|  | 4.2. **If Y/PY to 4.1**: Were these deviations from intended intervention unbalanced between groups *and* likely to have affected the outcome? |  | NA / Y / PY / PN / N / NI |
|  | **If your aim for this study is to assess the effect of starting and adhering to intervention, answer questions 4.3 to 4.6** | |  |
|  | 4.3. Were important co-interventions balanced across intervention groups? | NA; not possible to implement co-intervention as intervention focused on education and better communication – only one intervention function tested | Y / PY / PN / N / NI |
|  | 4.4. Was the intervention implemented successfully for most participants? | Y | Y / PY / PN / N / NI |
|  | 4.5. Did study participants adhere to the assigned intervention regimen? | NI | Y / PY / PN / N / NI |
|  | 4.6. **If N/PN to 4.3, 4.4 or 4.5**: Was an appropriate analysis used to estimate the effect of starting and adhering to the intervention? |  | NA / Y / PY / PN / N / NI |
|  | **Risk of bias judgement** | Low |  |
|  | Optional: What is the predicted direction of bias due to deviations from the intended interventions? |  |  |

| **Bias due to missing data** | | | |
| --- | --- | --- | --- |
|  | 5.1 Were outcome data available for all, or nearly all, participants? | Y – all relevant data available. | Y / PY / PN / N / NI |
|  | 5.2 Were participants excluded due to missing data on intervention status? | N | Y / PY / PN / N / NI |
|  | 5.3 Were participants excluded due to missing data on other variables needed for the analysis? | N | Y / PY / PN / N / NI |
|  | 5.4 **If PN/N to 5.1, or Y/PY to 5.2 or 5.3**: Are the proportion of participants and reasons for missing data similar across interventions? |  | NA / Y / PY / PN / N / NI |
|  | 5.5 **If PN/N to 5.1, or Y/PY to 5.2 or 5.3**: Is there evidence that results were robust to the presence of missing data? |  | NA / Y / PY / PN / N / NI |
|  | **Risk of bias judgement** | Low | Low / Moderate / Serious / Critical / NI |
|  | Optional: What is the predicted direction of bias due to missing data? |  | Favours experimental / Favours comparator / Towards null /Away from null / Unpredictable |

| **Bias in measurement of outcomes** | | | |
| --- | --- | --- | --- |
|  | 6.1 Could the outcome measure have been influenced by knowledge of the intervention received? | PN: AB data unlikely to be affected by knowledge of communications channel - any change is likely due to better communication and collaboration. | Y / PY / PN / N / NI |
|  | 6.2 Were outcome assessors aware of the intervention received by study participants? | Y; education and change of communication lines is obvious. | Y / PY / PN / N / NI |
|  | 6.3 Were the methods of outcome assessment comparable across intervention groups? | Y | Y / PY / PN / N / NI |
|  | 6.4 Were any systematic errors in measurement of the outcome related to intervention received? | N | Y / PY / PN / N / NI |
|  | **Risk of bias judgement** | Low | Low / Moderate / Serious / Critical / NI |
|  | Optional: What is the predicted direction of bias due to measurement of outcomes? |  | Favours experimental / Favours comparator / Towards null /Away from null / Unpredictable |

| **Bias in selection of the reported result** | | | |
| --- | --- | --- | --- |
|  | Is the reported effect estimate likely to be selected, on the basis of the results, from... |  |  |
|  | 7.1. ... multiple outcome *measurements* within the outcome domain? | N | Y / PY / PN / N / NI |
|  | 7.2 ... multiple *analyses* of the intervention-outcome relationship? | N | Y / PY / PN / N / NI |
|  | 7.3 ... different *subgroups*? | N | Y / PY / PN / N / NI |
|  | **Risk of bias judgement** | Low | Low / Moderate / Serious / Critical / NI |
|  | Optional: What is the predicted direction of bias due to selection of the reported result? |  | Favours experimental / Favours comparator / Towards null /Away from null / Unpredictable |

| **Overall bias** | | | |
| --- | --- | --- | --- |
|  | **Risk of bias judgement** | Low | Low / Moderate / Serious / Critical / NI |
|  | Optional: What is the overall predicted direction of bias for this outcome? |  | Favours experimental / Favours comparator / Towards null /Away from null / Unpredictable |

**Schwartz et al 2007**

Responses underlined in green are potential markers for low risk of bias, and responses in red are potential markers for a risk of bias. Where questions relate only to sign posts to other questions, no formatting is used.

|  | **Signalling questions** | **Description** | **Response options** |
| --- | --- | --- | --- |
| **Bias due to confounding** | | | |
|  | 1.1 Is there potential for confounding of the effect of intervention in this study?  **If N/PN to 1.1:** the study can be considered to be at low risk of bias due to confounding and no further signalling questions need be considered | Y | Y / PY / PN / N |
|  | **If Y/PY to 1.1**: determine whether there is a need to assess time-varying confounding: |  |  |
|  | 1.2. Was the analysis based on splitting participants’ follow up time according to intervention received?  **If N/PN**, answer questions relating to baseline confounding (1.4 to 1.6)  **If Y/PY**, go to question 1.3. | N | NA / Y / PY / PN / N / NI |
|  | 1.3. Were intervention discontinuations or switches likely to be related to factors that are prognostic for the outcome?  **If N/PN**, answer questions relating to baseline confounding (1.4 to 1.6)  **If Y/PY**, answer questions relating to both baseline and time-varying confounding (1.7 and 1.8) |  | NA / Y / PY / PN / N / NI |

|  | **Questions relating to baseline confounding only** | | |
| --- | --- | --- | --- |
|  | 1.4. Did the authors use an appropriate analysis method that controlled for all the important confounding domains? | PY; serial adjustment and time-series analysis, adjusted with ARIMA models | NA / Y / PY / PN / N / NI |
|  | 1.5. **If Y/PY to 1.4**: Were confounding domains that were controlled for measured validly and reliably by the variables available in this study? | Y | NA / Y / PY / PN / N / NI |
|  | 1.6. Did the authors control for any post-intervention variables that could have been affected by the intervention? |  | NA / Y / PY / PN / N / NI |
|  | **Questions relating to baseline and time-varying confounding** | |  |
|  | 1.7. Did the authors use an appropriate analysis method that controlled for all the important confounding domains and for time-varying confounding? |  | NA / Y / PY / PN / N / NI |
|  | 1.8. **If Y/PY to 1.7**: Were confounding domains that were controlled for measured validly and reliably by the variables available in this study? |  | NA / Y / PY / PN / N / NI |
|  | **Risk of bias judgement** | Low | Low / Moderate / Serious / Critical / NI |
|  | Optional: What is the predicted direction of bias due to confounding? |  | Favours experimental / Favours comparator / Unpredictable |

| **Bias in selection of participants into the study** | | | |
| --- | --- | --- | --- |
|  | 2.1. Was selection of participants into the study (or into the analysis) based on participant characteristics observed after the start of intervention?  **If N/PN to 2.1:** go to 2.4 | N | Y / PY / PN / N / NI |
|  | 2.2. **If Y/PY to 2.1**: Were the post-intervention variables that influenced selection likely to be associated with intervention?  2.3 **If Y/PY to 2.2**: Were the post-intervention variables that influenced selection likely to be influenced by the outcome or a cause of the outcome? |  | NA / Y / PY / PN / N / NI  NA / Y / PY / PN / N / NI |
|  | 2.4. Do start of follow-up and start of intervention coincide for most participants? | Y | Y / PY / PN / N / NI |
|  | 2.5. **If Y/PY to 2.2 and 2.3, or N/PN to 2.4**: Were adjustment techniques used that are likely to correct for the presence of selection biases? |  | NA / Y / PY / PN / N / NI |
|  | **Risk of bias judgement** | Low | Low / Moderate / Serious / Critical / NI |
|  | Optional: What is the predicted direction of bias due to selection of participants into the study? |  | Favours experimental / Favours comparator / Towards null /Away from null / Unpredictable |

| **Bias in classification of interventions** | | | |
| --- | --- | --- | --- |
|  | 3.1 Were intervention groups clearly defined? | Y – 20 salaried internists | Y / PY / PN / N / NI |
|  | 3.2 Was the information used to define intervention groups recorded at the start of the intervention? | Y | Y / PY / PN / N / NI |
|  | 3.3 Could classification of intervention status have been affected by knowledge of the outcome or risk of the outcome? | N | Y / PY / PN / N / NI |
|  | **Risk of bias judgement** | Low | Low / Moderate / Serious / Critical / NI |
|  | Optional: What is the predicted direction of bias due to classification of interventions? |  | Favours experimental / Favours comparator / Towards null /Away from null / Unpredictable |

| **Bias due to deviations from intended interventions** | | | |
| --- | --- | --- | --- |
|  | **If your aim for this study is to assess the effect of assignment to intervention, answer questions 4.1 and 4.2** | |  |
|  | 4.1. Were there deviations from the intended intervention beyond what would be expected in usual practice? | PN; slightly unconventional use of measurements (number of starts), but complemented with amount of AB use similar to DDDs | Y / PY / PN / N / NI |
|  | 4.2. **If Y/PY to 4.1**: Were these deviations from intended intervention unbalanced between groups *and* likely to have affected the outcome? |  | NA / Y / PY / PN / N / NI |
|  | **If your aim for this study is to assess the effect of starting and adhering to intervention, answer questions 4.3 to 4.6** | |  |
|  | 4.3. Were important co-interventions balanced across intervention groups? | Na; no other important co-intervention. Intervention incorporated ‘national guidelines, hospital resistance data, and physician feedback…series of four teaching sessions over 18 months and booklets detailing institutional guidelines’. | Y / PY / PN / N / NI |
|  | 4.4. Was the intervention implemented successfully for most participants? | Y | Y / PY / PN / N / NI |
|  | 4.5. Did study participants adhere to the assigned intervention regimen? | Y | Y / PY / PN / N / NI |
|  | 4.6. **If N/PN to 4.3, 4.4 or 4.5**: Was an appropriate analysis used to estimate the effect of starting and adhering to the intervention? |  | NA / Y / PY / PN / N / NI |
|  | **Risk of bias judgement** | Low |  |
|  | Optional: What is the predicted direction of bias due to deviations from the intended interventions? |  |  |

| **Bias due to missing data** | | | |
| --- | --- | --- | --- |
|  | 5.1 Were outcome data available for all, or nearly all, participants? | Y | Y / PY / PN / N / NI |
|  | 5.2 Were participants excluded due to missing data on intervention status? | N | Y / PY / PN / N / NI |
|  | 5.3 Were participants excluded due to missing data on other variables needed for the analysis? | N | Y / PY / PN / N / NI |
|  | 5.4 **If PN/N to 5.1, or Y/PY to 5.2 or 5.3**: Are the proportion of participants and reasons for missing data similar across interventions? |  | NA / Y / PY / PN / N / NI |
|  | 5.5 **If PN/N to 5.1, or Y/PY to 5.2 or 5.3**: Is there evidence that results were robust to the presence of missing data? |  | NA / Y / PY / PN / N / NI |
|  | **Risk of bias judgement** | Low | Low / Moderate / Serious / Critical / NI |
|  | Optional: What is the predicted direction of bias due to missing data? |  | Favours experimental / Favours comparator / Towards null /Away from null / Unpredictable |

| **Bias in measurement of outcomes** | | | |
| --- | --- | --- | --- |
|  | 6.1 Could the outcome measure have been influenced by knowledge of the intervention received? | PY – it is possible, even if unlikely. Not possible to hide intervention from participants as it is an educational intervention | Y / PY / PN / N / NI |
|  | 6.2 Were outcome assessors aware of the intervention received by study participants? | Y; study characteristics (pre/post). Implementation of educational intervention meant that the assessors were aware of who the participants were | Y / PY / PN / N / NI |
|  | 6.3 Were the methods of outcome assessment comparable across intervention groups? | Y | Y / PY / PN / N / NI |
|  | 6.4 Were any systematic errors in measurement of the outcome related to intervention received? | PY, but effect likely to be small. Implementation of electronic pharmaceutical data in Jan 2000. Prescriptions from Dec were added to Jan 1 and 2 thus discounted. | Y / PY / PN / N / NI |
|  | **Risk of bias judgement** | Moderate | Low / Moderate / Serious / Critical / NI |
|  | Optional: What is the predicted direction of bias due to measurement of outcomes? |  | Favours experimental / Favours comparator / Towards null /Away from null / Unpredictable |

| **Bias in selection of the reported result** | | | |
| --- | --- | --- | --- |
|  | Is the reported effect estimate likely to be selected, on the basis of the results, from... |  |  |
|  | 7.1. ... multiple outcome *measurements* within the outcome domain? | N | Y / PY / PN / N / NI |
|  | 7.2 ... multiple *analyses* of the intervention-outcome relationship? | N | Y / PY / PN / N / NI |
|  | 7.3 ... different *subgroups*? | N | Y / PY / PN / N / NI |
|  | **Risk of bias judgement** | Low | Low / Moderate / Serious / Critical / NI |
|  | Optional: What is the predicted direction of bias due to selection of the reported result? |  | Favours experimental / Favours comparator / Towards null /Away from null / Unpredictable |

| **Overall bias** | | | |
| --- | --- | --- | --- |
|  | **Risk of bias judgement** | Moderate | Low / Moderate / Serious / Critical / NI |
|  | Optional: What is the overall predicted direction of bias for this outcome? |  | Favours experimental / Favours comparator / Towards null /Away from null / Unpredictable |

**Smith et al 2016**

Responses underlined in green are potential markers for low risk of bias, and responses in red are potential markers for a risk of bias. Where questions relate only to sign posts to other questions, no formatting is used.

|  | **Signalling questions** | **Description** | **Response options** |
| --- | --- | --- | --- |
| **Bias due to confounding** | | | |
|  | 1.1 Is there potential for confounding of the effect of intervention in this study?  **If N/PN to 1.1:** the study can be considered to be at low risk of bias due to confounding and no further signalling questions need be considered | Y | Y / PY / PN / N |
|  | **If Y/PY to 1.1**: determine whether there is a need to assess time-varying confounding: | N |  |
|  | 1.2. Was the analysis based on splitting participants’ follow up time according to intervention received?  **If N/PN**, answer questions relating to baseline confounding (1.4 to 1.6)  **If Y/PY**, go to question 1.3. |  | NA / Y / PY / PN / N / NI |
|  | 1.3. Were intervention discontinuations or switches likely to be related to factors that are prognostic for the outcome?  **If N/PN**, answer questions relating to baseline confounding (1.4 to 1.6)  **If Y/PY**, answer questions relating to both baseline and time-varying confounding (1.7 and 1.8) |  | NA / Y / PY / PN / N / NI |

|  | **Questions relating to baseline confounding only** | | |
| --- | --- | --- | --- |
|  | 1.4. Did the authors use an appropriate analysis method that controlled for all the important confounding domains? | Y; patient characteristics not significantly different | NA / Y / PY / PN / N / NI |
|  | 1.5. **If Y/PY to 1.4**: Were confounding domains that were controlled for measured validly and reliably by the variables available in this study? | PY | NA / Y / PY / PN / N / NI |
|  | 1.6. Did the authors control for any post-intervention variables that could have been affected by the intervention? | N | NA / Y / PY / PN / N / NI |
|  | **Questions relating to baseline and time-varying confounding** | |  |
|  | 1.7. Did the authors use an appropriate analysis method that controlled for all the important confounding domains and for time-varying confounding? |  | NA / Y / PY / PN / N / NI |
|  | 1.8. **If Y/PY to 1.7**: Were confounding domains that were controlled for measured validly and reliably by the variables available in this study? |  | NA / Y / PY / PN / N / NI |
|  | **Risk of bias judgement** | Low | Low / Moderate / Serious / Critical / NI |
|  | Optional: What is the predicted direction of bias due to confounding? |  | Favours experimental / Favours comparator / Unpredictable |

| **Bias in selection of participants into the study** | | | |
| --- | --- | --- | --- |
|  | 2.1. Was selection of participants into the study (or into the analysis) based on participant characteristics observed after the start of intervention?  **If N/PN to 2.1:** go to 2.4 | N | Y / PY / PN / N / NI |
|  | 2.2. **If Y/PY to 2.1**: Were the post-intervention variables that influenced selection likely to be associated with intervention?  2.3 **If Y/PY to 2.2**: Were the post-intervention variables that influenced selection likely to be influenced by the outcome or a cause of the outcome? |  | NA / Y / PY / PN / N / NI  NA / Y / PY / PN / N / NI |
|  | 2.4. Do start of follow-up and start of intervention coincide for most participants? | Y | Y / PY / PN / N / NI |
|  | 2.5. **If Y/PY to 2.2 and 2.3, or N/PN to 2.4**: Were adjustment techniques used that are likely to correct for the presence of selection biases? |  | NA / Y / PY / PN / N / NI |
|  | **Risk of bias judgement** | Low | Low / Moderate / Serious / Critical / NI |
|  | Optional: What is the predicted direction of bias due to selection of participants into the study? |  | Favours experimental / Favours comparator / Towards null /Away from null / Unpredictable |

| **Bias in classification of interventions** | | | |
| --- | --- | --- | --- |
|  | 3.1 Were intervention groups clearly defined? | Y – patients in NH after certain date (implementation of intervention), and fit the following characteristics: >18 years, received ≥4 doses of vancomycin, creatinine clearance ≥ 15 mL/min, not receiving renal replacement therapy. | Y / PY / PN / N / NI |
|  | 3.2 Was the information used to define intervention groups recorded at the start of the intervention? | Y | Y / PY / PN / N / NI |
|  | 3.3 Could classification of intervention status have been affected by knowledge of the outcome or risk of the outcome? | N | Y / PY / PN / N / NI |
|  | **Risk of bias judgement** | Low | Low / Moderate / Serious / Critical / NI |
|  | Optional: What is the predicted direction of bias due to classification of interventions? |  | Favours experimental / Favours comparator / Towards null /Away from null / Unpredictable |

| **Bias due to deviations from intended interventions** | | | |
| --- | --- | --- | --- |
|  | **If your aim for this study is to assess the effect of assignment to intervention, answer questions 4.1 and 4.2** | |  |
|  | 4.1. Were there deviations from the intended intervention beyond what would be expected in usual practice? | N | Y / PY / PN / N / NI |
|  | 4.2. **If Y/PY to 4.1**: Were these deviations from intended intervention unbalanced between groups *and* likely to have affected the outcome? |  | NA / Y / PY / PN / N / NI |
|  | **If your aim for this study is to assess the effect of starting and adhering to intervention, answer questions 4.3 to 4.6** | |  |
|  | 4.3. Were important co-interventions balanced across intervention groups? | PY; measurements taken pre-intervention for a year as control – in Phase 1, physicians ordered lab tests for vancomycin monitoring; in Phase 2, pharmacists independently ordered vancomycin trough levels, SCr, and BUN. No other co-intervention needed | Y / PY / PN / N / NI |
|  | 4.4. Was the intervention implemented successfully for most participants? | Y; improvement in adherence from 75% to 90+% as well. | Y / PY / PN / N / NI |
|  | 4.5. Did study participants adhere to the assigned intervention regimen? | Y | Y / PY / PN / N / NI |
|  | 4.6. **If N/PN to 4.3, 4.4 or 4.5**: Was an appropriate analysis used to estimate the effect of starting and adhering to the intervention? |  | NA / Y / PY / PN / N / NI |
|  | **Risk of bias judgement** | Low |  |
|  | Optional: What is the predicted direction of bias due to deviations from the intended interventions? |  |  |

| **Bias due to missing data** | | | |
| --- | --- | --- | --- |
|  | 5.1 Were outcome data available for all, or nearly all, participants? | PY – in Phase 1, 49/174 patients qualified for consideration by patient; in phase 2, 149/359 patients qualified.  → determination of AKI, thus by strict criteria. | Y / PY / PN / N / NI |
|  | 5.2 Were participants excluded due to missing data on intervention status? | N | Y / PY / PN / N / NI |
|  | 5.3 Were participants excluded due to missing data on other variables needed for the analysis? | N | Y / PY / PN / N / NI |
|  | 5.4 **If PN/N to 5.1, or Y/PY to 5.2 or 5.3**: Are the proportion of participants and reasons for missing data similar across interventions? |  | NA / Y / PY / PN / N / NI |
|  | 5.5 **If PN/N to 5.1, or Y/PY to 5.2 or 5.3**: Is there evidence that results were robust to the presence of missing data? |  | NA / Y / PY / PN / N / NI |
|  | **Risk of bias judgement** | Low | Low / Moderate / Serious / Critical / NI |
|  | Optional: What is the predicted direction of bias due to missing data? |  | Favours experimental / Favours comparator / Towards null /Away from null / Unpredictable |

| **Bias in measurement of outcomes** | | | |
| --- | --- | --- | --- |
|  | 6.1 Could the outcome measure have been influenced by knowledge of the intervention received? | N | Y / PY / PN / N / NI |
|  | 6.2 Were outcome assessors aware of the intervention received by study participants? | Y; separated by time, but assessor could not influence outcome – biological measurements. | Y / PY / PN / N / NI |
|  | 6.3 Were the methods of outcome assessment comparable across intervention groups? | Y | Y / PY / PN / N / NI |
|  | 6.4 Were any systematic errors in measurement of the outcome related to intervention received? | N | Y / PY / PN / N / NI |
|  | **Risk of bias judgement** | Low | Low / Moderate / Serious / Critical / NI |
|  | Optional: What is the predicted direction of bias due to measurement of outcomes? |  | Favours experimental / Favours comparator / Towards null /Away from null / Unpredictable |

| **Bias in selection of the reported result** | | | |
| --- | --- | --- | --- |
|  | Is the reported effect estimate likely to be selected, on the basis of the results, from... |  |  |
|  | 7.1. ... multiple outcome *measurements* within the outcome domain? | N | Y / PY / PN / N / NI |
|  | 7.2 ... multiple *analyses* of the intervention-outcome relationship? | N | Y / PY / PN / N / NI |
|  | 7.3 ... different *subgroups*? | N | Y / PY / PN / N / NI |
|  | **Risk of bias judgement** | Low | Low / Moderate / Serious / Critical / NI |
|  | Optional: What is the predicted direction of bias due to selection of the reported result? |  | Favours experimental / Favours comparator / Towards null /Away from null / Unpredictable |

| **Overall bias** | | | |
| --- | --- | --- | --- |
|  | **Risk of bias judgement** | Low | Low / Moderate / Serious / Critical / NI |
|  | Optional: What is the overall predicted direction of bias for this outcome? |  | Favours experimental / Favours comparator / Towards null /Away from null / Unpredictable |

**van Buul et al 2015**

Responses underlined in green are potential markers for low risk of bias, and responses in red are potential markers for a risk of bias. Where questions relate only to sign posts to other questions, no formatting is used.

|  | **Signalling questions** | **Description** | **Response options** |
| --- | --- | --- | --- |
| **Bias due to confounding** | | | |
|  | 1.1 Is there potential for confounding of the effect of intervention in this study?  **If N/PN to 1.1:** the study can be considered to be at low risk of bias due to confounding and no further signalling questions need be considered | Y | Y / PY / PN / N |
|  | **If Y/PY to 1.1**: determine whether there is a need to assess time-varying confounding: |  |  |
|  | 1.2. Was the analysis based on splitting participants’ follow up time according to intervention received?  **If N/PN**, answer questions relating to baseline confounding (1.4 to 1.6)  **If Y/PY**, go to question 1.3. | N | NA / Y / PY / PN / N / NI |
|  | 1.3. Were intervention discontinuations or switches likely to be related to factors that are prognostic for the outcome?  **If N/PN**, answer questions relating to baseline confounding (1.4 to 1.6)  **If Y/PY**, answer questions relating to both baseline and time-varying confounding (1.7 and 1.8) |  | NA / Y / PY / PN / N / NI |

|  | **Questions relating to baseline confounding only** | | |
| --- | --- | --- | --- |
|  | 1.4. Did the authors use an appropriate analysis method that controlled for all the important confounding domains? | N; assignment to intervention/control groups were done nonrandomly. “facilities were allocated to either the intervention or control group…” | NA / Y / PY / PN / N / NI |
|  | 1.5. **If Y/PY to 1.4**: Were confounding domains that were controlled for measured validly and reliably by the variables available in this study? |  | NA / Y / PY / PN / N / NI |
|  | 1.6. Did the authors control for any post-intervention variables that could have been affected by the intervention? | PN; authors accounted for physician turnover which may or may not be related to intervention. | NA / Y / PY / PN / N / NI |
|  | **Questions relating to baseline and time-varying confounding** | |  |
|  | 1.7. Did the authors use an appropriate analysis method that controlled for all the important confounding domains and for time-varying confounding? |  | NA / Y / PY / PN / N / NI |
|  | 1.8. **If Y/PY to 1.7**: Were confounding domains that were controlled for measured validly and reliably by the variables available in this study? |  | NA / Y / PY / PN / N / NI |
|  | **Risk of bias judgement** | Serious | Low / Moderate / Serious / Critical / NI |
|  | Optional: What is the predicted direction of bias due to confounding? |  | Favours experimental / Favours comparator / Unpredictable |

| **Bias in selection of participants into the study** | | | |
| --- | --- | --- | --- |
|  | 2.1. Was selection of participants into the study (or into the analysis) based on participant characteristics observed after the start of intervention?  **If N/PN to 2.1:** go to 2.4 | N | Y / PY / PN / N / NI |
|  | 2.2. **If Y/PY to 2.1**: Were the post-intervention variables that influenced selection likely to be associated with intervention?  2.3 **If Y/PY to 2.2**: Were the post-intervention variables that influenced selection likely to be influenced by the outcome or a cause of the outcome? |  | NA / Y / PY / PN / N / NI  NA / Y / PY / PN / N / NI |
|  | 2.4. Do start of follow-up and start of intervention coincide for most participants? | N; total intervention/control phase was 4 months. Notably, NH 5 implemented interventions “within a month after intervention phase”, while NH-4 did so “within 2 weeks after intervention phase”. | Y / PY / PN / N / NI |
|  | 2.5. **If Y/PY to 2.2 and 2.3, or N/PN to 2.4**: Were adjustment techniques used that are likely to correct for the presence of selection biases? | N | NA / Y / PY / PN / N / NI |
|  | **Risk of bias judgement** | Serious | Low / Moderate / Serious / Critical / NI |
|  | Optional: What is the predicted direction of bias due to selection of participants into the study? |  | Favours experimental / Favours comparator / Towards null /Away from null / Unpredictable |

| **Bias in classification of interventions** | | | |
| --- | --- | --- | --- |
|  | 3.1 Were intervention groups clearly defined? | Y – dependent on NH | Y / PY / PN / N / NI |
|  | 3.2 Was the information used to define intervention groups recorded at the start of the intervention? | Y | Y / PY / PN / N / NI |
|  | 3.3 Could classification of intervention status have been affected by knowledge of the outcome or risk of the outcome? | PN | Y / PY / PN / N / NI |
|  | **Risk of bias judgement** | Low | Low / Moderate / Serious / Critical / NI |
|  | Optional: What is the predicted direction of bias due to classification of interventions? |  | Favours experimental / Favours comparator / Towards null /Away from null / Unpredictable |

| **Bias due to deviations from intended interventions** | | | |
| --- | --- | --- | --- |
|  | **If your aim for this study is to assess the effect of assignment to intervention, answer questions 4.1 and 4.2** | |  |
|  | 4.1. Were there deviations from the intended intervention beyond what would be expected in usual practice? | N | Y / PY / PN / N / NI |
|  | 4.2. **If Y/PY to 4.1**: Were these deviations from intended intervention unbalanced between groups *and* likely to have affected the outcome? |  | NA / Y / PY / PN / N / NI |
|  | **If your aim for this study is to assess the effect of starting and adhering to intervention, answer questions 4.3 to 4.6** | |  |
|  | 4.3. Were important co-interventions balanced across intervention groups? | N | Y / PY / PN / N / NI |
|  | 4.4. Was the intervention implemented successfully for most participants? | Y | Y / PY / PN / N / NI |
|  | 4.5. Did study participants adhere to the assigned intervention regimen? | NI | Y / PY / PN / N / NI |
|  | 4.6. **If N/PN to 4.3, 4.4 or 4.5**: Was an appropriate analysis used to estimate the effect of starting and adhering to the intervention? | N | NA / Y / PY / PN / N / NI |
|  | **Risk of bias judgement** | Serious |  |
|  | Optional: What is the predicted direction of bias due to deviations from the intended interventions? |  |  |

| **Bias due to missing data** | | | |
| --- | --- | --- | --- |
|  | 5.1 Were outcome data available for all, or nearly all, participants? | Y | Y / PY / PN / N / NI |
|  | 5.2 Were participants excluded due to missing data on intervention status? | Y, but data for all RCFs were not included due to low recruitment. | Y / PY / PN / N / NI |
|  | 5.3 Were participants excluded due to missing data on other variables needed for the analysis? | Y; see above | Y / PY / PN / N / NI |
|  | 5.4 **If PN/N to 5.1, or Y/PY to 5.2 or 5.3**: Are the proportion of participants and reasons for missing data similar across interventions? | Y | NA / Y / PY / PN / N / NI |
|  | 5.5 **If PN/N to 5.1, or Y/PY to 5.2 or 5.3**: Is there evidence that results were robust to the presence of missing data? | Y | NA / Y / PY / PN / N / NI |
|  | **Risk of bias judgement** | Moderate | Low / Moderate / Serious / Critical / NI |
|  | Optional: What is the predicted direction of bias due to missing data? |  | Favours experimental / Favours comparator / Towards null /Away from null / Unpredictable |

| **Bias in measurement of outcomes** | | | |
| --- | --- | --- | --- |
|  | 6.1 Could the outcome measure have been influenced by knowledge of the intervention received? | PY; intervention consists of education and persuasion policies. Outcome measurement of appropriateness of AB is likely affected as physicians know they are under ‘watch’ during intervention period | Y / PY / PN / N / NI |
|  | 6.2 Were outcome assessors aware of the intervention received by study participants? | Y; Assessors were participants as well (since recording of AB used is part of the physician’s role) | Y / PY / PN / N / NI |
|  | 6.3 Were the methods of outcome assessment comparable across intervention groups? | Y | Y / PY / PN / N / NI |
|  | 6.4 Were any systematic errors in measurement of the outcome related to intervention received? | N | Y / PY / PN / N / NI |
|  | **Risk of bias judgement** | Moderate | Low / Moderate / Serious / Critical / NI |
|  | Optional: What is the predicted direction of bias due to measurement of outcomes? |  | Favours experimental / Favours comparator / Towards null /Away from null / Unpredictable |

| **Bias in selection of the reported result** | | | |
| --- | --- | --- | --- |
|  | Is the reported effect estimate likely to be selected, on the basis of the results, from... |  |  |
|  | 7.1. ... multiple outcome *measurements* within the outcome domain? | N | Y / PY / PN / N / NI |
|  | 7.2 ... multiple *analyses* of the intervention-outcome relationship? | N | Y / PY / PN / N / NI |
|  | 7.3 ... different *subgroups*? | N | Y / PY / PN / N / NI |
|  | **Risk of bias judgement** | Low | Low / Moderate / Serious / Critical / NI |
|  | Optional: What is the predicted direction of bias due to selection of the reported result? |  | Favours experimental / Favours comparator / Towards null /Away from null / Unpredictable |

| **Overall bias** | | | |
| --- | --- | --- | --- |
|  | **Risk of bias judgement** | Serious | Low / Moderate / Serious / Critical / NI |
|  | Optional: What is the overall predicted direction of bias for this outcome? |  | Favours experimental / Favours comparator / Towards null /Away from null / Unpredictable |

**Zabarsky et al 2008**

Responses underlined in green are potential markers for low risk of bias, and responses in red are potential markers for a risk of bias. Where questions relate only to sign posts to other questions, no formatting is used.

|  | **Signalling questions** | **Description** | **Response options** |
| --- | --- | --- | --- |
| **Bias due to confounding** | | | |
|  | 1.1 Is there potential for confounding of the effect of intervention in this study?  **If N/PN to 1.1:** the study can be considered to be at low risk of bias due to confounding and no further signalling questions need be considered | N; intervention applied to nurses, physicians etc. Unlikely to have confounds on patients themselves, though treatment may be affected (later category) | Y / PY / PN / N |
|  | **If Y/PY to 1.1**: determine whether there is a need to assess time-varying confounding: |  |  |
|  | 1.2. Was the analysis based on splitting participants’ follow up time according to intervention received?  **If N/PN**, answer questions relating to baseline confounding (1.4 to 1.6)  **If Y/PY**, go to question 1.3. |  | NA / Y / PY / PN / N / NI |
|  | 1.3. Were intervention discontinuations or switches likely to be related to factors that are prognostic for the outcome?  **If N/PN**, answer questions relating to baseline confounding (1.4 to 1.6)  **If Y/PY**, answer questions relating to both baseline and time-varying confounding (1.7 and 1.8) |  | NA / Y / PY / PN / N / NI |

|  | **Questions relating to baseline confounding only** | | |
| --- | --- | --- | --- |
|  | 1.4. Did the authors use an appropriate analysis method that controlled for all the important confounding domains? |  | NA / Y / PY / PN / N / NI |
|  | 1.5. **If Y/PY to 1.4**: Were confounding domains that were controlled for measured validly and reliably by the variables available in this study? |  | NA / Y / PY / PN / N / NI |
|  | 1.6. Did the authors control for any post-intervention variables that could have been affected by the intervention? |  | NA / Y / PY / PN / N / NI |
|  | **Questions relating to baseline and time-varying confounding** | |  |
|  | 1.7. Did the authors use an appropriate analysis method that controlled for all the important confounding domains and for time-varying confounding? |  | NA / Y / PY / PN / N / NI |
|  | 1.8. **If Y/PY to 1.7**: Were confounding domains that were controlled for measured validly and reliably by the variables available in this study? |  | NA / Y / PY / PN / N / NI |
|  | **Risk of bias judgement** | Low | Low / Moderate / Serious / Critical / NI |
|  | Optional: What is the predicted direction of bias due to confounding? |  | Favours experimental / Favours comparator / Unpredictable |

| **Bias in selection of participants into the study** | | | |
| --- | --- | --- | --- |
|  | 2.1. Was selection of participants into the study (or into the analysis) based on participant characteristics observed after the start of intervention?  **If N/PN to 2.1:** go to 2.4 | N | Y / PY / PN / N / NI |
|  | 2.2. **If Y/PY to 2.1**: Were the post-intervention variables that influenced selection likely to be associated with intervention?  2.3 **If Y/PY to 2.2**: Were the post-intervention variables that influenced selection likely to be influenced by the outcome or a cause of the outcome? |  | NA / Y / PY / PN / N / NI  NA / Y / PY / PN / N / NI |
|  | 2.4. Do start of follow-up and start of intervention coincide for most participants? | Y | Y / PY / PN / N / NI |
|  | 2.5. **If Y/PY to 2.2 and 2.3, or N/PN to 2.4**: Were adjustment techniques used that are likely to correct for the presence of selection biases? |  | NA / Y / PY / PN / N / NI |
|  | **Risk of bias judgement** | Low | Low / Moderate / Serious / Critical / NI |
|  | Optional: What is the predicted direction of bias due to selection of participants into the study? |  | Favours experimental / Favours comparator / Towards null /Away from null / Unpredictable |

| **Bias in classification of interventions** | | | |
| --- | --- | --- | --- |
|  | 3.1 Were intervention groups clearly defined? | Y – in nursing home, after a certain date (where intervention occurred) | Y / PY / PN / N / NI |
|  | 3.2 Was the information used to define intervention groups recorded at the start of the intervention? | Y | Y / PY / PN / N / NI |
|  | 3.3 Could classification of intervention status have been affected by knowledge of the outcome or risk of the outcome? | N | Y / PY / PN / N / NI |
|  | **Risk of bias judgement** | Low | Low / Moderate / Serious / Critical / NI |
|  | Optional: What is the predicted direction of bias due to classification of interventions? |  | Favours experimental / Favours comparator / Towards null /Away from null / Unpredictable |

| **Bias due to deviations from intended interventions** | | | |
| --- | --- | --- | --- |
|  | **If your aim for this study is to assess the effect of assignment to intervention, answer questions 4.1 and 4.2** | |  |
|  | 4.1. Were there deviations from the intended intervention beyond what would be expected in usual practice? | N | Y / PY / PN / N / NI |
|  | 4.2. **If Y/PY to 4.1**: Were these deviations from intended intervention unbalanced between groups *and* likely to have affected the outcome? |  | NA / Y / PY / PN / N / NI |
|  | **If your aim for this study is to assess the effect of starting and adhering to intervention, answer questions 4.3 to 4.6** | |  |
|  | 4.3. Were important co-interventions balanced across intervention groups? | PN: possible co-intervention with issuing of reference card vs educational intervention policy. | Y / PY / PN / N / NI |
|  | 4.4. Was the intervention implemented successfully for most participants? | Y – intervention at start of shift to ensure all nurses were given the intervention | Y / PY / PN / N / NI |
|  | 4.5. Did study participants adhere to the assigned intervention regimen? | Y – educational intervention | Y / PY / PN / N / NI |
|  | 4.6. **If N/PN to 4.3, 4.4 or 4.5**: Was an appropriate analysis used to estimate the effect of starting and adhering to the intervention? | NI | NA / Y / PY / PN / N / NI |
|  | **Risk of bias judgement** | Moderate |  |
|  | Optional: What is the predicted direction of bias due to deviations from the intended interventions? |  |  |

| **Bias due to missing data** | | | |
| --- | --- | --- | --- |
|  | 5.1 Were outcome data available for all, or nearly all, participants? | Y – intervention on nurses; outcome measured in patient treatments | Y / PY / PN / N / NI |
|  | 5.2 Were participants excluded due to missing data on intervention status? | N | Y / PY / PN / N / NI |
|  | 5.3 Were participants excluded due to missing data on other variables needed for the analysis? | N | Y / PY / PN / N / NI |
|  | 5.4 **If PN/N to 5.1, or Y/PY to 5.2 or 5.3**: Are the proportion of participants and reasons for missing data similar across interventions? |  | NA / Y / PY / PN / N / NI |
|  | 5.5 **If PN/N to 5.1, or Y/PY to 5.2 or 5.3**: Is there evidence that results were robust to the presence of missing data? |  | NA / Y / PY / PN / N / NI |
|  | **Risk of bias judgement** | Low | Low / Moderate / Serious / Critical / NI |
|  | Optional: What is the predicted direction of bias due to missing data? |  | Favours experimental / Favours comparator / Towards null /Away from null / Unpredictable |

| **Bias in measurement of outcomes** | | | |
| --- | --- | --- | --- |
|  | 6.1 Could the outcome measure have been influenced by knowledge of the intervention received? | Y: highly possible. It was not possible to avoid knowledge of intervention received because it was an educational intervention. | Y / PY / PN / N / NI |
|  | 6.2 Were outcome assessors aware of the intervention received by study participants? | Y | Y / PY / PN / N / NI |
|  | 6.3 Were the methods of outcome assessment comparable across intervention groups? | Y | Y / PY / PN / N / NI |
|  | 6.4 Were any systematic errors in measurement of the outcome related to intervention received? | N | Y / PY / PN / N / NI |
|  | **Risk of bias judgement** | Moderate | Low / Moderate / Serious / Critical / NI |
|  | Optional: What is the predicted direction of bias due to measurement of outcomes? |  | Favours experimental / Favours comparator / Towards null /Away from null / Unpredictable |

| **Bias in selection of the reported result** | | | |
| --- | --- | --- | --- |
|  | Is the reported effect estimate likely to be selected, on the basis of the results, from... |  |  |
|  | 7.1. ... multiple outcome *measurements* within the outcome domain? | N | Y / PY / PN / N / NI |
|  | 7.2 ... multiple *analyses* of the intervention-outcome relationship? | N | Y / PY / PN / N / NI |
|  | 7.3 ... different *subgroups*? | N | Y / PY / PN / N / NI |
|  | **Risk of bias judgement** | Low | Low / Moderate / Serious / Critical / NI |
|  | Optional: What is the predicted direction of bias due to selection of the reported result? |  | Favours experimental / Favours comparator / Towards null /Away from null / Unpredictable |

| **Overall bias** | | | |
| --- | --- | --- | --- |
|  | **Risk of bias judgement** | Moderate | Low / Moderate / Serious / Critical / NI |
|  | Optional: What is the overall predicted direction of bias for this outcome? |  | Favours experimental / Favours comparator / Towards null /Away from null / Unpredictable |

**Zimmerman et al 2014**

Responses underlined in green are potential markers for low risk of bias, and responses in red are potential markers for a risk of bias. Where questions relate only to sign posts to other questions, no formatting is used.

|  | **Signalling questions** | **Description** | **Response options** |
| --- | --- | --- | --- |
| **Bias due to confounding** | | | |
|  | 1.1 Is there potential for confounding of the effect of intervention in this study?  **If N/PN to 1.1:** the study can be considered to be at low risk of bias due to confounding and no further signalling questions need be considered | PY | Y / PY / PN / N |
|  | **If Y/PY to 1.1**: determine whether there is a need to assess time-varying confounding: |  |  |
|  | 1.2. Was the analysis based on splitting participants’ follow up time according to intervention received?  **If N/PN**, answer questions relating to baseline confounding (1.4 to 1.6)  **If Y/PY**, go to question 1.3. | N | NA / Y / PY / PN / N / NI |
|  | 1.3. Were intervention discontinuations or switches likely to be related to factors that are prognostic for the outcome?  **If N/PN**, answer questions relating to baseline confounding (1.4 to 1.6)  **If Y/PY**, answer questions relating to both baseline and time-varying confounding (1.7 and 1.8) |  | NA / Y / PY / PN / N / NI |

|  | **Questions relating to baseline confounding only** | | |
| --- | --- | --- | --- |
|  | 1.4. Did the authors use an appropriate analysis method that controlled for all the important confounding domains? | PY; however, proportions of residents younger than 65 was significantly different (21.6% in intervention, vs 8.0% in control). This may be a significant confound as younger residents are likely to be less prone to age-related issues. | NA / Y / PY / PN / N / NI |
|  | 1.5. **If Y/PY to 1.4**: Were confounding domains that were controlled for measured validly and reliably by the variables available in this study? | Y | NA / Y / PY / PN / N / NI |
|  | 1.6. Did the authors control for any post-intervention variables that could have been affected by the intervention? | N | NA / Y / PY / PN / N / NI |
|  | **Questions relating to baseline and time-varying confounding** | |  |
|  | 1.7. Did the authors use an appropriate analysis method that controlled for all the important confounding domains and for time-varying confounding? |  | NA / Y / PY / PN / N / NI |
|  | 1.8. **If Y/PY to 1.7**: Were confounding domains that were controlled for measured validly and reliably by the variables available in this study? |  | NA / Y / PY / PN / N / NI |
|  | **Risk of bias judgement** | M | Low / Moderate / Serious / Critical / NI |
|  | Optional: What is the predicted direction of bias due to confounding? |  | Favours experimental / Favours comparator / Unpredictable |

| **Bias in selection of participants into the study** | | | |
| --- | --- | --- | --- |
|  | 2.1. Was selection of participants into the study (or into the analysis) based on participant characteristics observed after the start of intervention?  **If N/PN to 2.1:** go to 2.4 | N | Y / PY / PN / N / NI |
|  | 2.2. **If Y/PY to 2.1**: Were the post-intervention variables that influenced selection likely to be associated with intervention?  2.3 **If Y/PY to 2.2**: Were the post-intervention variables that influenced selection likely to be influenced by the outcome or a cause of the outcome? |  | NA / Y / PY / PN / N / NI  NA / Y / PY / PN / N / NI |
|  | 2.4. Do start of follow-up and start of intervention coincide for most participants? | Y | Y / PY / PN / N / NI |
|  | 2.5. **If Y/PY to 2.2 and 2.3, or N/PN to 2.4**: Were adjustment techniques used that are likely to correct for the presence of selection biases? |  | NA / Y / PY / PN / N / NI |
|  | **Risk of bias judgement** | L | Low / Moderate / Serious / Critical / NI |
|  | Optional: What is the predicted direction of bias due to selection of participants into the study? |  | Favours experimental / Favours comparator / Towards null /Away from null / Unpredictable |

| **Bias in classification of interventions** | | | |
| --- | --- | --- | --- |
|  | 3.1 Were intervention groups clearly defined? | Y – Intervention/Control depends on which NH. | Y / PY / PN / N / NI |
|  | 3.2 Was the information used to define intervention groups recorded at the start of the intervention? | Y | Y / PY / PN / N / NI |
|  | 3.3 Could classification of intervention status have been affected by knowledge of the outcome or risk of the outcome? | N | Y / PY / PN / N / NI |
|  | **Risk of bias judgement** | L | Low / Moderate / Serious / Critical / NI |
|  | Optional: What is the predicted direction of bias due to classification of interventions? |  | Favours experimental / Favours comparator / Towards null /Away from null / Unpredictable |

| **Bias due to deviations from intended interventions** | | | |
| --- | --- | --- | --- |
|  | **If your aim for this study is to assess the effect of assignment to intervention, answer questions 4.1 and 4.2** | |  |
|  | 4.1. Were there deviations from the intended intervention beyond what would be expected in usual practice? | N | Y / PY / PN / N / NI |
|  | 4.2. **If Y/PY to 4.1**: Were these deviations from intended intervention unbalanced between groups *and* likely to have affected the outcome? |  | NA / Y / PY / PN / N / NI |
|  | **If your aim for this study is to assess the effect of starting and adhering to intervention, answer questions 4.3 to 4.6** | |  |
|  | 4.3. Were important co-interventions balanced across intervention groups? | Y. Intervention group received training and education which was not balanced with control group. Intervention group also received additional training in monthly meetings with QI staff.  However, as a result, no suitable comparator group. | Y / PY / PN / N / NI |
|  | 4.4. Was the intervention implemented successfully for most participants? | Y | Y / PY / PN / N / NI |
|  | 4.5. Did study participants adhere to the assigned intervention regimen? | Y | Y / PY / PN / N / NI |
|  | 4.6. **If N/PN to 4.3, 4.4 or 4.5**: Was an appropriate analysis used to estimate the effect of starting and adhering to the intervention? |  | NA / Y / PY / PN / N / NI |
|  | **Risk of bias judgement** | Low/Moderate |  |
|  | Optional: What is the predicted direction of bias due to deviations from the intended interventions? |  |  |

| **Bias due to missing data** | | | |
| --- | --- | --- | --- |
|  | 5.1 Were outcome data available for all, or nearly all, participants? | Y – data was available of all NHs. | Y / PY / PN / N / NI |
|  | 5.2 Were participants excluded due to missing data on intervention status? | N | Y / PY / PN / N / NI |
|  | 5.3 Were participants excluded due to missing data on other variables needed for the analysis? | N | Y / PY / PN / N / NI |
|  | 5.4 **If PN/N to 5.1, or Y/PY to 5.2 or 5.3**: Are the proportion of participants and reasons for missing data similar across interventions? |  | NA / Y / PY / PN / N / NI |
|  | 5.5 **If PN/N to 5.1, or Y/PY to 5.2 or 5.3**: Is there evidence that results were robust to the presence of missing data? |  | NA / Y / PY / PN / N / NI |
|  | **Risk of bias judgement** | L | Low / Moderate / Serious / Critical / NI |
|  | Optional: What is the predicted direction of bias due to missing data? |  | Favours experimental / Favours comparator / Towards null /Away from null / Unpredictable |

| **Bias in measurement of outcomes** | | | |
| --- | --- | --- | --- |
|  | 6.1 Could the outcome measure have been influenced by knowledge of the intervention received? | Y – prescribers were given additional training on prescribing guidelines and 12 common situations when use of antibiotics were unneeded. | Y / PY / PN / N / NI |
|  | 6.2 Were outcome assessors aware of the intervention received by study participants? | N – Research nurses who obtained information were blind to study aims and conditions | Y / PY / PN / N / NI |
|  | 6.3 Were the methods of outcome assessment comparable across intervention groups? | Y | Y / PY / PN / N / NI |
|  | 6.4 Were any systematic errors in measurement of the outcome related to intervention received? | N | Y / PY / PN / N / NI |
|  | **Risk of bias judgement** | L | Low / Moderate / Serious / Critical / NI |
|  | Optional: What is the predicted direction of bias due to measurement of outcomes? |  | Favours experimental / Favours comparator / Towards null /Away from null / Unpredictable |

| **Bias in selection of the reported result** | | | |
| --- | --- | --- | --- |
|  | Is the reported effect estimate likely to be selected, on the basis of the results, from... |  |  |
|  | 7.1. ... multiple outcome *measurements* within the outcome domain? | N | Y / PY / PN / N / NI |
|  | 7.2 ... multiple *analyses* of the intervention-outcome relationship? | N | Y / PY / PN / N / NI |
|  | 7.3 ... different *subgroups*? | N. For all three, standard analyses and outcome measurements were performed. | Y / PY / PN / N / NI |
|  | **Risk of bias judgement** | L | Low / Moderate / Serious / Critical / NI |
|  | Optional: What is the predicted direction of bias due to selection of the reported result? |  | Favours experimental / Favours comparator / Towards null /Away from null / Unpredictable |

| **Overall bias** | | | |
| --- | --- | --- | --- |
|  | **Risk of bias judgement** | M | Low / Moderate / Serious / Critical / NI |
|  | Optional: What is the overall predicted direction of bias for this outcome? |  | Favours experimental / Favours comparator / Towards null /Away from null / Unpredictable |


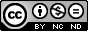


This work is licensed under a [Creative Commons Attribution-NonCommercial-NoDerivatives 4.0 International License](http://creativecommons.org/licenses/by-nc-nd/4.0/).

**Figure: Displaying the number of studies allocated each risk of bias score from the ROBINS-I tool for non randomised study designs.**

**Cochrane Risk of Bias Assessment of RCTs only**

**Table: Displaying the percentage of studies allocated to each risk of bias domain for the Cochrane Risk of Bias tool.**

| **Cochran toll domain** | **Low** | **Unclear** | **High/Serious** |
| --- | --- | --- | --- |
| Overall Risk | 40% | 20% | 40% |
| Random Sequence Generation (RCT) | 80% | 20% | 0% |
| Allocation Concealment (RCT) | 80% | 20% | 0% |
| Blinding of Participants and Personnel (RCT) | 80% | 20% | 0% |
| Blinding of Outcome Assessment (RCT) | 100% | 0% | 0% |
| Incomplete Outcome Data (RCT) | 40% | 20% | 40% |
| Selective Reporting (RCT) | 100% | 0% | 0% |

Figure: Displaying the percentage of studies allocated each risk of bias score from the Cochrane Risk of Bias tool for RCTs.

Figure: Displaying the combined risk of bias analysis for both RCTs and non-randomised study designs applying both the Cochrane risk of bias tool and the ROBINS-I tool, where appropriate.

The horizontal axis shows the percentage of studies allocated to each score for risk of bias.

Blank spaces in the bars represent where not all domains of risk assessment were applicable to all studies contingent on what tool was applied to assess risk of bias.

**Supplement 4: BCT-COM-B mapping exercise**

Table 1. Mapping exercise to demonstrate theoretical link between CO-B Model of Behaviour and BCTs delivered in interventions included in review

| **COM-B Construct** | **Definition*** | **Intervention types identified in included review studies** | **Frequency of intervention types (n=number of interventions)** | **BCTs identified in included review studies** | **Frequency of BCTs (n=number of interventions)** |
| --- | --- | --- | --- | --- | --- |
| Capability | | | | | |
| Physical | Having the physical skills, strength or stamina to perform the behaviour | Enablement | 19 | Instruction on how to perform the behaviour | 18 |
|  |  | Training | 9 | Demonstration of the behaviour | 4 |
|  |  |  |  | Problem solving | 4 |
| Psychological | Having the knowledge or psychological skills, strength or stamina to engage in engage in the necessary mental processes in order to perform the behaviour | Enablement | 19 | Feedback on Behaviour | 9 |
|  |  |  |  | Discrepancy between behaviour and goal | 4 |
|  |  | Education | 16 | Instruction on how to perform the behaviour | 18 |
|  |  |  |  | Information about health consequences | 5 |
|  |  | Training | 9 | Prompts/cues | 7 |
|  |  |  |  | Problem solving | 4 |
|  |  |  |  | Self-monitoring behaviour | 4 |
|  |  |  |  | Self-monitoring outcome(s) of behaviour | 1 |
| Opportunity | | | | | |
| Physical | Opportunities afforded by the environment involving time, resources, locations, cues, physical ‘affordance’ | Enablement | 19 | Prompts/cues | 7 |
|  |  | Training | 9 | Adding objects to the environment | 13 |
|  |  |  |  | Problem solving | 4 |
|  |  | Environmental restructuring | 17 | Social support (practical) | 6 |
|  |  |  |  | Restructuring the social environment | 8 |
|  |  | Restriction | 1 | Restructuring the physical environment | 2 |
| Social | Opportunities afforded by the interpersonal influences, social cues and cultural norms that influence the way that we think about things | Enablement | 19 | Monitoring of behaviour by others without feedback | 2 |
|  |  | Modelling | 4 | Social support (practical) | 6 |
|  |  | Environmental restructuring | 17 | Restructuring the social environment | 8 |
|  |  | Restriction | 1 |  |  |
| Motivation | | | | | |
| Reflective | Irreflective processes involving plans (self-conscious intentions) and evaluations (beliefs about what is good or bad) | Education | 16 | Discrepancy between behaviour and goal | 4 |
|  |  |  |  | Instruction on how to perform the behaviour | 18 |
|  |  | Incentivisation | 1 | Information about health consequences | 5 |
|  |  |  |  | Demonstration of the behaviour | 4 |
|  |  | Persuasion | 12 | Problem solving | 4 |
|  |  |  |  | Material incentive (behaviour) | 1 |
|  |  |  |  | Credible source | 12 |
|  |  |  |  | Review behaviour goal(s) | 1 |
| Automatic | Automatic processes involving emotional reactions, desires ( wants and needs), impulses, inhibitions, drive states and reflex responses | Enablement | 19 | Feedback on behaviour | 9 |
|  |  | Training | 9 | Prompts/cues | 7 |
|  |  | Modelling | 4 | Monitoring of behaviour by others without feedback | 2 |
|  |  | Environmental restructuring | 17 |  |  |
|  |  | Incentivisation | 1 | Material incentive (behaviour) | 1 |
|  |  | Persuasion | 12 |  |  |

*Definitions of COM-B constructs from: Michie S, Atkins L, West R. The behaviour change wheel: a guide to designing interventions. Needed: physician leaders. 2014;26.

*---Please note- ‘Feedback on outcome(s) of behaviour’ has no known confirmed or inconclusive links to a model of behaviour as presented by the Theory and Techniques Tool at the time of this review*
